# Supplementary material for: Imaging the response to DNA damage in heterochromatin domains reveals core principles of heterochromatin maintenance
Source: Nat Commun. 2021 Apr 23;12:2428. doi: 10.1038/s41467-021-22575-5 (PMC8065061; doi:10.1038/s41467-021-22575-5)
Supplement: Supplementary file 1 — Supplementary Information [file 41467_2021_22575_MOESM1_ESM.pdf]

Supplementary Figure 1

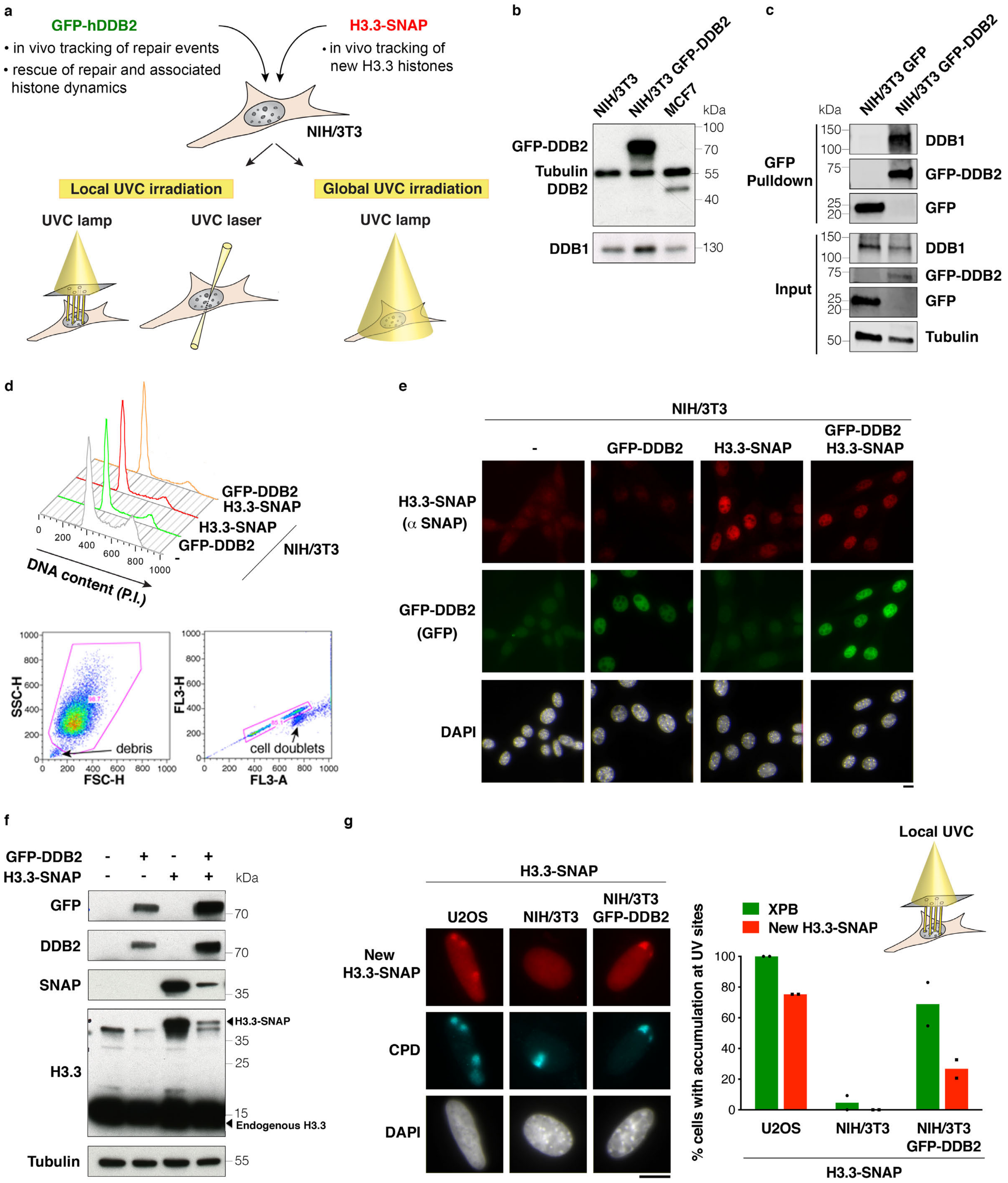

Supplementary Figure 1. Mammalian cellular model for studying heterochromatin maintenance in response to UV damage.

(**a**) Engineered NIH/3T3 stable cell lines permit to track DNA repair events and to follow H3.3 histone deposition into chromatin in cells exposed to global or local UVC irradiation.
(**b**) Western blot analysis showing the levels of ectopically expressed GFP-hDDB2 in NIH/3T3 cells compared to endogenous hDDB2 in MCF7 cells. The overexpression of GFP-hDDB2 results in a slight increase in endogenous DDB1 in NIH/3T3 cells.
(**c**) Ectopically expressed GFP-hDDB2 pulls down endogenous DDB1 in NIH/3T3 cells.
(**d**) Cell cycle distribution analysed by flow cytometry in the NIH/3T3 stable cell lines (top graph) and the corresponding gating strategy to gate out debris and cell doublets (bottom graphs). FSC-H, forward scatter-height; SSC-H, side scatter-height; FL3-A/-H, red fluorescent channel-area/-height; P.I.: Propidium Iodide.
(**e**) H3.3-SNAP and GFP-DDB2 expression analysed by fluorescence microscopy in the indicated cell lines.
(**f**) Total cell extracts of NIH/3T3 stable cell lines (same as in c) analysed by western blot with the indicated antibodies. The top band detected by the H3.3 antibody corresponds to H3.3-SNAP. Tubulin is used as a loading control.
(**g**) New H3.3 histone deposition (red) at UVC damage sites (CPD) analysed by immunofluorescence in the indicated cell lines 45 min after local UVC irradiation through micropore filters. Histograms represent the fraction of cells showing new H3.3 histone accumulation at UV damage sites (red bars). Recruitment of the repair factor XPB to UV damage sites was analysed by immunofluorescence 30 min after local UVC irradiation in the same cell lines and plotted on the same histogram (green bars). Data are presented as mean values from two independent experiments scoring 150 cells in each experiment. Similar results were obtained in two independent experiments (b, c, e, f). Scale bars, 10 µm. Source data are provided as a Source Data file.

Supplementary Figure 2

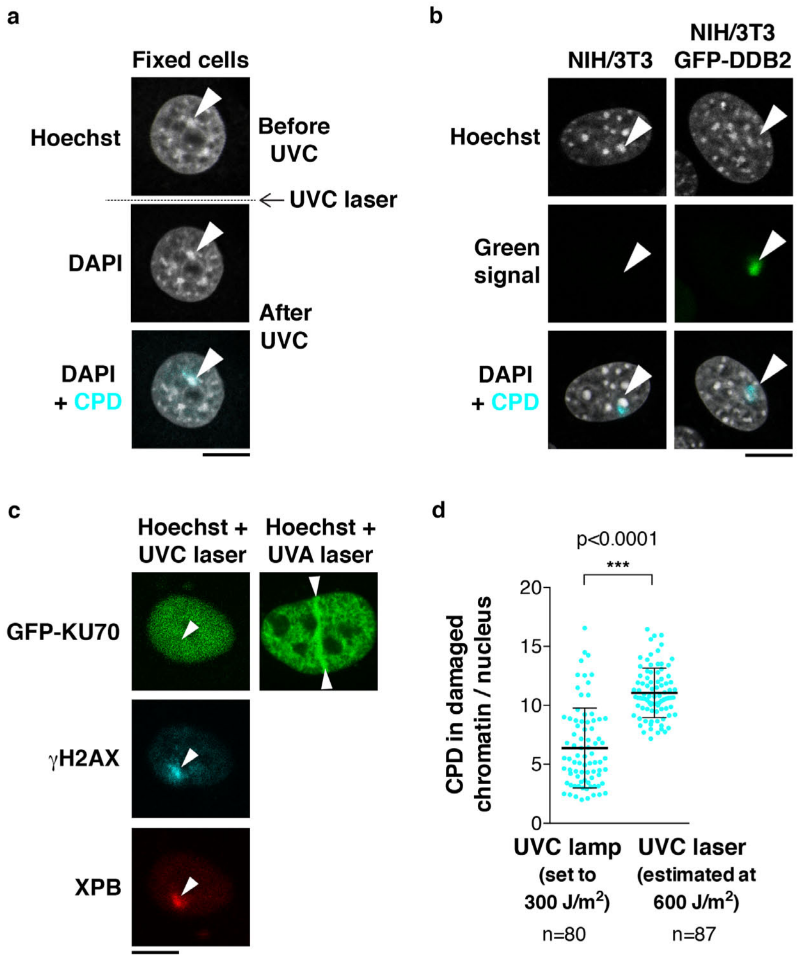

Supplementary Figure 2. Characterization of UVC laser damage combined with Hoechst staining.

(**a**) No detectable bleaching of the Hoechst signal by the UVC laser upon irradiation in paraformaldehyde-fixed NIH/3T3 GFP-DDB2 cells.
(**b**) No switch from blue to green signal upon UVC laser irradiation in Hoechst-labeled NIH/3T3 nuclei. Same cells as in Fig. 3b.
(**c**) No induction of DNA double-strand breaks (DSBs): the recruitment of the DSB repair factor KU70 is observed to sites of UVA but not UVC laser damage in Hoechst-stained U2OS cells. Cells were fixed 30 min post laser damage.
(**d**) UVC laser micro-irradiation in Hoechst-labeled NIH/3T3 cells generates 2-fold more UV photoproducts (CPD) as compared to a 300 J/m^2 irradiation with a UVC lamp through micropore filters. The UVC dose delivered by the UVC laser can thus be estimated at 600 J/m^2. Data are presented as mean values +/- s.d. from n cells scored in four (UVC lamp) and six (UVC laser) independent experiments. Statistical significance is calculated via two-sided Student's t-test with Welch's correction. Similar results were obtained in two independent experiments (a-c). All microscopy images are confocal sections. Scale bars, 10 µm. Source data are provided as a Source Data file.

Supplementary Figure 3

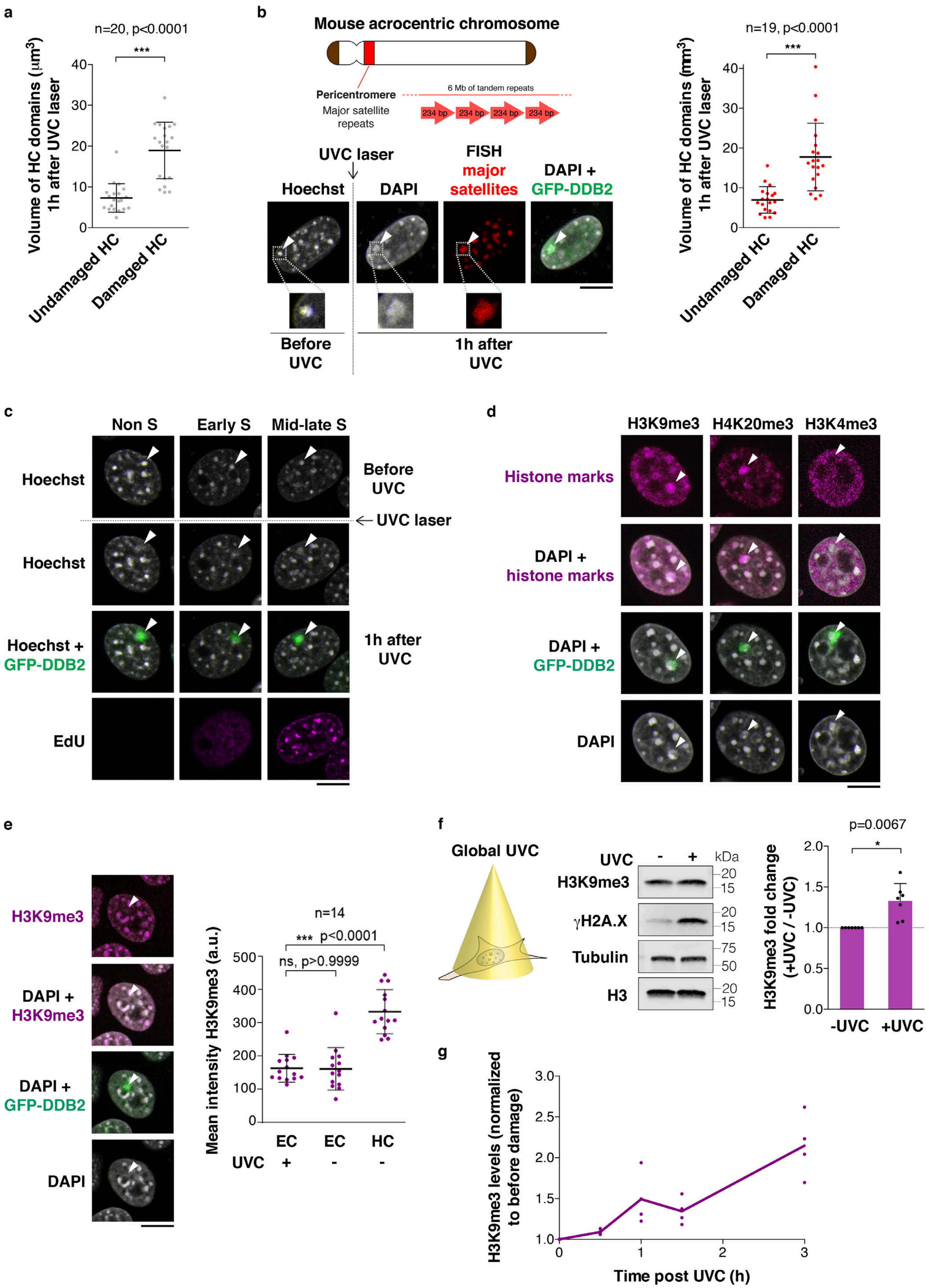

Supplementary Figure 3. Decompaction and histone modification changes in UV-damaged pericentric heterochromatin domains.

(**a**) Decompaction of UVC-damaged chromocenters as compared to undamaged chromocenters 1h after UVC laser micro-irradiation in NIH/3T3 GFP-DDB2 cells. The scatter plot shows the volume of heterochromatin (HC) domains based on DAPI staining, measured on reconstructed 3D images (damaged and undamaged heterochromatin domains are from the same nuclei).
(**b**) Schematic representation of major satellite repeats in pericentric regions of mouse chromosomes. Heterochromatin decompaction following UVC laser micro-irradiation is visualized by DNA-FISH of major satellite DNA sequences in NIH/3T3 GFP-DDB2 cells. The scatter plot shows the volume of pericentric heterochromatin domains based on DNA FISH staining, measured on reconstructed 3D images (damaged and undamaged heterochromatin domains are from the same nuclei).
(**c**) Decompaction of UV-damaged heterochromatin (white arrowheads) 1h after UVC laser damage in NIH/3T3 GFP-DDB2 cells. Cell cycle stages were defined based on staining of replication foci with Ethynyl-deoxyUridine (EdU).
(**d**) H3K9me3, H4K20me3 and H3K4me3 in damaged heterochromatin (white arrowheads) analysed by immunofluorescence 1h after UVC laser micro-irradiation in NIH/3T3 GFP-DDB2 cells. H3K9me3 and H4K20me3 are heterochromatin-specific modifications associated with transcriptional silencing while H3K4me3 is a transcriptionally active histone mark used as negative control.
(**e**) H3K9me3 in damaged euchromatin (white arrowheads) analysed as in Fig. 1c. The scatter plot shows H3K9me3 levels measured on reconstructed 3D images in damaged euchromatin domains (EC +UVC) compared to undamaged euchromatin (EC –UVC) and heterochromatin (HC –UVC) in the same nucleus.
(**f**) H3K9me3 levels analysed by western blot 1h30 after global UVC irradiation. Tubulin, loading control; γH2A.X, damage marker. The bar graph represents H3K9me3 abundance in damaged/undamaged conditions.
(**g**) H3K9me3 levels analysed by western blot at the indicated time points following global UVC irradiation.
Data are presented as mean values +/- s.d. from seven (f) and four (g) experiments or from n cells scored in seven (a), three (b) or two (e) independent experiments. Statistical significance is calculated via two-sided Student's t-test with Welch's correction when necessary (a, b, f). Multiple comparisons are performed by one-way ANOVA with Bonferroni post-test (e). Similar results were obtained in three (b) and two (d) independent experiments. Scale bars, 10 μm. Zoomed in views of heterochromatin domains (x2.6). Source data are provided as a Source Data file.

Supplementary Figure 4

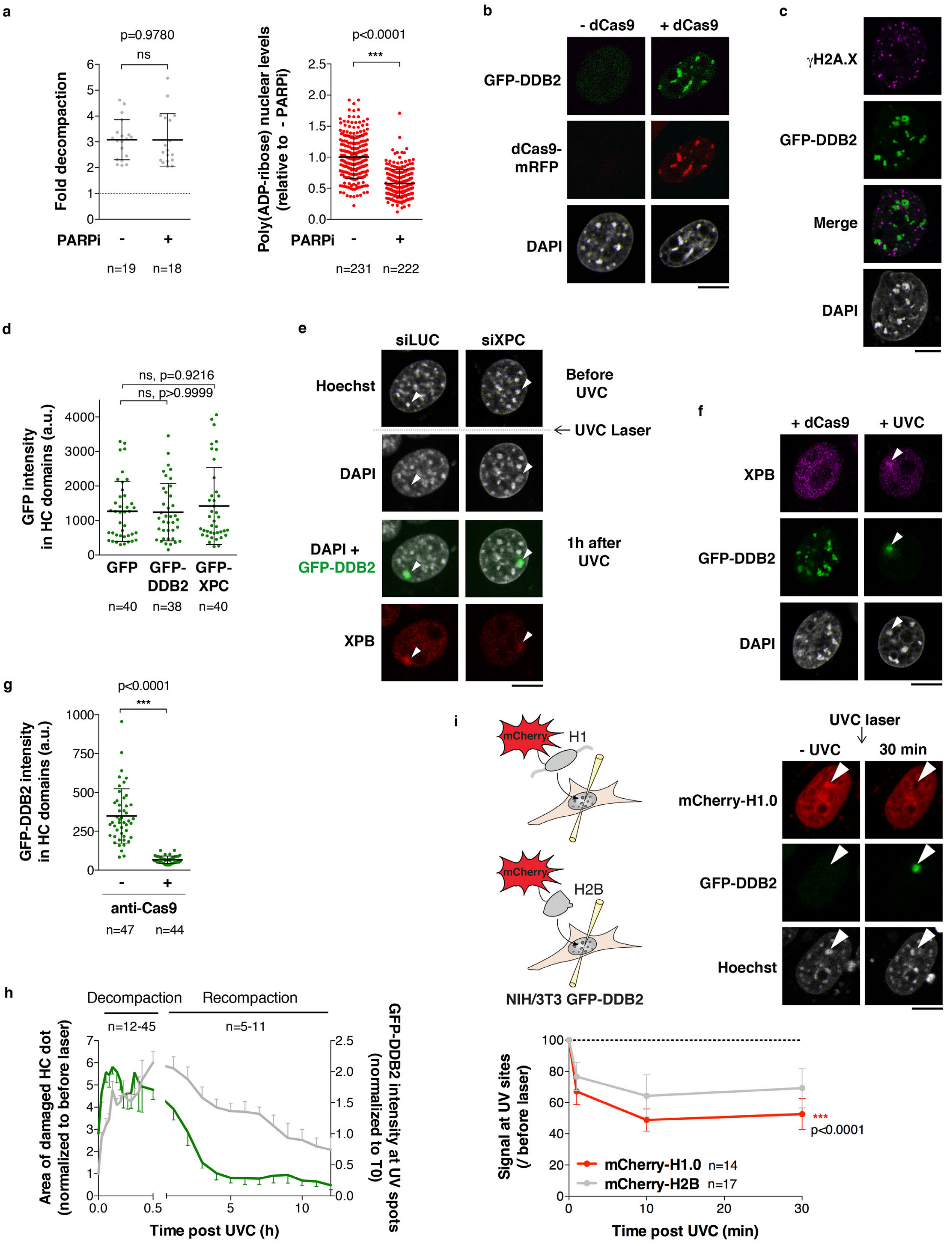

Supplementary Figure 4. DDB2-dependent regulation of heterochromatin compaction.

(**a**) Heterochromatin decompaction 1h post UVC laser damage measured as in Fig. 2a in NIH/3T3 GFP-DDB2 cells upon treatment with PARP inhibitor (PARPi). The efficiency of PARP inhibition is controlled by immunostaining of Poly(ADP-ribose).
(**b**) Confocal sections showing the tethering of GFP-DDB2 to pericentric heterochromatin domains of NIH/3T3 GFP-DDB2 cells in the presence of catalytically dead Cas9 (dCas9).
(**c**) Confocal sections showing no overlap between the DNA damage marker γH2A.X and GFP-DDB2 tethered to pericentric heterochromatin.
(**d**, **g**) GFP intensity levels in heterochromatin (HC) domains quantified on reconstructed 3D images corresponding to Fig. 2c (d) and Fig. 2d (g).
(**e**) Heterochromatin decompaction 1h post UVC laser damage in NIH/3T3 GFP-DDB2 cells treated with the indicated siRNAs (siLUC, control). XPC knockdown efficiency is controlled by immunostaining for XPB, whose recruitment to UV damage is promoted by XPC.
(**f**) Confocal sections showing that dCas9-mediated tethering of GFP-DDB2 to pericentric heterochromatin (+dCas9) does not result in XPB recruitment contrary to local UVC irradiation (+UVC). The white arrowhead points to the damaged chromocenter.
(**h**) Heterochromatin (HC) compaction changes (same data as in Figure 1b) and DDB2 recruitment kinetics upon UVC laser micro-irradiation analysed by live imaging in NIH/3T3 GFP-DDB2 cells stained with Hoechst.
(**i**) Scheme of the experiment for the detection of mCherry-tagged H1 and H2B in live NIH/3T3 GFP-DDB2 cells exposed to UVC laser damage. The levels of H1.0 and H2B are measured in UVC-damaged regions, identified by GFP-DDB2 accumulation (white arrowheads), relative to the whole nucleus at the indicated time points after laser damage. Results normalized to before laser damage are presented on the graphs.
Data are presented as mean values +/- s.d. from n cells scored in at least three independent experiments. Statistical significance is calculated via two-sided Student's t-test with Welch's correction when necessary (a, g). Multiple comparisons are performed by one-way ANOVA with Bonferroni post-test (d). Comparison of mcherry-H1.0 and -H2B signal loss is based on non-linear regression with a polynomial quadratic model (i). Similar results were obtained in two independent experiments (b, c, e, f). Scale bars, 10 μm. Source data are provided as a Source Data file.

Supplementary Figure 5

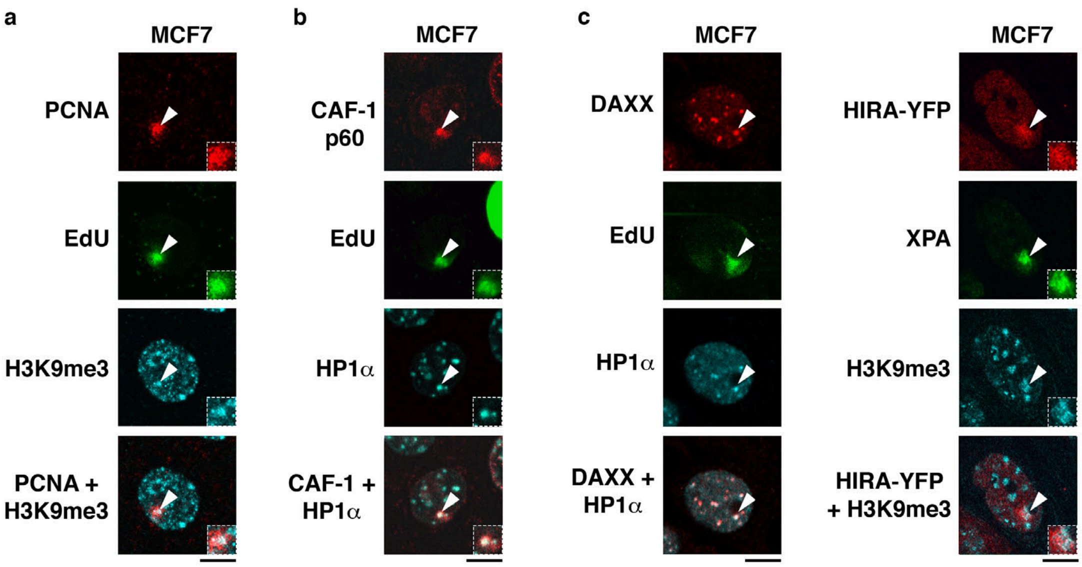

Supplementary Figure 5. Validation in human MCF7 cells.

(**a-c**) Recruitment of the repair factor PCNA (a) and of the histone chaperones CAF-1 (p60 subunit) (b), DAXX and HIRA (c) to UVC damaged heterochromatin domains (white arrowheads) analysed 30 min (HIRA) or 1h30 (PCNA, CAF-1, DAXX) after local UVC irradiation through micropore filters in MCF7 cells. PCNA, CAF-1 and DAXX are detected by immunofluorescence and HIRA upon transfection of HIRA-YFP. Constitutive heterochromatin is revealed by H3K9me3 or HP1α immunostaining. Sites of UVC damage repair are marked by Ethynyl-deoxyUridine (EdU, repair synthesis) or by immunodetection of the repair factor XPA. Insets show zoomed in views of heterochromatin domains (x1.8). All microscopy images are confocal sections. Similar results were obtained in at least two independent experiments. Scale bars, 10 µm. Source data are provided as a Source Data file.

Supplementary Figure 6

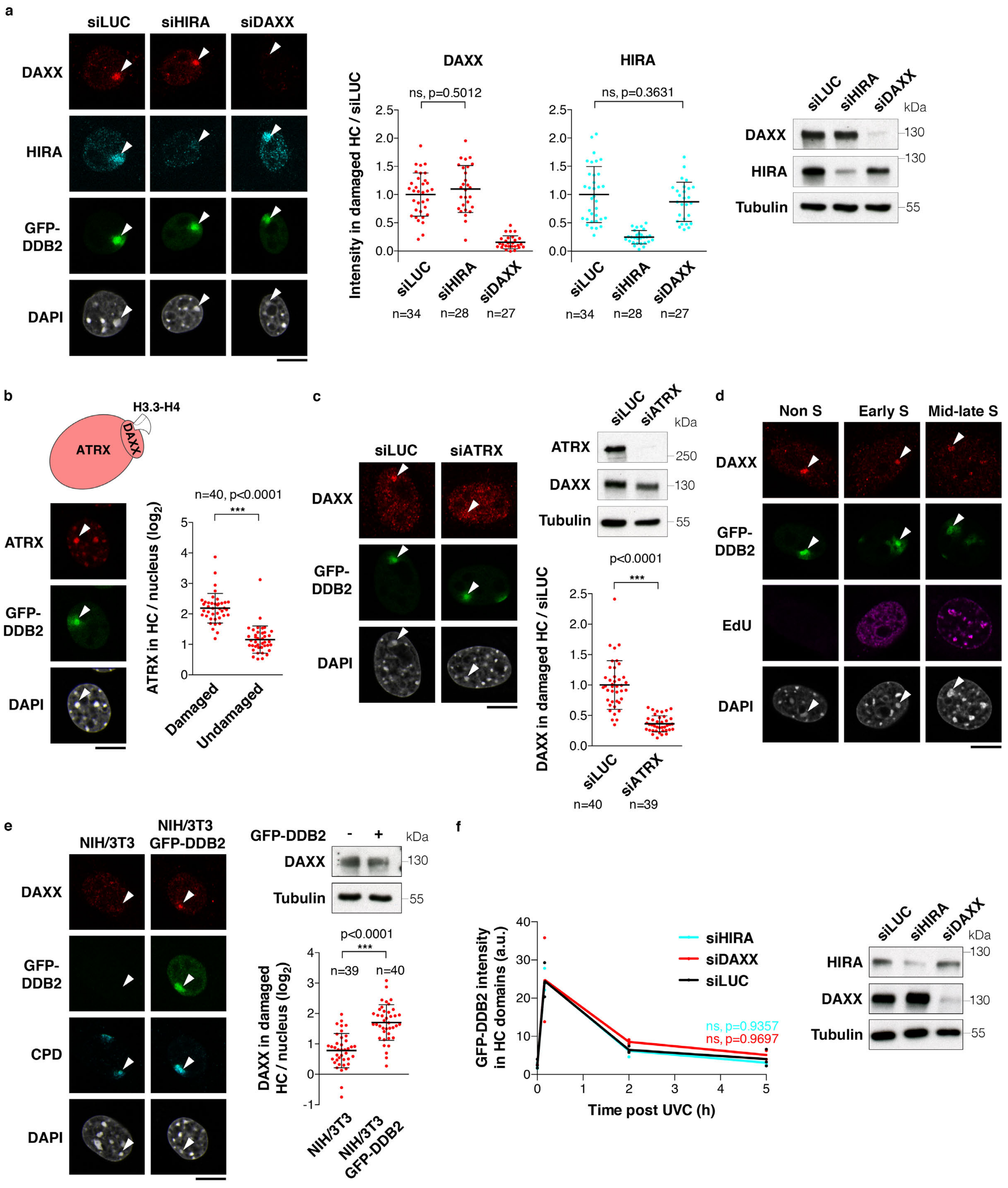

Supplementary Figure 6. DAXX accumulation in UVC-damaged heterochromatin.

(**a**) Recruitment of DAXX and HIRA chaperones to damaged heterochromatin (white arrowheads) analysed by immunofluorescence 1h30 after local UVC irradiation through micropore filters in NIH/3T3 GFP-DDB2 cells treated with the indicated siRNAs (siLUC, control). siRNA efficiencies are controlled by western blot (Tubulin, loading control).
(**b**) Recruitment of ATRX to damaged heterochromatin (white arrowheads) analysed by immunofluorescence 1h30 after local UVC irradiation in NIH/3T3 GFP-DDB2 cells.
(**c**) DAXX recruitment to damaged heterochromatin upon ATRX knock-down (siLUC, control) 1h30 after local UVC irradiation in NIH/3T3 GFP-DDB2 cells.
(**d**) Recruitment of DAXX to damaged heterochromatin (white arrowheads) 1h30 after local UVC irradiation in NIH/3T3 GFP-DDB2 cells. Cell cycle stages were defined based on staining of replication foci with Ethynyl-deoxyUridine (EdU). Similar results were obtained in two independent experiments.
(**e**) Recruitment of DAXX to damaged heterochromatin analysed in the indicated cell lines 1h30 after local UVC irradiation. DAXX total levels are shown on the western blot (Tubulin, loading control).
(**f**) Kinetic analysis of GFP-DDB2 release from UVC-damaged chromocenters analysed by fluorescence microscopy in NIH/3T3 GFP-DDB2 cells treated with the indicated siRNAs and exposed to global UVC irradiation in two (siDAXX) or three (siLUC, siHIRA) independent experiments. Knockdown efficiencies are controlled by western blot (Tubulin, loading control).
The scatter plots show DAXX, ATRX and HIRA levels in damaged heterochromatin (HC) normalized to the corresponding siLUC experiment (a, c) or log2 fold enrichments compared to the whole nucleus (b, e). Data are presented as mean values +/- s.d. from n cells scored in three independent experiments. Multiple comparisons are performed by one-way ANOVA with Bonferroni post-test (a). Statistical significance in (b, c, e) is calculated via two-sided Student's t-test with Welch's correction when necessary. Comparisons of GFP-DDB2 release kinetics are based on non-linear regression with a polynomial quadratic model (f). All microscopy images are confocal sections. Scale bars, 10 µm. Source data are provided as a Source Data file.

Supplementary Figure 7

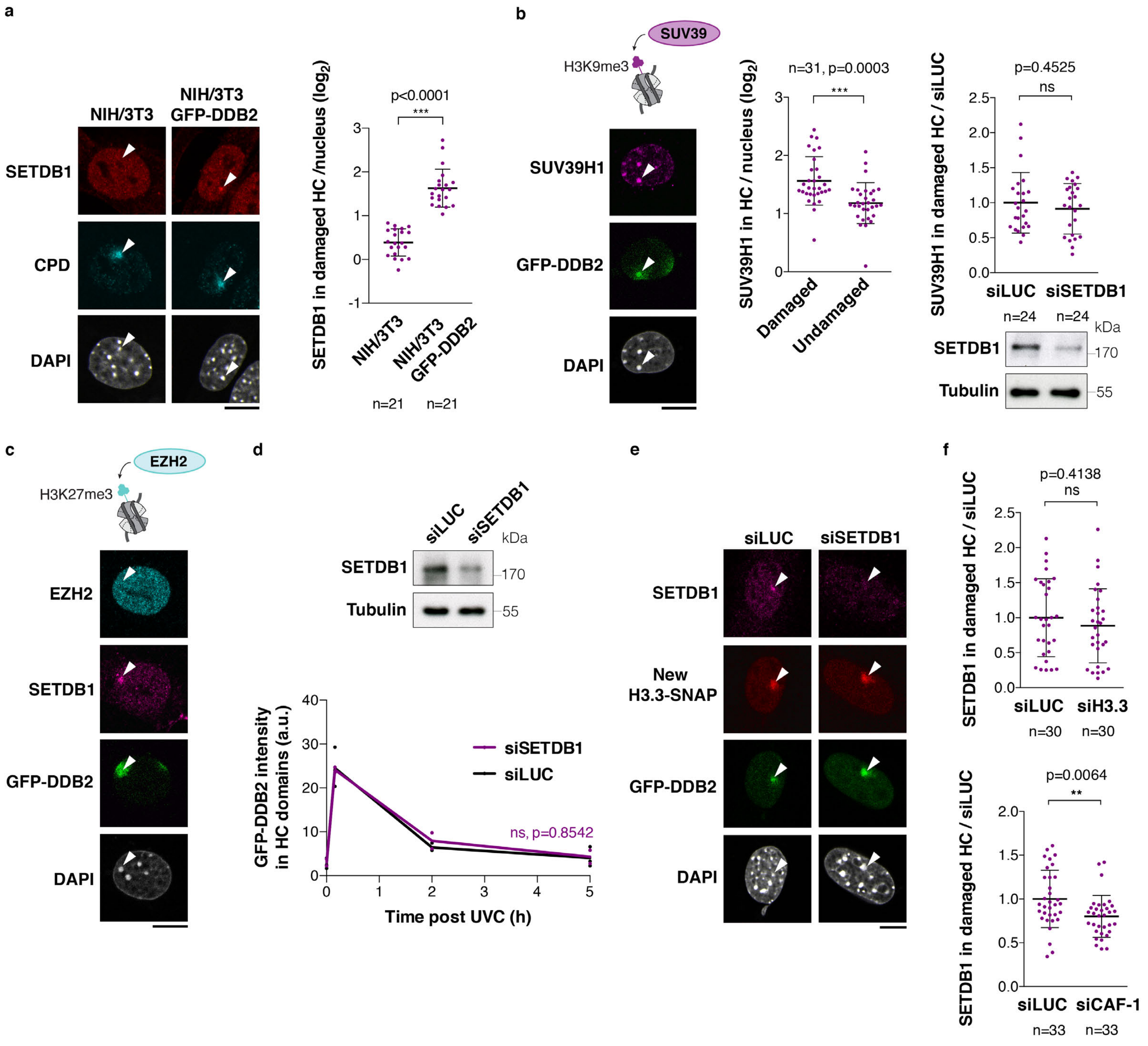

Supplementary Figure 7. Recruitment of histone methyltransferases to UVC-damaged heterochromatin.

(**a**) SETDB1 recruitment to UVC-damaged heterochromatin (HC) analyzed by immunofluorescence 1h30 after local UVC irradiation through micropore filters in the indicated cell lines.
(**b, c**) Recruitment of the histone methyltransferases SUV39H1 (b) and EZH2 (c) to damaged heterochromatin (HC) (white arrowheads) analysed by immunofluorescence 1h30 after local UVC irradiation through micropore filters in NIH/3T3 GFP-DDB2 cells. SETDB1 knockdown is controlled by western-blot (Tubulin, loading control).
(**d**) Kinetic analysis of GFP-DDB2 release from UVC-damaged chromocenters analysed by fluorescence microscopy in NIH/3T3 GFP-DDB2 cells treated with the indicated siRNAs and exposed to global UVC irradiation in two (siSETDB1) or three (siLUC) independent experiments. siLUC data are the same as in Supplementary Fig. 6f. Knockdown efficiencies are controlled by western blot (Tubulin, loading control).
(**e**) Accumulation of newly synthesized H3.3 histones in UVC-damaged heterochromatin regions (white arrowheads) upon SETDB1 knockdown (siLUC, control) analysed in NIH/3T3 GFP-DDB2 H3.3-SNAP cells 1h30 after local UVC irradiation through micropore filters.
(**f**) Scatter plots representing log2 fold enrichments of SETDB1 in damaged heterochromatin normalized to the corresponding siLUC experiment upon knock down of H3.3 or CAF-1 (p150 subunit).
Data are presented as mean values +/- s.d. from n cells scored in three independent experiments. Statistical significance in (a, b, f) is calculated via two-sided Student's t-test with Welch's correction when necessary. Comparisons of GFP-DDB2 release kinetics are based on non-linear regression with a polynomial quadratic model (d). Similar results were obtained in two independent experiments (c, e). All microscopy images are confocal sections. Scale bars, 10 µm. Source data are provided as a Source Data file.

SUPPLEMENTARY TABLES

Supplementary Table 1: Stable cell lines.

Ectopically expressed proteins are of human origin and are more than 80% similar to mouse proteins. Antibiotics: Hygromycin, G418 (Euromedex)

**Stable cell lines**	**Selection antibiotics**
NIH/3T3 GFP-DDB2	200 µg/ml Hygromycin
NIH/3T3 GFP-DDB2 H3.3-SNAP	200 µg/ml Hygromycin + 250 µg/ml G418
NIH/3T3 H3.3-SNAP	100 µg/ml G418
U2OS H3.3-SNAP	100 µg/ml G418

Supplementary Table 2: siRNA sequences.

**Designation**	**Target species**	**Target sequence**	**Working conditions**
**siATRX**	Mouse	$^{5'}$GTACAGAAATCTCGCTCAA$^{3'}$	50 nM – 72 h
**siCAF-1 p150**	Mouse	$^{5'}$AAGGAGAAGGCGGAGAAGCAG$^{3'}$	30 nM – 48 h
**siDAXX**	Mouse	$^{5'}$TGACCTTACAAACACTGAA$^{3'}$	50 nM – 72 h
**siH3.3**	Mouse	1:1 combination of siH3.3A: $^{5'}$CTACAAAAGCCGCTCGCAA$^{3'}$ and siH3.3B: $^{5'}$GCCAAGAGAGTCACCATCA$^{3'}$	50 nM – 48 to 72 h
**siHIRA**	Mouse	$^{5'}$ GGAAGGTTGTGATCTGGAA $^{3'}$	50 nM – 72 h
**siLUC (Luciferase)**	Firefly	$^{5'}$CGTACGCGGAATACTTCGA$^{3'}$	50 nM – 48 to 72 h
**siSETDB1**	Mouse	$^{5'}$GCGCAGAGTTAACCGCAAA$^{3'}$	50 nM – 72 h
**siSETDB1**	Mouse	$^{5'}$CCCGAGGCTTTGCTCTTAAAT$^{3'}$	50 nM – 72 h
**siSUV39H1/2**	Mouse	$^{5'}$ACCTCTTTGACCTGGACTA$^{3'}$	50 nM – 72 h
**siXPC**	Mouse	$^{5'}$GCCAGTGGCTTCTATCGAA$^{3'}$	50 nM – 72 h
**siXPG**	Mouse	$^{5'}$TGATGATAACGATGAGAAA$^{3'}$	50 nM – 72 h

Supplementary Table 3: Plasmids.

Human proteins expressed in mouse cells share around 90% identity with their mouse counterparts.

Plasmid	Construct details	Reference/Provider
**anti-Cas9 (pJH376-AcrIIA4)**	Bacteriophage AcrIIA4 sequence inserted into pcDNA3.1(+)	Gift from Joseph Bondy-Denomy. Addgene plasmid #86842 [101]
**GBP-dCas9-mRFP**	GFP-binding nanobody (GBP) and mRFP coding sequences cloned into pCAG-dCas9	Gift from Sebastian Butlmann [55]
**GFP**	pEGFP-C1	Clontech #6084-1
**GFP-DDB2**	Human *DDB2* coding sequence (Montpellier Genomic Collections) subcloned into GFP-XPC plasmid replacing XPC	[51]
**GFP-XPC**	cDNA encoding GFP-human XPC cloned into pIREShyg vector (Clontech)	Gift from Ryotaro Nishi [102]
**H3.3-SNAP**	Human *H3F3B* coding sequence cloned into pSNAPm (New England Biolabs)	[103]
**HIRA-YFP**	Human *HIRA* sequence edited by Genscript and subcloned into pEYFP-N1 (Clontech)	[50]
**GFP-KU70**	Human *XRCC6* sequence subcloned into pEGFP-C1 (Clontech)	Gift from Akira Yasui [104]
**MajSat gRNA**	Major satellite guide RNA sequence cloned into pEX-A-U6-gRNA	Gift from Sebastian Butlmann [105]
**mCherry-H1.0, H1.4**	Human histone *H1* variant coding sequence from GFP-tagged construct [106] subcloned into mCherry-H2B [107] replacing H2B	This study
**mCherry-H2B**	Human *HIST1H2BJ* coding sequence cloned into mCherry-C1 plasmid (R. Tsien)	Gift from D. Lleres [107]

Supplementary Table 4: Antibodies

IF: Immunofluorescence; WB: Western-Blot, ChIP: Chromatin immunoprecipitation

Type	Antibody target	Species	Supplier	Dilution/ amount	Application
Primary	**ATRX**	Rabbit	Santa Cruz Biotechnology (sc-15408)	1:400	IF
				1:500	WB
	**CAF-1 p60**	Mouse	Active Motif (39996)	1:500	IF
	**CAF-1 p150**	Goat	Santa Cruz Biotechnology (sc-10206)	1:50	IF
				1:250	WB
	**CPD**	Mouse	Kamiya Biomedical Company (MC-062, clone KTM53)	1:1000	IF
			Cosmo Bio (CAC-NM-DND-001, clone TDM2)	1:1000	IF
	**DAXX**	Rabbit	Santa Cruz Biotechnology (sc-7152)	1:250	IF
			Sigma-Aldrich (HPA008736)	1:100	IF
			Ozyme (4533)	1:100	WB
	**DDB1**	Rabbit	Bethyl laboratories (A300-426A)	1:2000	WB
	**DDB2**	Mouse	Abcam (ab51017)	1:200	WB
	**EZH2**	Mouse	BD-Biosciences (612666)	1:100	IF
	**GFP**	Rat	Santa Cruz Biotechnology (sc-101536)	1:50	IF
		Mouse	Roche Applied Science (11814460001)	1:1000	WB
	**γH2A.X**	Mouse	MERCK Millipore (05-636, clone	1:1000	IF
				1:1000	WB

		JBW301)		
**H3**	Rabbit	Abcam (ab1791)	2 µg	ChIP
			1:5000	WB
**H3.3**	Rabbit	MERCK Millipore (09-838)	1:1000	WB
**H3K4me3**	Rabbit	MERCK Millipore (07-473)	1:5000	IF
**H3K9me3**	Rabbit	Active Motif (39765)	1:500	IF
			1:1000	WB
		Abcam (ab8898)	3 µg	ChIP
			1:1000	WB
**H4K20me3**	Rabbit	Abcam (ab9053)	1:500	IF
**HIRA**	Mouse	Active Motif (39557)	1:100	IF
			1:200	WB
**HP1α**	Mouse	Millipore (MAB3584)	1:500	IF
**Poly(ADP-ribose)**	Rabbit	Trevigen (4336-BPC-100)	1:200	IF
**PCNA**	Rabbit	Santa Cruz Biotechnology (sc-7907)	1:50	IF
	Mouse	Dako (M0879)	1:1000	IF
**SETDB1**	Mouse	Thermo scientific (MA515722)	1:200	IF
			1:1000	WB
	Rabbit	Santa Cruz Biotechnology (sc-66884)	1:200	IF
	Rabbit	Proteintech (11231-1-AP)	1:100	IF
**SNAP**	Rabbit	Pierce Antibodies (CAB4255)	1:500	IF
			1:1000	WB
**SUV39H1**	Rabbit	Cell signaling technology (8729)	1:25	IF
			1:1000	WB

	**Tubulin**	Mouse	Sigma-Aldrich (T9026)	1:10000	WB
	**XPA**	Mouse	BD Biosciences (556453)	1:500	IF
	**XPB**	Rabbit	Santa Cruz Biotechnology (sc-293)	1:400	IF
Secondary	**Goat HRP**	Donkey	Santa Cruz Biotechnology (sc-2020)	1:10000	WB
	**Mouse HRP**	Goat	Jackson Immunoresearch Laboratories (115-035-068)	1:10000	WB
	**Rabbit HRP**	Donkey	Jackson Immunoresearch Laboratories (711-035-152)	1:10000	WB
	**Anti-Rabbit IRDye 680RD Conjugated**	Goat	LI-COR Biosciences (926-68071)	1:15000	WB
	**Anti-Rabbit IRDye 800CW Conjugated**	Goat	LI-COR Biosciences (926-32211)	1:15000	WB
	**Anti-Mouse IRDye 680RD Conjugated**	Goat	LI-COR Biosciences (926-68070)	1:15000	WB
	**Anti-Mouse IRDye 800CW Conjugated**	Goat	LI-COR Biosciences (926-32210)	1:15000	WB
	**Goat Alexa Fluor 594**	Donkey	Invitrogen (A11058)	1:1000	IF
	**Mouse Alexa Fluor 488**	Goat	Invitrogen (A11029)	1:1000	IF
	**Mouse Alexa Fluor 568**	Goat	Invitrogen (A11031)	1:1000	IF
	**Mouse Alexa Fluor 594**	Goat	Invitrogen (A11032)	1:1000	IF
	**Mouse Alexa Fluor 647**	Goat	Invitrogen (A21236)	1:1000	IF

	**Rabbit Alexa Fluor 568**	Goat	Invitrogen (A11036)	1:1000	IF
	**Rabbit Alexa Fluor 594**	Goat	Invitrogen (A11037)	1:1000	IF
	**Rabbit Alexa Fluor 647**	Goat	Invitrogen (A21245)	1:1000	IF
	**Rat Alexa Fluor 488**	Goat	Invitrogen (A11006)	1:1000	IF

Supplementary Table 5: qRT-PCR primers and FISH probes

F: forward; R: reverse.

**Designation**	**Sequence**	**Application**
Major satellite_F	$^{5'}$ GACGACTTGAAAAATGACGAAATC $^{3'}$	RT-qPCR
Major satellite_R	$^{5'}$ CATATTCCAGGTCCTTCAGTGTGC $^{3'}$	RT-qPCR
GAPDH_F	$^{5'}$ TGCACCACCAACTGCTTAGC $^{3'}$	RT-qPCR
GAPDH_R	$^{5'}$ GGCATGGACTGTGGTCATGAG $^{3'}$	RT-qPCR
Major satellite_F	$^{5'}$ ACGTGAAATATGGCGAGGAA $^{3'}$	ChIP
Major satellite_R	$^{5'}$ CAAGTCGTCAAGTGGATGTT $^{3'}$	ChIP
Major satellite_F	$^{5'}$ TCTTGCCATATTCCACGTCC $^{3'}$	DNA-FISH
Major satellite_R	$^{5'}$ GCGAGGAAAACTGAAAAAGG $^{3'}$	DNA-FISH
