# Supplementary material for: Imaging the response to DNA damage in heterochromatin domains reveals core principles of heterochromatin maintenance
Source: Nat Commun. 2021 Apr 23;12:2428. doi: 10.1038/s41467-021-22575-5 (PMC8065061; doi:10.1038/s41467-021-22575-5)
Supplement: Supplementary file 3 — Description of Additional Supplementary Files [file 41467_2021_22575_MOESM3_ESM.pdf]

Description of Additional Supplementary Files

File Name: Supplementary Movie 1

Description: **Pericentric heterochromatin decompaction following UVC laser irradiation (22 min kinetics).**

Heterochromatin decompaction visualized by Hoechst staining during the first 22 min following local damage with the UVC laser in a NIH/3T3 GFP-hDDB2 mouse fibroblast nucleus. 12 images were captured at 2 min intervals and are displayed at 2 frames/sec. The resulting motion picture is shown with a superimposed white arrowhead pointing to the laser irradiation site.

File Name: Supplementary Movie 2

Description: **Pericentric heterochromatin decompaction and recompaction following UVC laser irradiation (12 h kinetics).**

Heterochromatin decompaction and recompaction are visualized by Hoechst staining during the first 12 h following local damage with the UVC laser in a NIH/3T3 GFP-hDDB2 mouse fibroblast nucleus. 24 images were captured at the following time points: before UVC, 8 min, 30 min, 1h45, and every 30 min till 12 h, and are displayed at 2 frames/sec. The resulting motion picture is shown with a superimposed white arrowhead pointing to the laser irradiation site.
