# Supplementary material for: Imaging the response to DNA damage in heterochromatin domains reveals core principles of heterochromatin maintenance
Source: Nat Commun. 2021 Apr 23;12:2428. doi: 10.1038/s41467-021-22575-5 (PMC8065061; doi:10.1038/s41467-021-22575-5)
Supplement: Supplementary file 6 — Reporting Summary [file 41467_2021_22575_MOESM6_ESM.pdf]

nature research

Corresponding author(s): Sophie E. Polo

Last updated by author(s): Feb 15, 2021

Reporting Summary

Nature Research wishes to improve the reproducibility of the work that we publish. This form provides structure for consistency and transparency in reporting. For further information on Nature Research policies, see our Editorial Policies and the Editorial Policy Checklist.

Statistics

For all statistical analyses, confirm that the following items are present in the figure legend, table legend, main text, or Methods section.

n/a	Confirmed	
☐	☒	The exact sample size (*n*) for each experimental group/condition, given as a discrete number and unit of measurement
☐	☒	A statement on whether measurements were taken from distinct samples or whether the same sample was measured repeatedly
☐	☒	The statistical test(s) used AND whether they are one- or two-sided<br>*Only common tests should be described solely by name; describe more complex techniques in the Methods section.*
☒	☐	A description of all covariates tested
☐	☒	A description of any assumptions or corrections, such as tests of normality and adjustment for multiple comparisons
☐	☒	A full description of the statistical parameters including central tendency (e.g. means) or other basic estimates (e.g. regression coefficient) AND variation (e.g. standard deviation) or associated estimates of uncertainty (e.g. confidence intervals)
☐	☒	For null hypothesis testing, the test statistic (e.g. *F*, *t*, *r*) with confidence intervals, effect sizes, degrees of freedom and *P* value noted<br>*Give P values as exact values whenever suitable.*
☒	☐	For Bayesian analysis, information on the choice of priors and Markov chain Monte Carlo settings
☒	☐	For hierarchical and complex designs, identification of the appropriate level for tests and full reporting of outcomes
☒	☐	Estimates of effect sizes (e.g. Cohen's *d*, Pearson's *r*), indicating how they were calculated

Our web collection on statistics for biologists contains articles on many of the points above.

Software and code

Policy information about availability of computer code

Data collection	Metamorph v.7.10.2.240 and Zen Black v.7.1.6.461 and v.14.0.9.201 were used for image acquisition. CellQuest Pro v. 6.0 was used for collecting flow cytometry data.
Data analysis	Image Studio Lite v.5.2.5 was used for western blot quantifications. Image J v.2.0.0-rc-43/1.50i and Imaris v.X64 9.1.2 were used for image analyses. Flow cytometry data were analyzed with FlowJo v.9.9.5. Quantitative PCR analyses were performed with Applied Biosystems 7500 Fast v.2.3. Graphpad Prism (versions 6 to 9) was used for statistical analyses.

For manuscripts utilizing custom algorithms or software that are central to the research but not yet described in published literature, software must be made available to editors and reviewers. We strongly encourage code deposition in a community repository (e.g. GitHub). See the Nature Research guidelines for submitting code & software for further information.

Data

Policy information about availability of data

All manuscripts must include a data availability statement. This statement should provide the following information, where applicable:

- Accession codes, unique identifiers, or web links for publicly available datasets
- A list of figures that have associated raw data
- A description of any restrictions on data availability

All data generated during this study are included in this article and its supplementary information files. Source data for Figures 1-6 and Supplementary figures 1-7 are provided with the paper.

Field-specific reporting

Please select the one below that is the best fit for your research. If you are not sure, read the appropriate sections before making your selection.

☒ Life sciences ☐ Behavioural & social sciences ☐ Ecological, evolutionary & environmental sciences

For a reference copy of the document with all sections, see nature.com/documents/nr-reporting-summary-flat.pdf

Life sciences study design

All studies must disclose on these points even when the disclosure is negative.

Sample size	When analysing fluorescence microscopy images, a minimum sample size of 20-30 cells per condition was chosen because it provided us with sufficient power to discriminate between experimental conditions. For the analysis of micronucleated cells, 500-1000 cells were scored per condition because micronucleus formation is a rare event. For FACS analyses, 20 000 cells were analyzed per sample, consistent with previously published protocols. For all other experiments, sample size was taken as the number of biological replicates.
Data exclusions	No data were excluded from analyses.
Replication	Most experiments have been replicated at least 3 times in this study. All replicates were successful. The number of replicates is indicated in all figure legends.
Randomization	No groups were needed for this study.
Blinding	Experimenters were not blinded to the experimental conditions. This was not necessary because the cells to be imaged were randomly selected based only on the presence of DNA damage on heterochromatin domains and not based on the signal of interest. Furthermore, in most cases, analyses and quantifications of fluorescence microscopy images were automated using Fiji's macros, therefore minimizing the subjectivity of the experimenters.

Reporting for specific materials, systems and methods

We require information from authors about some types of materials, experimental systems and methods used in many studies. Here, indicate whether each material, system or method listed is relevant to your study. If you are not sure if a list item applies to your research, read the appropriate section before selecting a response.

Materials & experimental systems

n/a	Involved in the study	
☐	☒	Antibodies
☐	☒	Eukaryotic cell lines
☒	☐	Palaeontology and archaeology
☒	☐	Animals and other organisms
☒	☐	Human research participants
☒	☐	Clinical data
☒	☐	Dual use research of concern

Methods

n/a	Involved in the study	
☒	☐	ChIP-seq
☐	☒	Flow cytometry
☒	☐	MRI-based neuroimaging

Antibodies

Antibodies used

ATRX, Santa Cruz Biotechnology (sc-15408)
CAF-1 p60, Active Motif (39996)
CAF-1 p150, Santa Cruz Biotechnology (sc-10206)
CPD, Kamiya Biomedical Company (MC-062, clone KTM53)
CPD, Cosmo Bio (CAC-NM-DND-001, clone TDM2)
DAXX, Santa Cruz Biotechnology (sc-7152)
DAXX, Sigma-Aldrich (HPA008736)
DAXX, Ozyme (4533)
DDB1, Bethyl laboratories (A300-426A)
DDB2, Abcam (ab51017)
EZH2, BD-Biosciences (612666)
GFP, Santa Cruz Biotechnology (sc-101536)
GFP, Roche Applied Science (11814460001)
γH2A.X, MERCK Millipore (05-636, clone JBW301)
H3, Abcam (ab1791)
H3.3, MERCK Millipore (09-838)
H3K4me3, MERCK Millipore (07-473)
H3K9me3, Active Motif (39765)

H3K9me3, Abcam (ab8898)
H4K20me3, Abcam (ab9053)
HIRA, Active Motif (39557)
HP1α, Millipore (MAB3584)
PAR=Poly(ADP-ribose), Trevigen (4336-BPC-100)
PCNA, Santa Cruz Biotechnology (sc-7907)
PCNA, Dako (M0879, clone PC-10)
SETDB1, Thermo scientific (MA515722)
SETDB1, Santa Cruz Biotechnology (sc-66884)
SETDB1, Proteintech (11231-1-AP)
SNAP, Pierce Antibodies (CAB4255)
SUV39H1, Cell signaling technology (8729)
Tubulin, Sigma-Aldrich (T9026)
XPA, BD Biosciences (556453)
XPB, Santa Cruz Biotechnology (sc-293)
Goat HRP, Santa Cruz Biotechnology (sc-2020)
Mouse HRP, Jackson Immunoresearch (115-035-068)
Rabbit HRP, Jackson Immunoresearch (711-035-152)
Rabbit IRDye 680RD Conjugated, LI-COR Biosciences (926-68071)
Rabbit IRDye 800CW Conjugated, LI-COR Biosciences (926-32211)
Mouse IRDye 680RD Conjugated, LI-COR Biosciences (926-68070)
Mouse IRDye 800CW Conjugated, LI-COR Biosciences (926-32210)
Goat Alexa Fluor 594, Invitrogen (A11058)
Mouse Alexa Fluor 488, Invitrogen (A11029)
Mouse Alexa Fluor 568, Invitrogen (A11031)
Mouse Alexa Fluor 594, Invitrogen (A11032)
Mouse Alexa Fluor 647, Invitrogen (A21236)
Rabbit Alexa Fluor 568, Invitrogen (A11036)
Rabbit Alexa Fluor 594, Invitrogen (A11037)
Rabbit Alexa Fluor 647, Invitrogen (A21245)
Rat Alexa Fluor 488, Invitrogen (A11006)

Validation

All antibodies were purchased from commercial vendors, who provide validation information on their website. In addition, antibody specificity was validated in our lab by western blot and immunofluorescence in control vs siRNA knockdown conditions.

Data provided in the manuscript validating key antibodies used in this study:
- H3K9me3 and SUV39H1 antibodies are validated by siSUV39H1/2 (IF Fig. 1a, WB Fig. 6c)
- HIRA antibody is validated by siHIRA (WB Fig. 5e, IF and WB Suppl. Fig. 6a, WB Suppl. Fig. 6f)
- DAXX antibody is validated by siDAXX (WB Fig. 5e, IF and WB Suppl. Fig. 6a, WB Suppl. Fig. 6f)
- CAF-1 p150 antibody is validated by siCAF-1 (WB Fig. 6c)
- SETDB1 antibody is validated by siSETDB1 (WB Fig. 6d, 6f, WB Suppl. Fig. 7b, 7d, IF Suppl. Fig. 7e)
- ATRX antibody is validated by siATRX (WB Suppl. Fig. 6c)

Validation/specificity statements on manufacturer's websites for key antibodies used in this study:
- CPD antibodies:
Reacts specifically with thymine dimers produced by UV irradiation in double-or single-stranded DNA. Does not react with (6-4) photo products (https://www.kamiyabiomedical.com/pdf/MC-062.pdf)
Reactivity: CPDs in single-stranded DNA, CPDs formed in every dipyrimidine sequence (TT, TC, CT and CC), CPDs formed in oligonucleotides consisting of more than eight bases (https://www.cosmobiousa.com/products/anti-cpds-mab-clone-tdm-2)
- H3K9me3, Active Motif (39765): specificity validated by dot blot (https://www.activemotif.com/catalog/details/39765/histone-h3-trimethyl-lys9-antibody-pab-1#image_4)
-PCNA clone PC-10: In Western blotting of cell extracts of HeLa cells... the antibody labels a band corresponding to PCNA. Excellent specificity. (https://www.agilent.com/en/product/immunohistochemistry/antibodies-controls/primary-antibodies/proliferating-cell-nuclear-antigen-(concentrate)-76551#specifications)

Eukaryotic cell lines

Policy information about cell lines

Cell line source(s)

U2OS (ATCC HTB-96, human osteosarcoma, female)
MCF7 (ATCC HTB-22, human breast adenocarcinoma, female)
NIH/3T3 cells (ATCC CRL-1658, mouse embryonic fibroblast, male)
NIH/3T3 GFP-DDB2 (stable cell line established in this study)
NIH/3T3 GFP-DDB2 H3.3-SNAP (stable cell line established in this study)
NIH/3T3 H3.3-SNAP (stable cell line established in this study)
U2OS H3.3-SNAP (stable cell line described in Dunleavy et al, Nucleus 2011, Ref. 102)

Authentication

The stable cell lines NIH/3T3 GFP-DDB2, NIH/3T3 GFP-DDB2 H3.3-SNAP and NIH/3T3 H3.3-SNAP were authenticated in this study (Suppl. Fig. 1e-f).

Mycoplasma contamination

All cell lines tested negative for mycoplasma contamination.

Commonly misidentified lines
(See ICLAC register)

This study did not involve misidentified cell lines.

Flow Cytometry

Plots

Confirm that:

☒ The axis labels state the marker and fluorochrome used (e.g. CD4-FITC).

☒ The axis scales are clearly visible. Include numbers along axes only for bottom left plot of group (a 'group' is an analysis of identical markers).

☒ All plots are contour plots with outliers or pseudocolor plots.

☒ A numerical value for number of cells or percentage (with statistics) is provided.

Methodology

Sample preparation	Cells were fixed in ice-cold 70% ethanol before DNA staining with 50 µg/ml propidium iodide (Sigma-Aldrich) in PBS containing 0.05% Tween and 0.5 mg/ml RNase A (USB/Affymetrix).
Instrument	BD FACScalibur flow cytometer (BD Biosciences)
Software	FlowJo software (TreeStar)
Cell population abundance	We did not perform cell sorting.
Gating strategy	Cell debris were gated out using FSC/SSC and cell doublets with FL3-A/FL3-H (as shown in Suppl. Fig. 1d)

☒ Tick this box to confirm that a figure exemplifying the gating strategy is provided in the Supplementary Information.

April 2020
