# Supplementary material for: Imaging the response to DNA damage in heterochromatin domains reveals core principles of heterochromatin maintenance
Source: Nat Commun. 2021 Apr 23;12:2428. doi: 10.1038/s41467-021-22575-5 (PMC8065061; doi:10.1038/s41467-021-22575-5)
Supplement: Supplementary file 7 — Source Data [file 41467_2021_22575_MOESM7_ESM.zip › Raw data/Supplementary Figures/Supplementary Figure 1/Suppl Fig 1d/FACS cell lines.pdf]

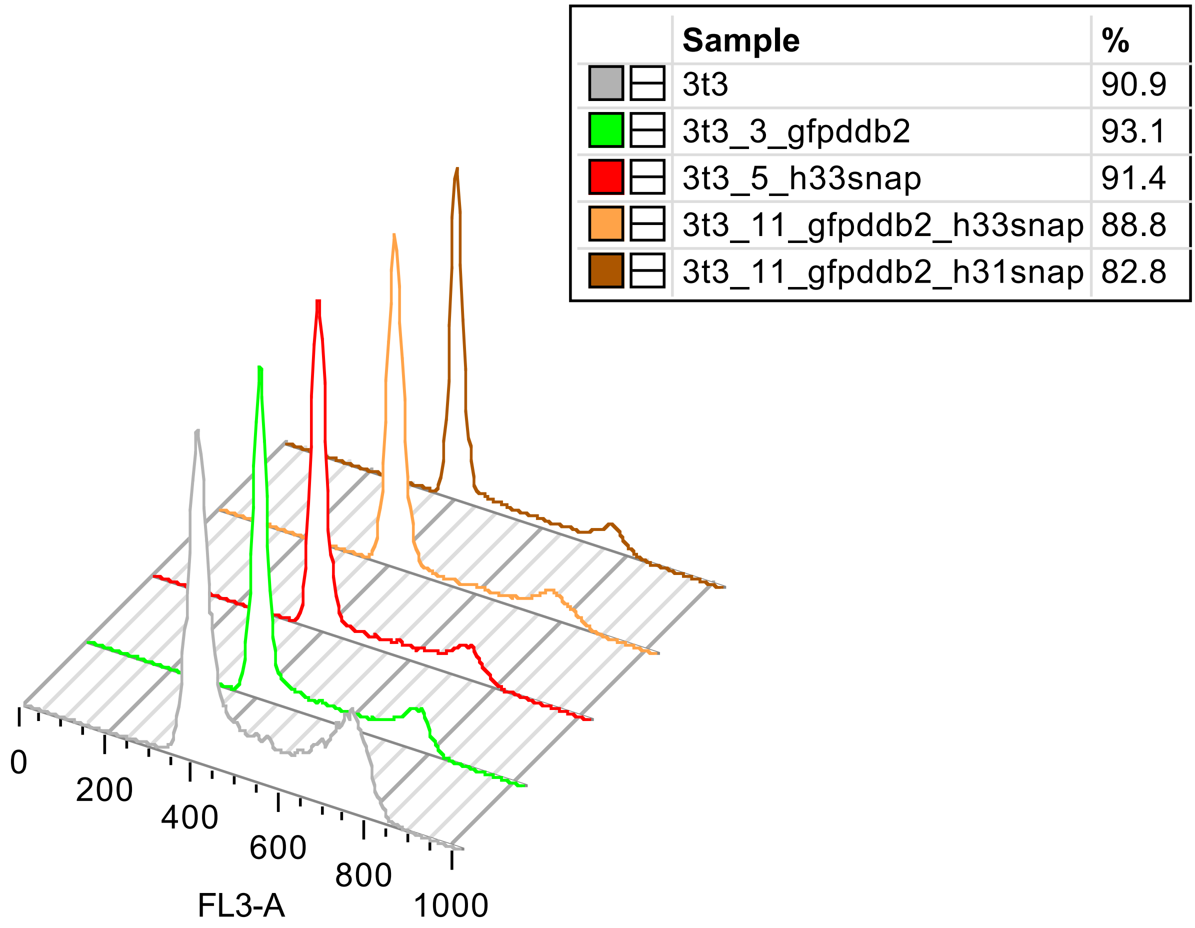

cell selection

Ancestry / Subset / Value Type / For	cel… %G1 W…	ce… %S W…	cel… %G2 W…	cel… %G1 De…	c… %S D…	cel… %G2 De…
3t3	39.4	39.7	19.5	42	28.9	28.5
3t3_3_gfpddb2	57.2	31.6	10.6	57.6	29.6	12.1
3t3_5_h33snap	65	24.6	10.2	65.2	21.4	12.9
3t3_11_gfpddb2_h33snap	71	16	12.1	72.7	14.5	12.4
3t3_11_gfpddb2_h31snap	66.3	24.2	7.69	67.9	23	7.97
