# Supplementary figures and images for: Imaging the response to DNA damage in heterochromatin domains reveals core principles of heterochromatin maintenance
Source: Nat Commun. 2021 Apr 23;12:2428. doi: 10.1038/s41467-021-22575-5 (PMC8065061; doi:10.1038/s41467-021-22575-5)

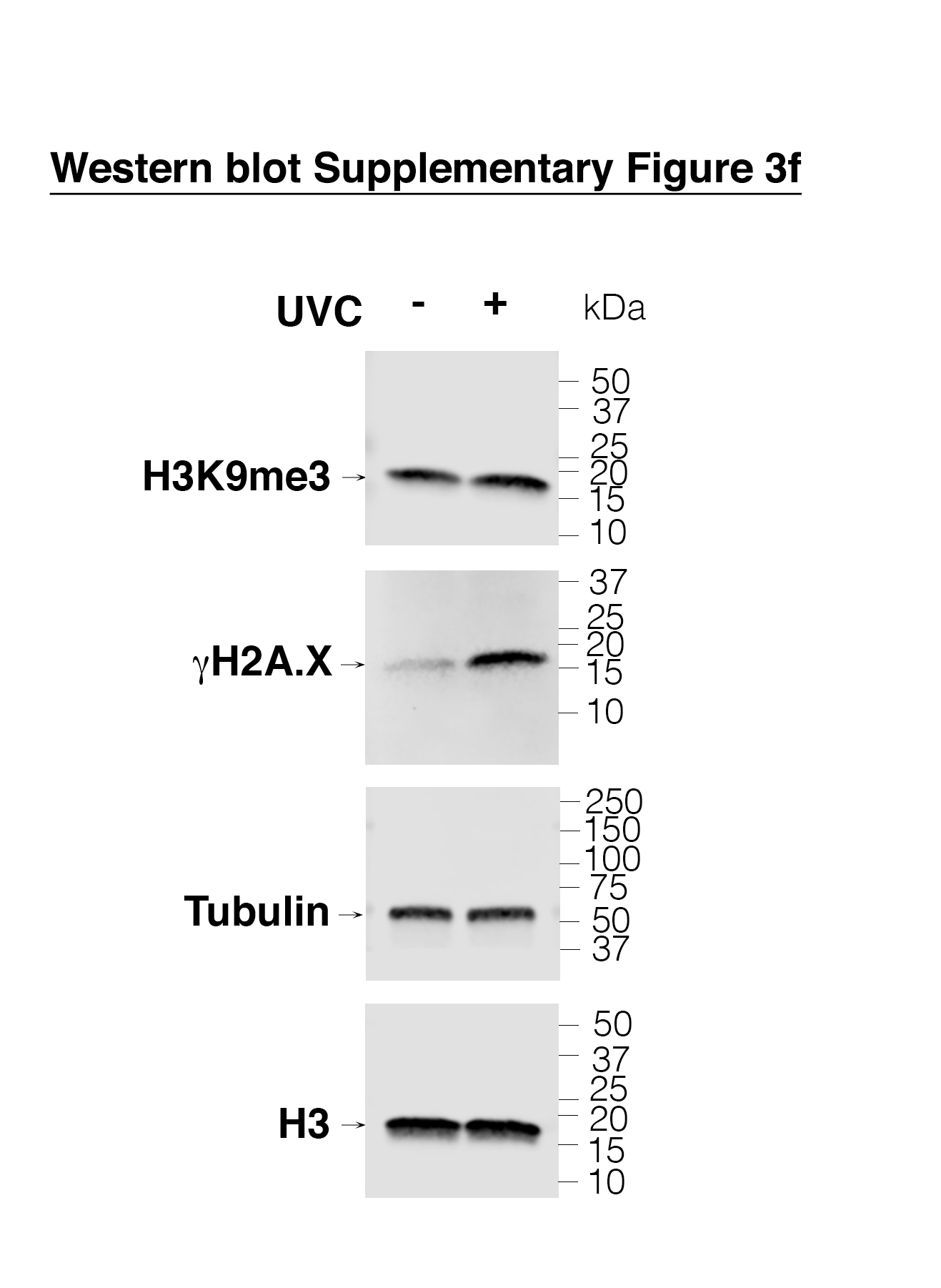

Supplement: Supplementary file 7 — Source Data [file 41467_2021_22575_MOESM7_ESM.zip › Raw data/Supplementary Figures/Supplementary Figure 3/Suppl Fig 3f/Western blot H3K9me3.tif]

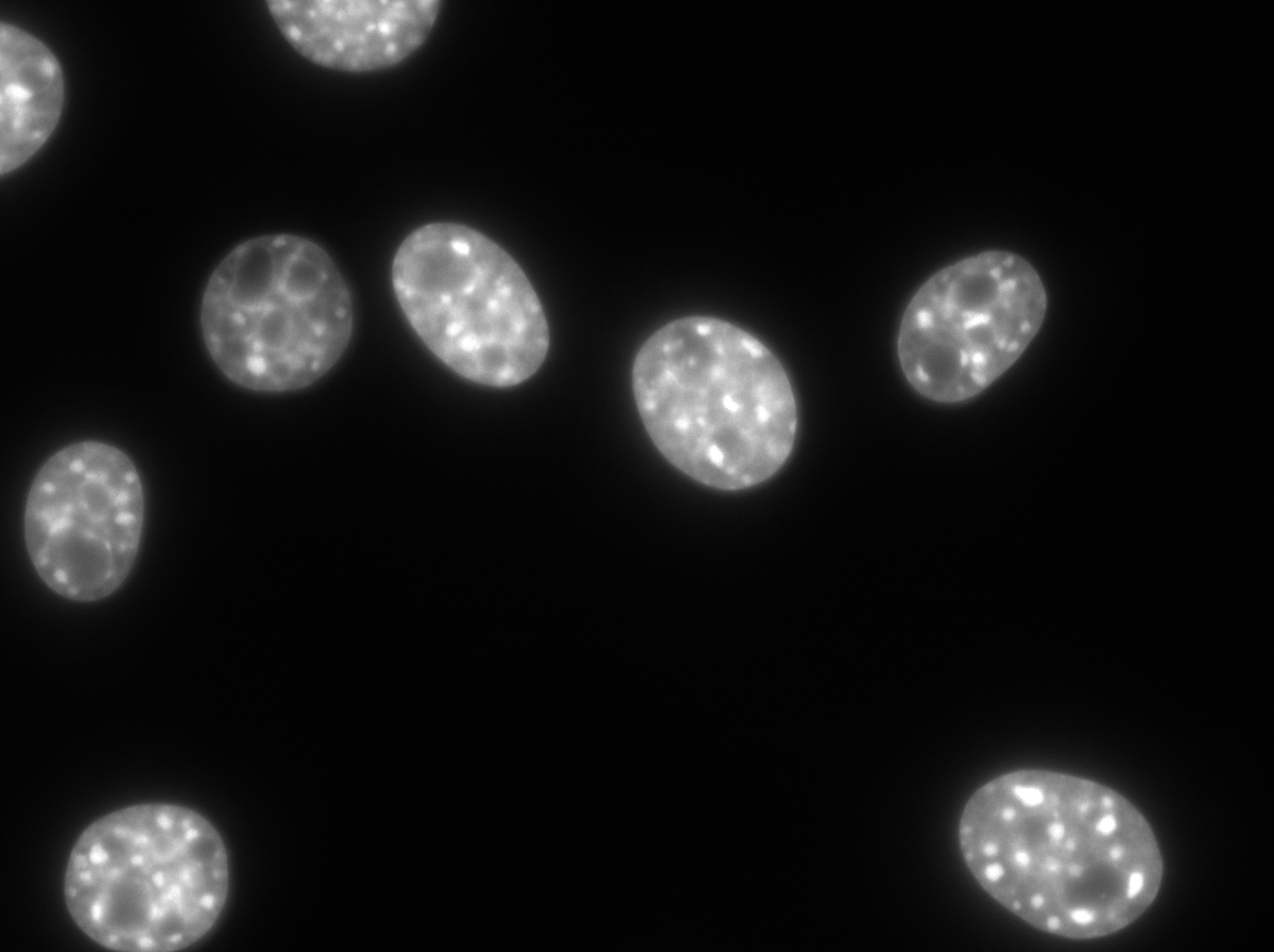

Supplement: Supplementary file 7 — Source Data [file 41467_2021_22575_MOESM7_ESM.zip › Raw data/Supplementary Figures/Supplementary Figure 1/Suppl Fig 1e/3T3_GFPDDB2_w1DAPI.TIF]

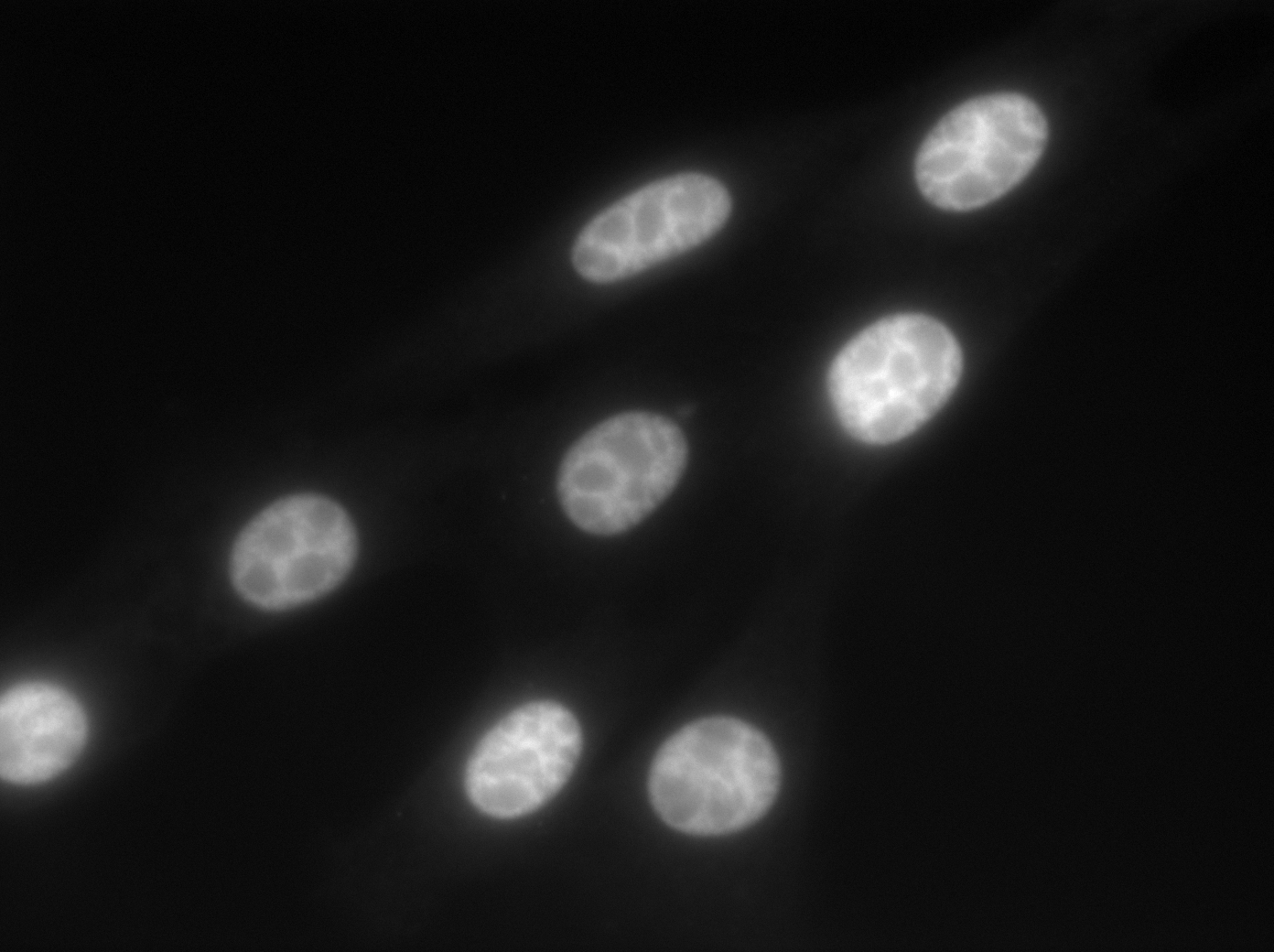

Supplement: Supplementary file 7 — Source Data [file 41467_2021_22575_MOESM7_ESM.zip › Raw data/Supplementary Figures/Supplementary Figure 1/Suppl Fig 1e/3T3_GFPDDB2_H33-SNAP_w2GFP.TIF]

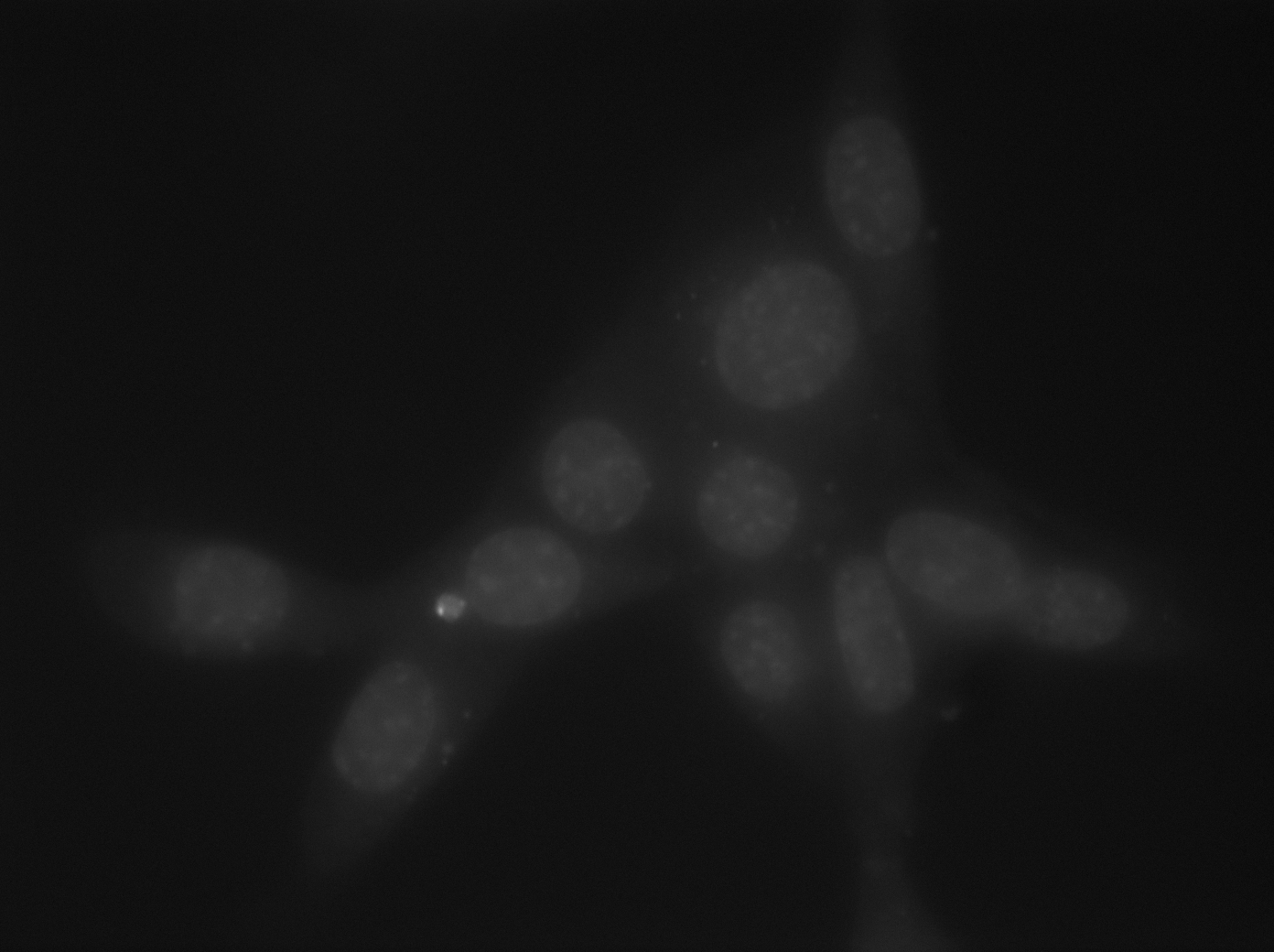

Supplement: Supplementary file 7 — Source Data [file 41467_2021_22575_MOESM7_ESM.zip › Raw data/Supplementary Figures/Supplementary Figure 1/Suppl Fig 1e/3T3_w2GFP.TIF]

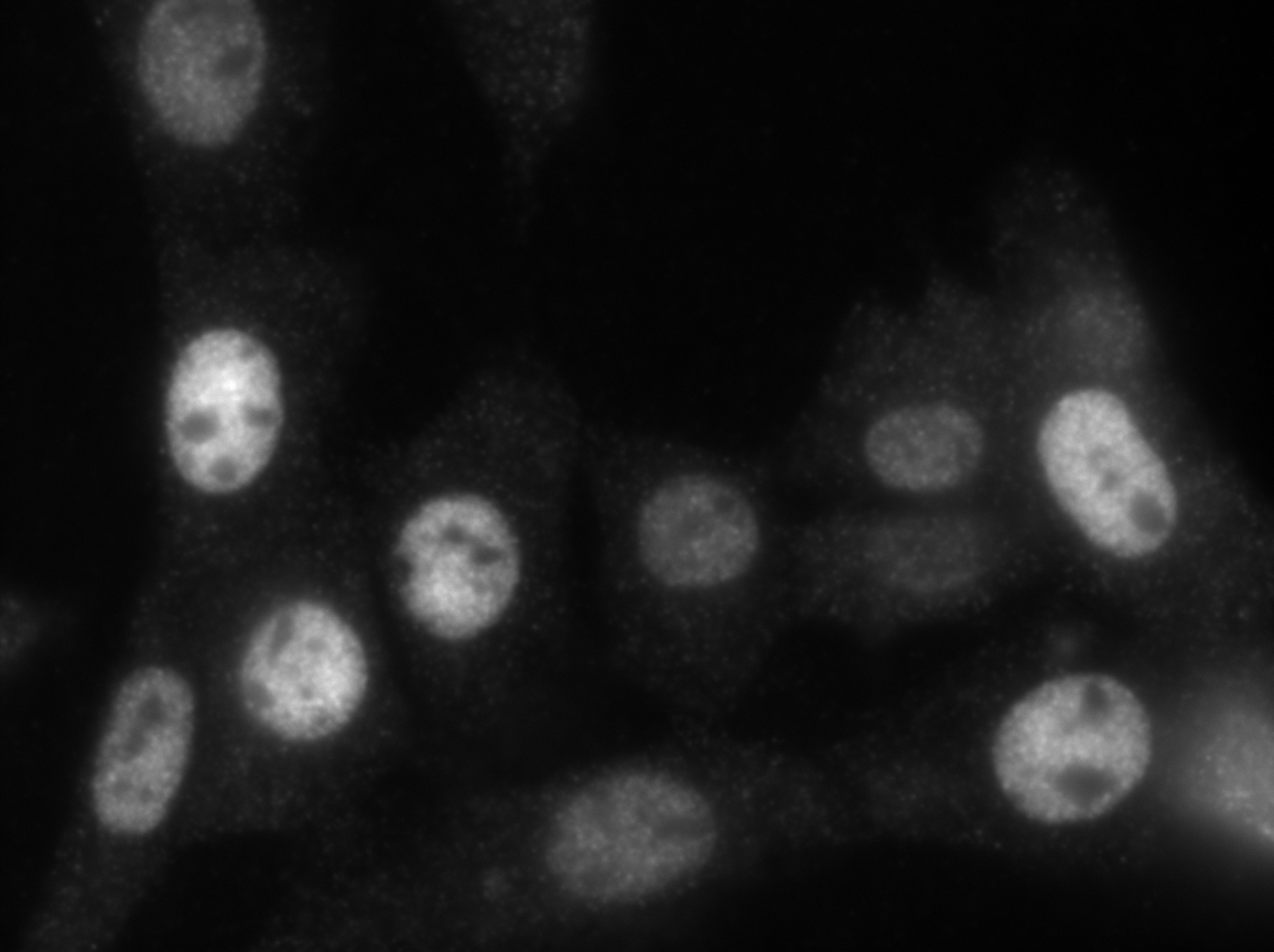

Supplement: Supplementary file 7 — Source Data [file 41467_2021_22575_MOESM7_ESM.zip › Raw data/Supplementary Figures/Supplementary Figure 1/Suppl Fig 1e/3T3_H33-SNAP_w3TX.TIF]

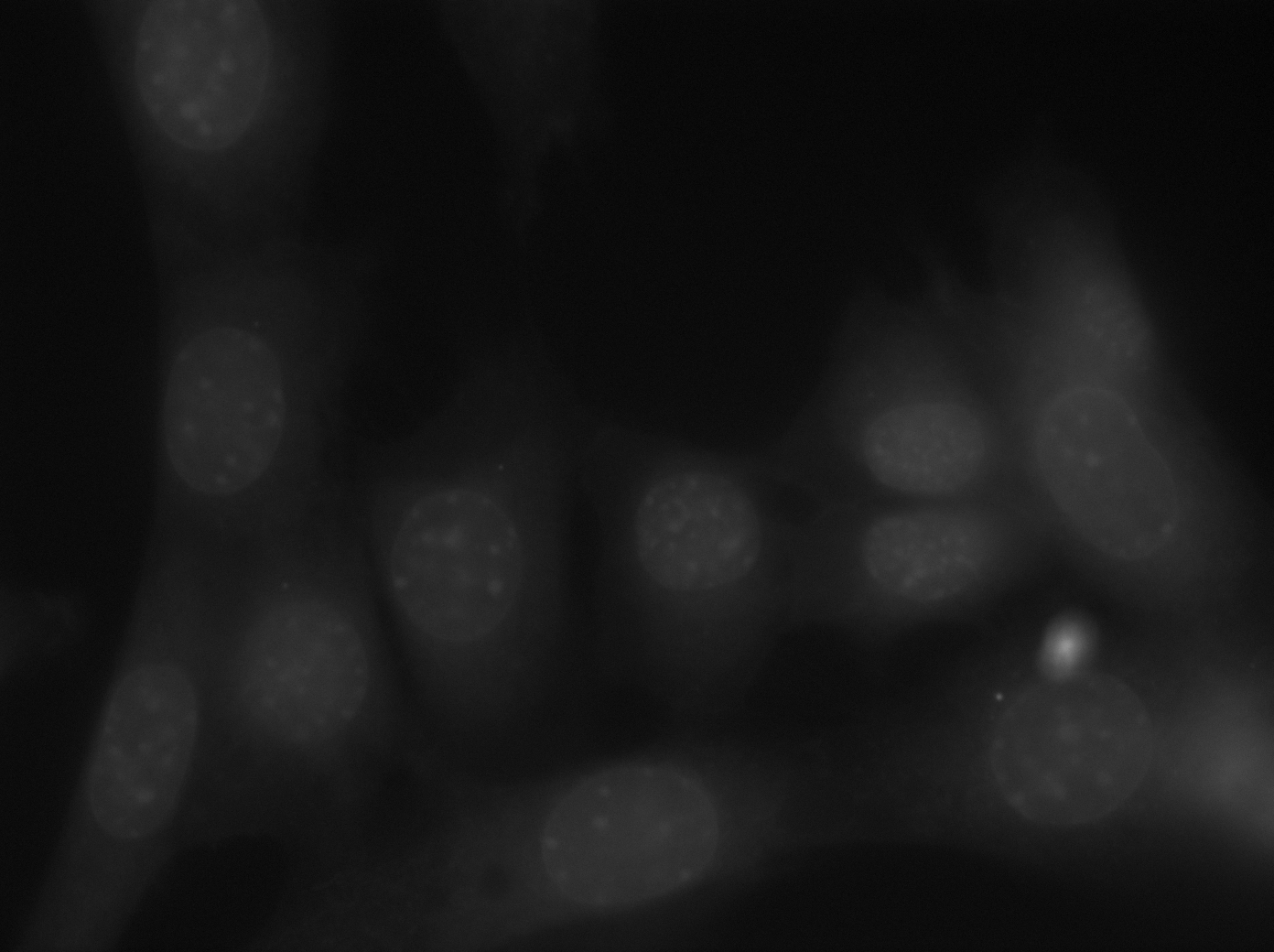

Supplement: Supplementary file 7 — Source Data [file 41467_2021_22575_MOESM7_ESM.zip › Raw data/Supplementary Figures/Supplementary Figure 1/Suppl Fig 1e/3T3_H33-SNAP_w2GFP.TIF]

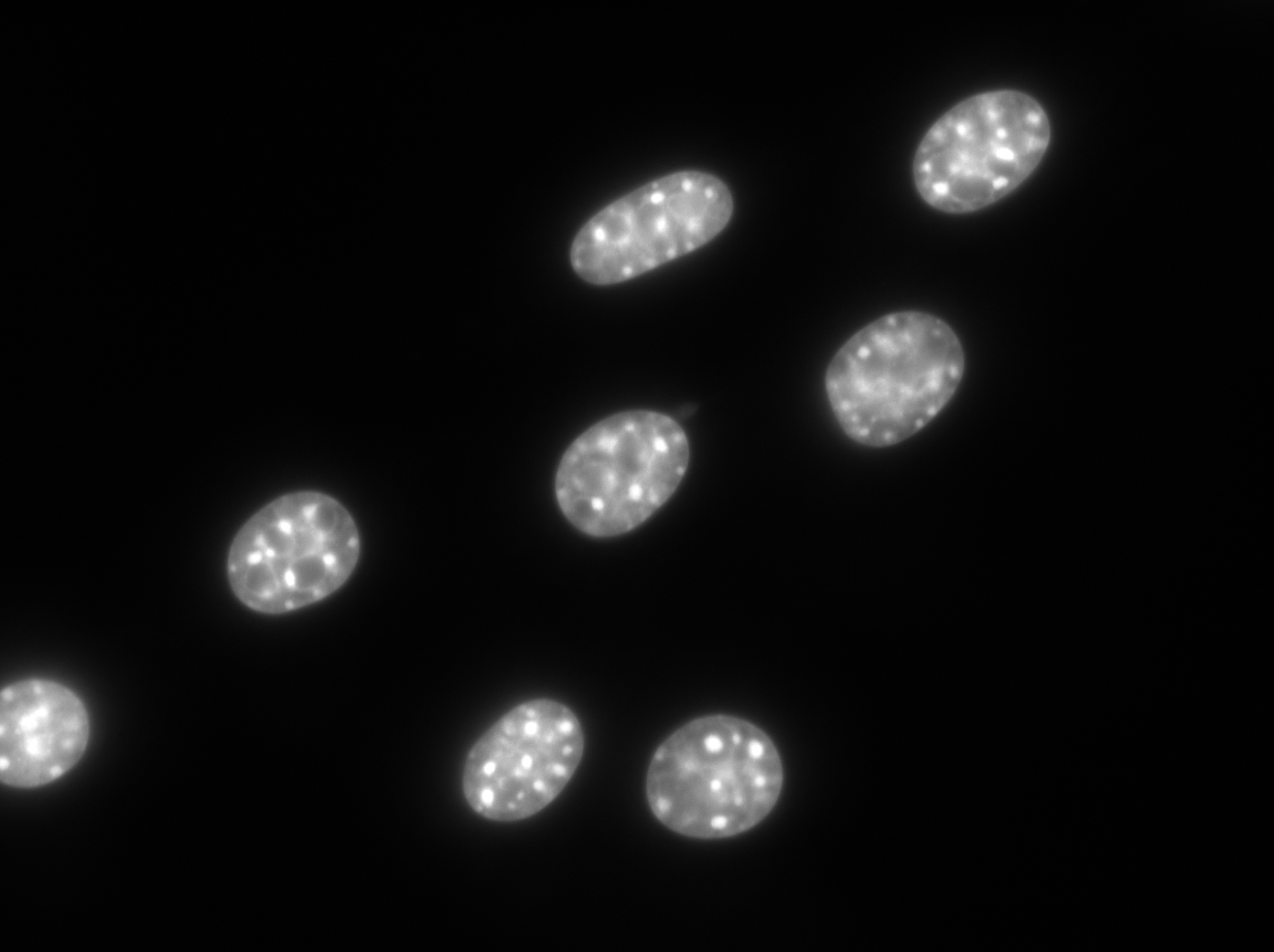

Supplement: Supplementary file 7 — Source Data [file 41467_2021_22575_MOESM7_ESM.zip › Raw data/Supplementary Figures/Supplementary Figure 1/Suppl Fig 1e/3T3_GFPDDB2_H33-SNAP_w1DAPI.TIF]

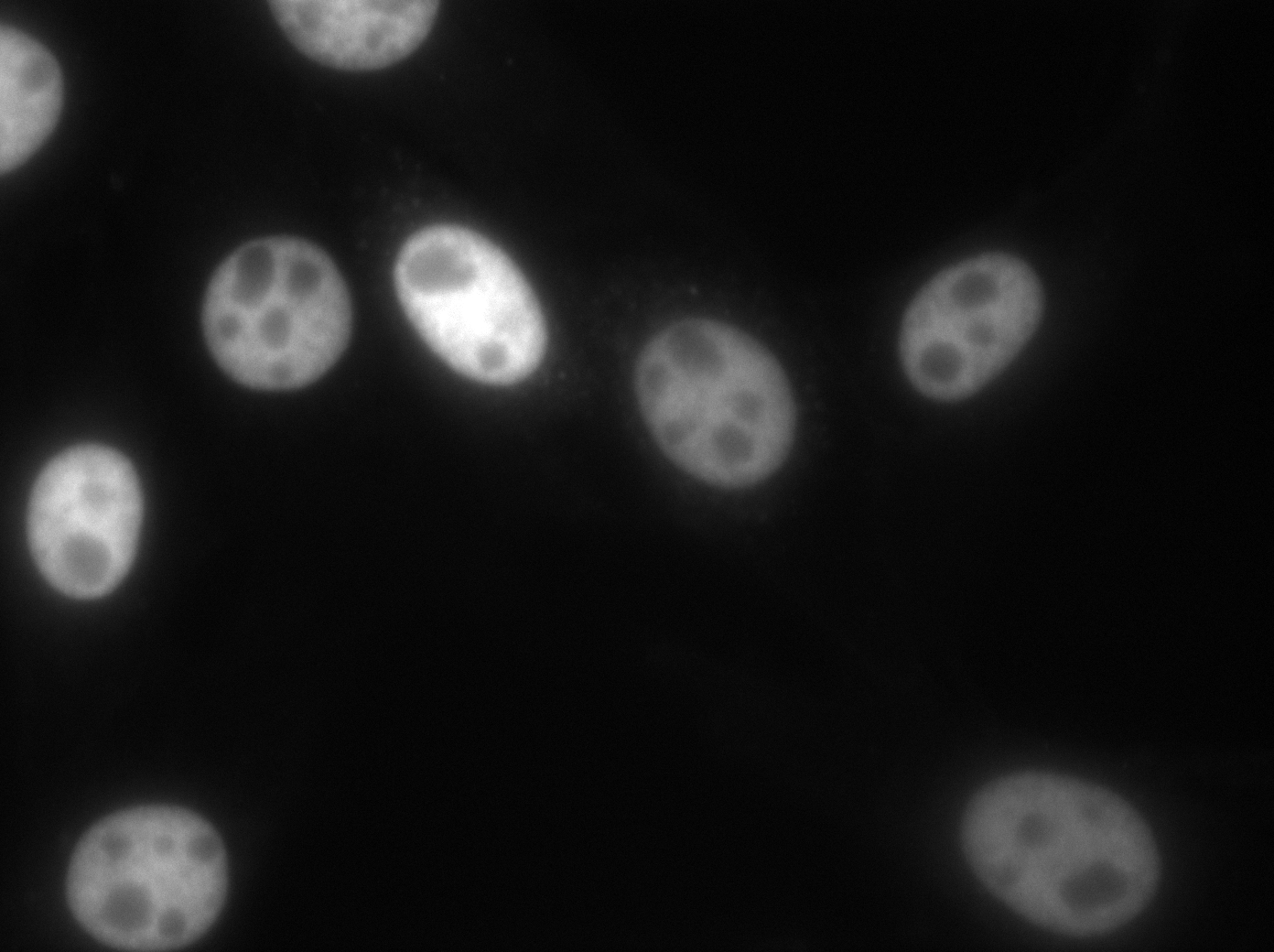

Supplement: Supplementary file 7 — Source Data [file 41467_2021_22575_MOESM7_ESM.zip › Raw data/Supplementary Figures/Supplementary Figure 1/Suppl Fig 1e/3T3_GFPDDB2_w2GFP.TIF]

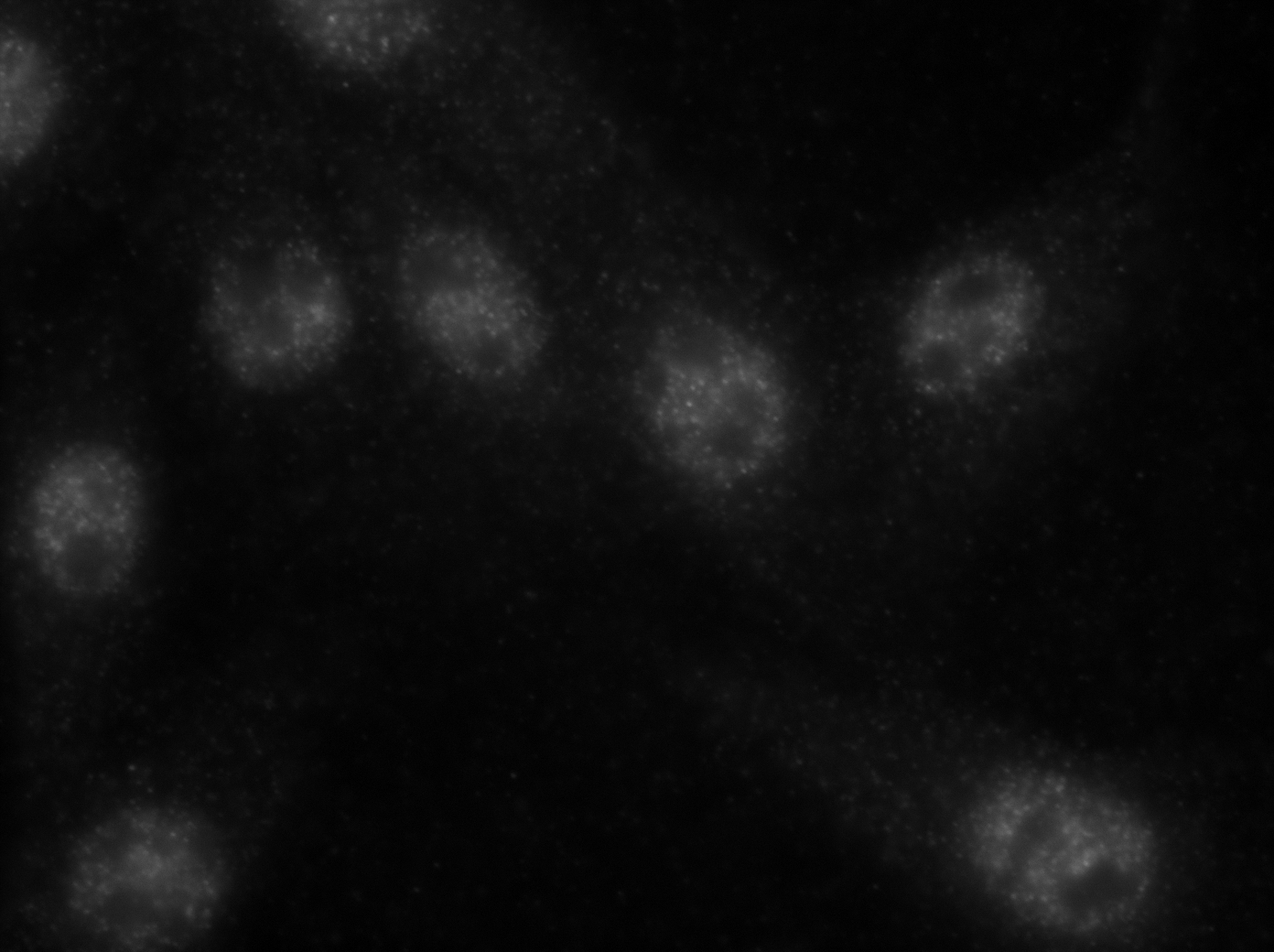

Supplement: Supplementary file 7 — Source Data [file 41467_2021_22575_MOESM7_ESM.zip › Raw data/Supplementary Figures/Supplementary Figure 1/Suppl Fig 1e/3T3_GFPDDB2_w3TX.TIF]

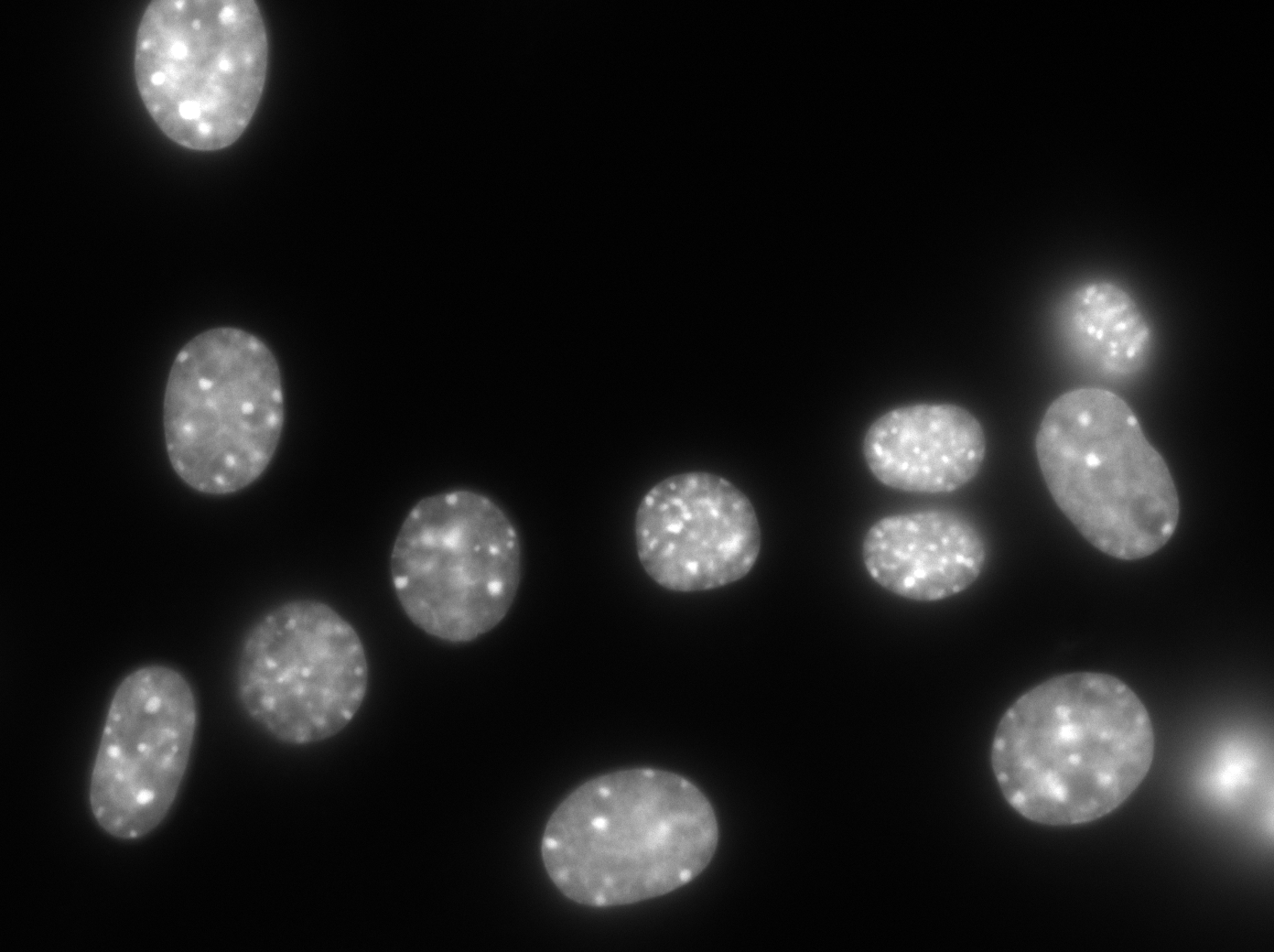

Supplement: Supplementary file 7 — Source Data [file 41467_2021_22575_MOESM7_ESM.zip › Raw data/Supplementary Figures/Supplementary Figure 1/Suppl Fig 1e/3T3_H33-SNAP_w1DAPI.TIF]

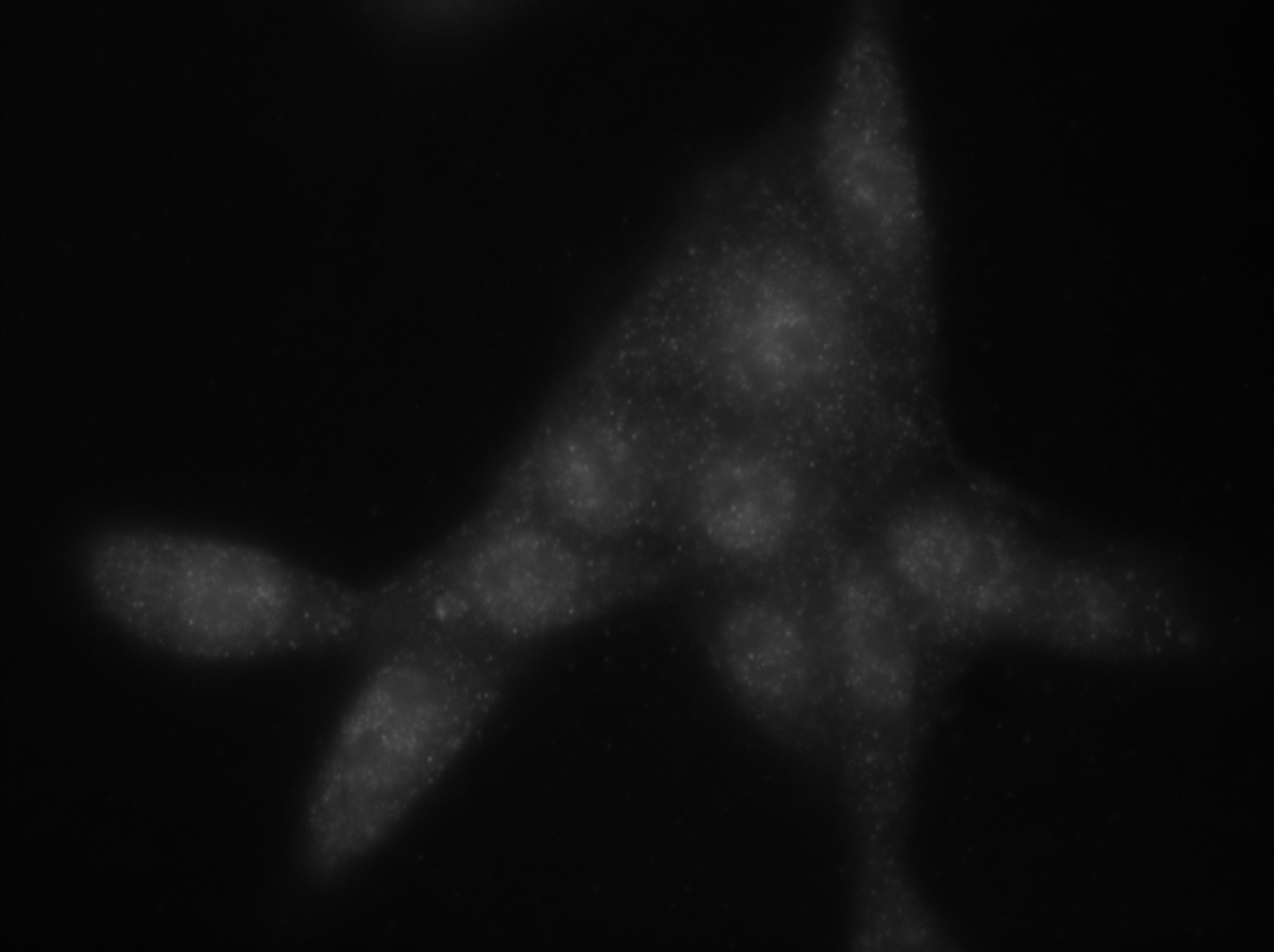

Supplement: Supplementary file 7 — Source Data [file 41467_2021_22575_MOESM7_ESM.zip › Raw data/Supplementary Figures/Supplementary Figure 1/Suppl Fig 1e/3T3_w3TX.TIF]

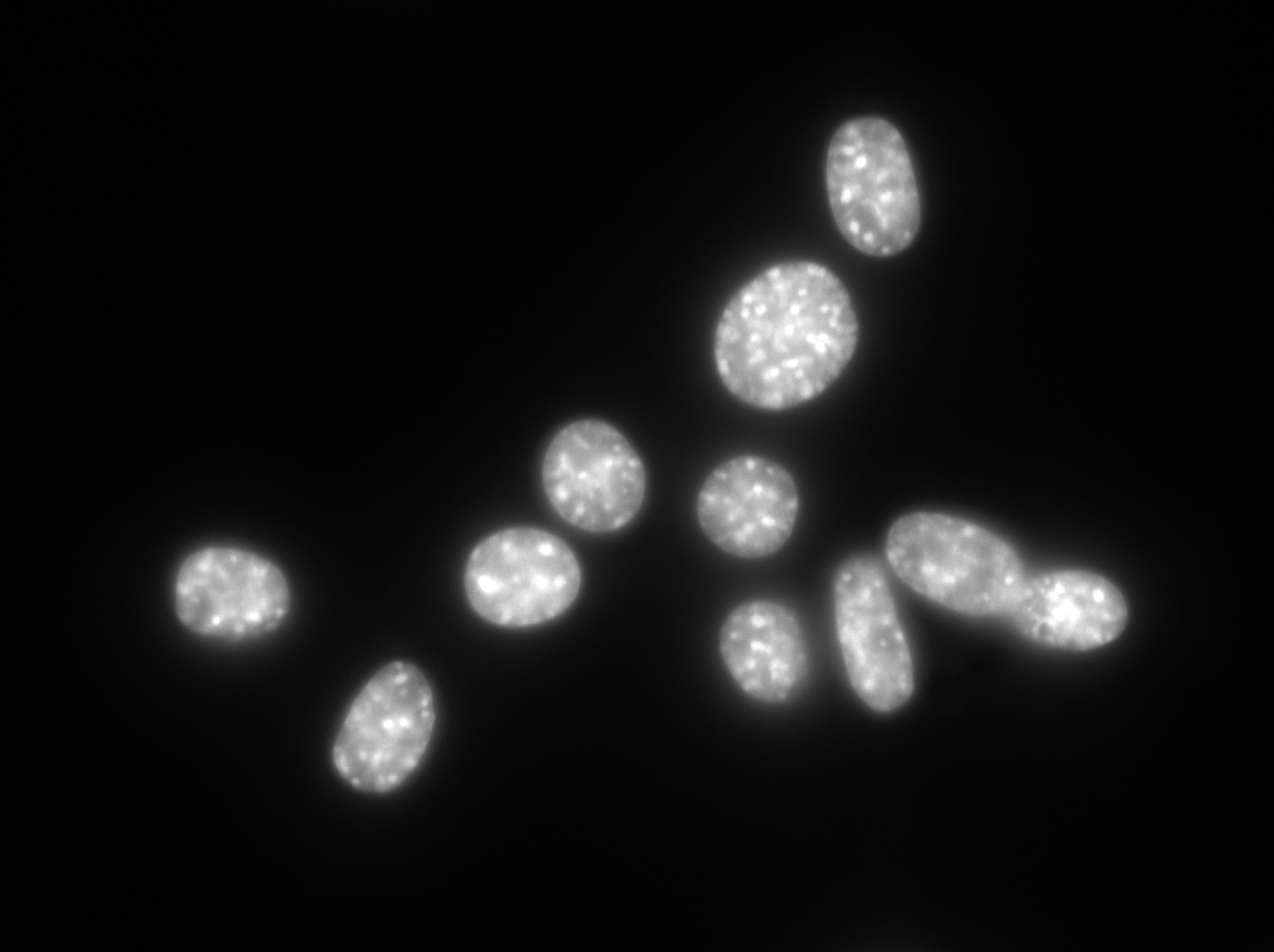

Supplement: Supplementary file 7 — Source Data [file 41467_2021_22575_MOESM7_ESM.zip › Raw data/Supplementary Figures/Supplementary Figure 1/Suppl Fig 1e/3T3_w1DAPI.TIF]

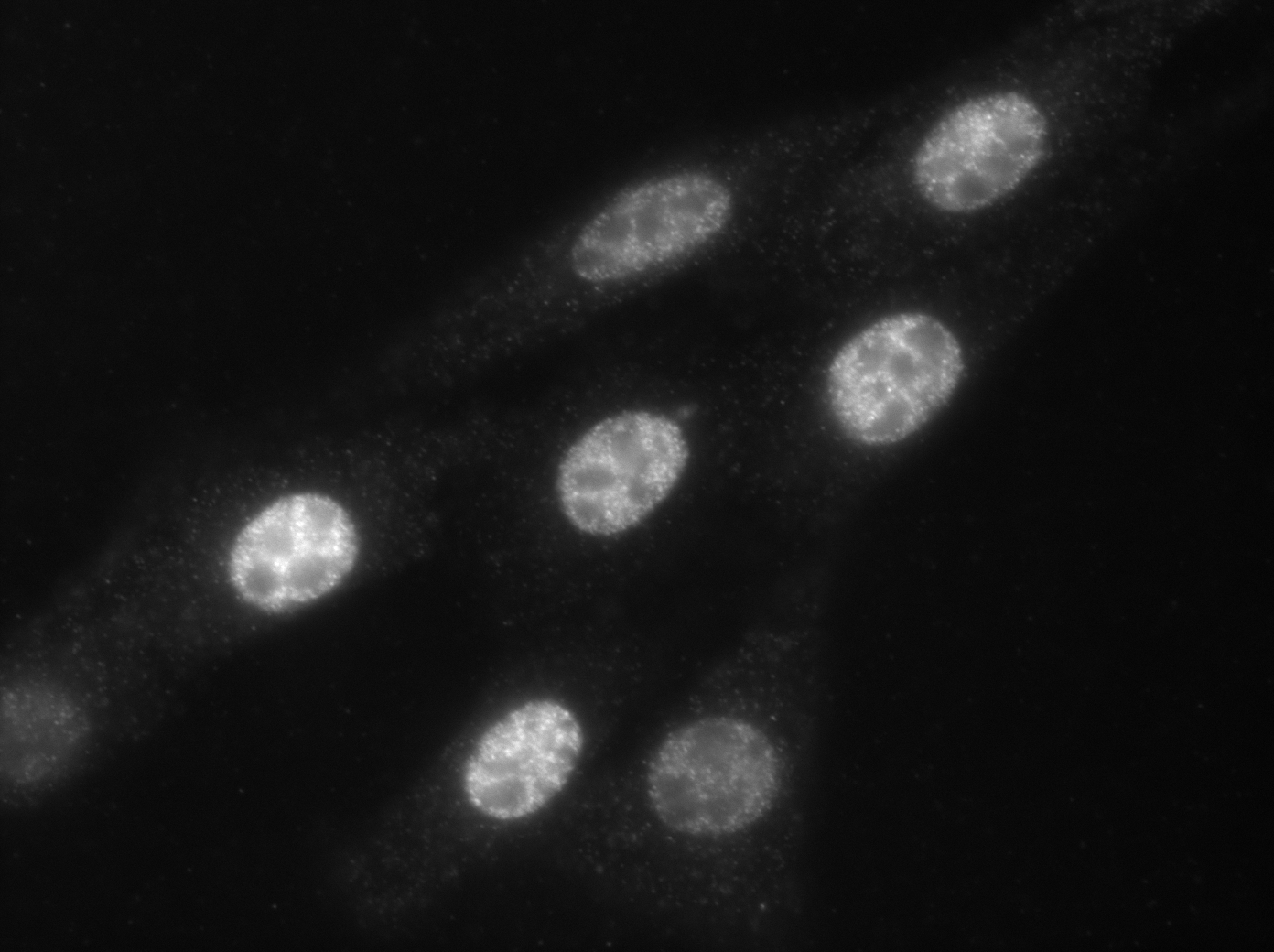

Supplement: Supplementary file 7 — Source Data [file 41467_2021_22575_MOESM7_ESM.zip › Raw data/Supplementary Figures/Supplementary Figure 1/Suppl Fig 1e/3T3_GFPDDB2_H33-SNAP_w3TX.TIF]

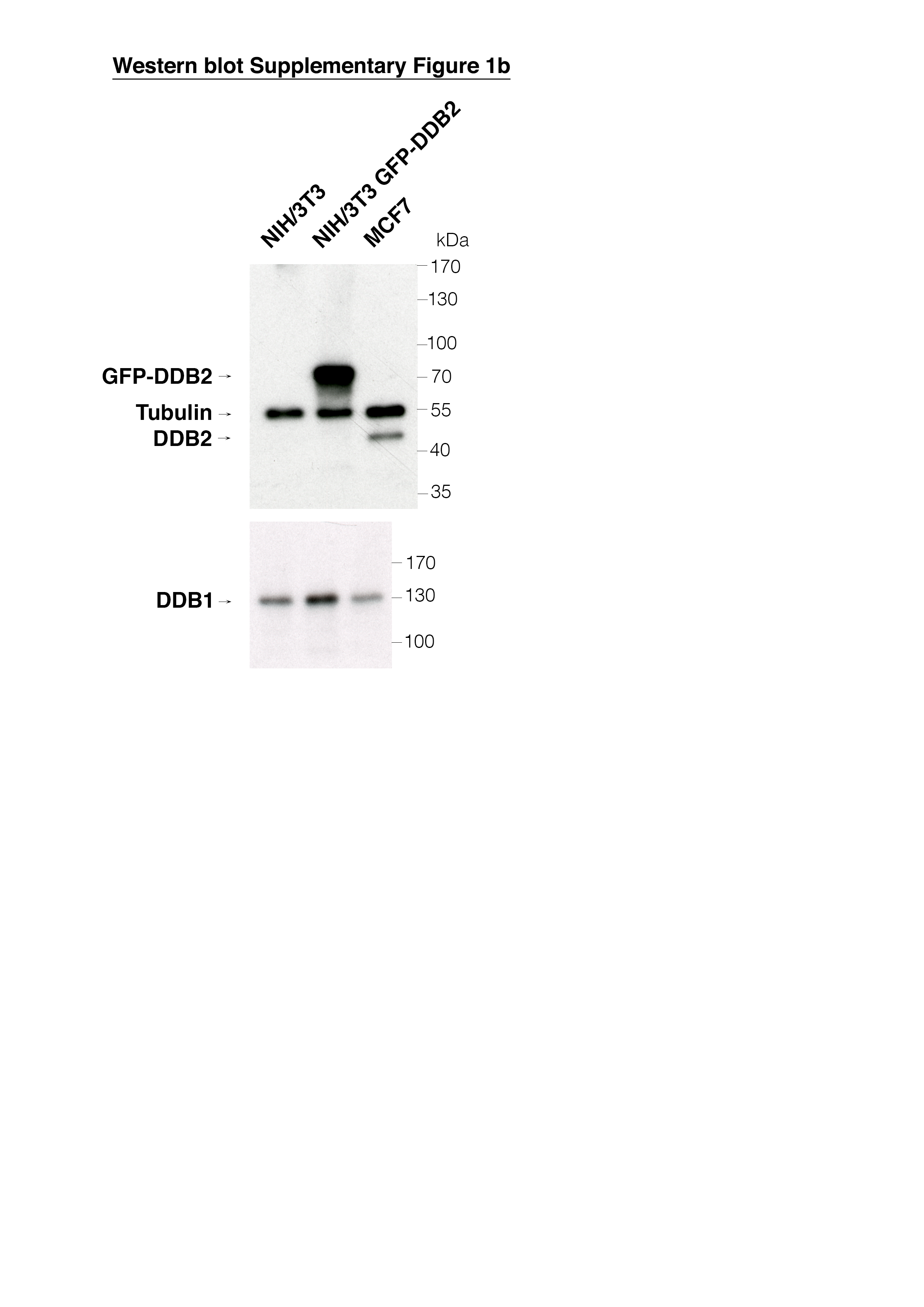

Supplement: Supplementary file 7 — Source Data [file 41467_2021_22575_MOESM7_ESM.zip › Raw data/Supplementary Figures/Supplementary Figure 1/Suppl Fig 1b/Western blot DDB1-DDB2 levels.tif]

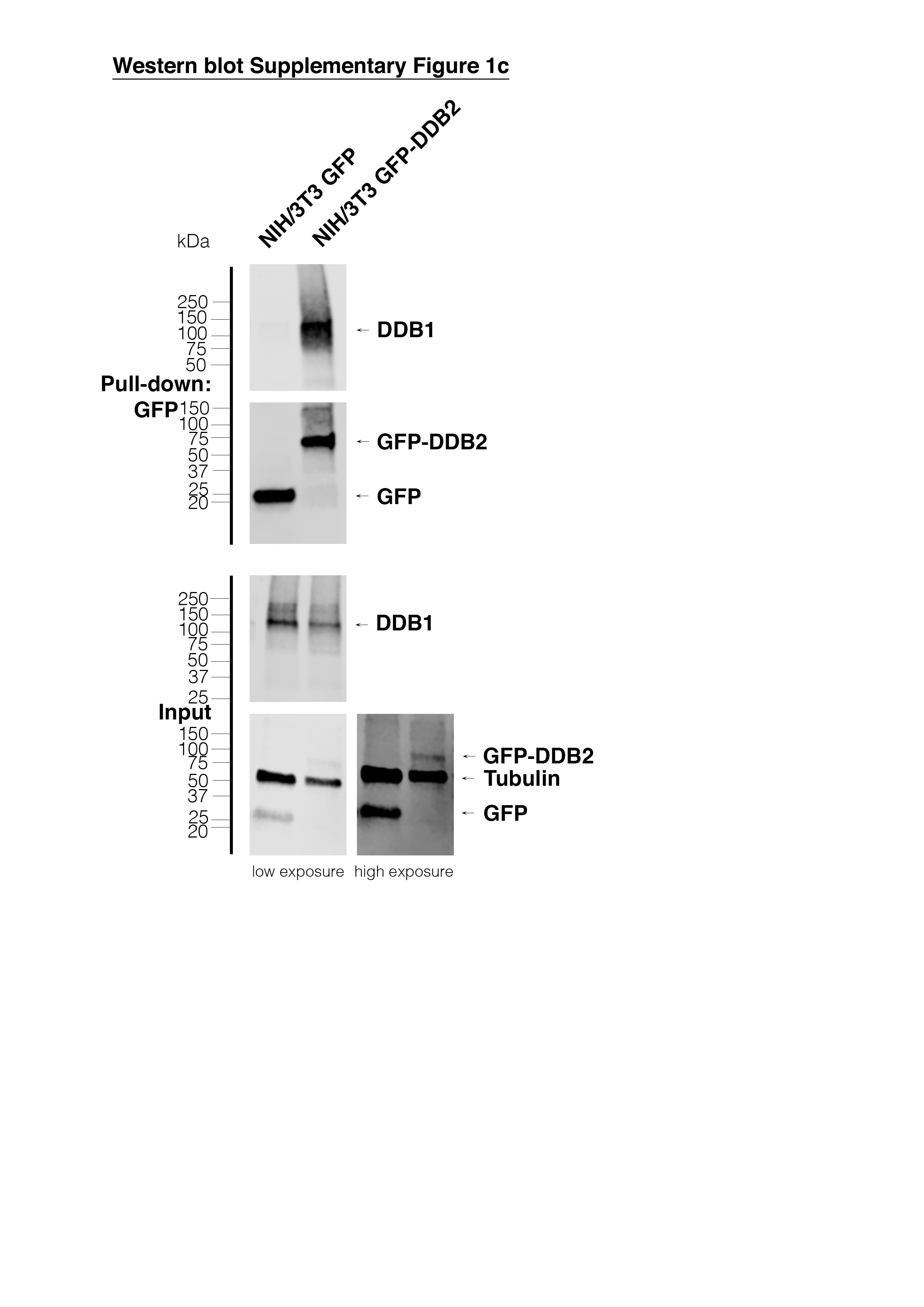

Supplement: Supplementary file 7 — Source Data [file 41467_2021_22575_MOESM7_ESM.zip › Raw data/Supplementary Figures/Supplementary Figure 1/Suppl Fig 1c/Western blot pulldown.tif]

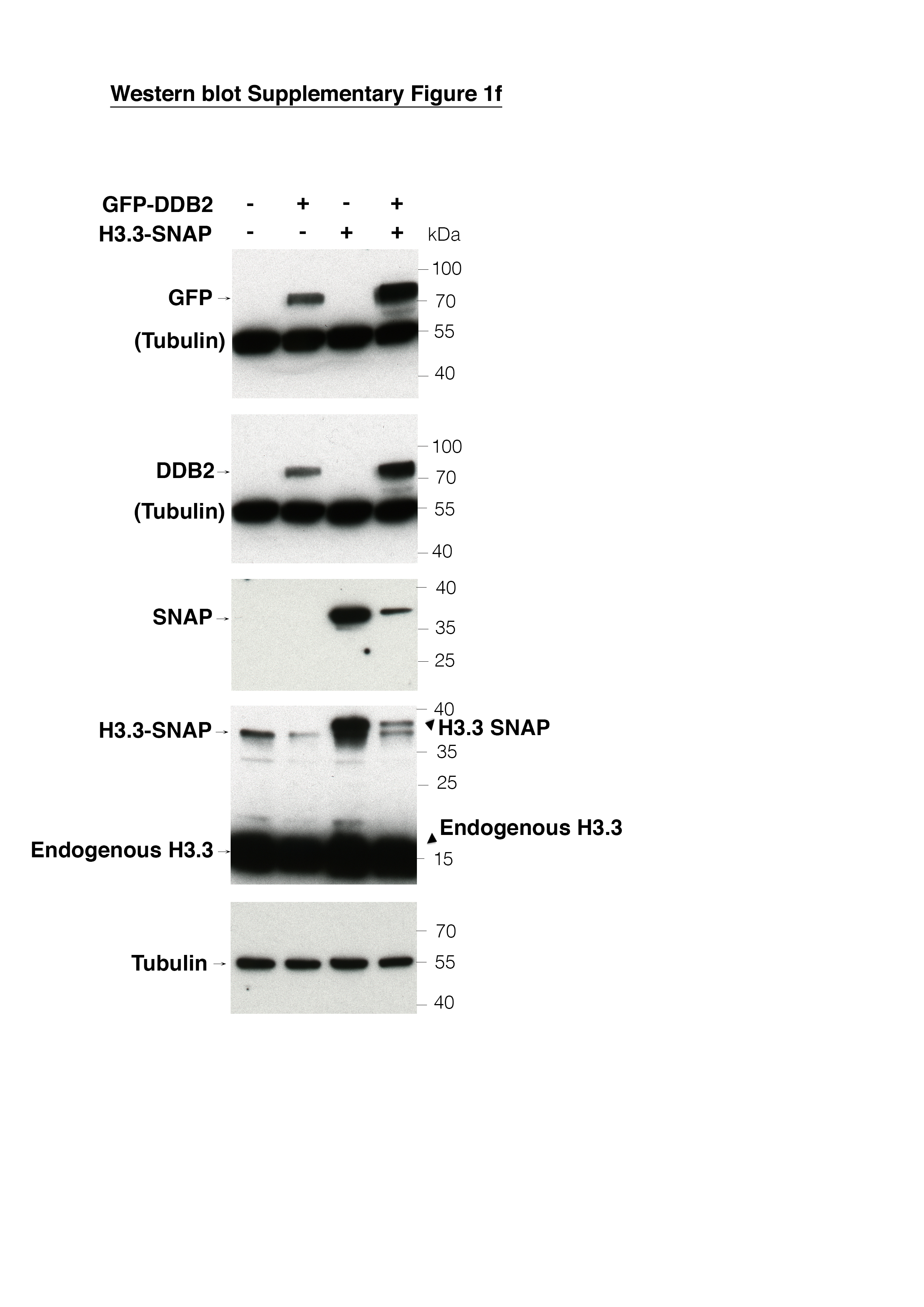

Supplement: Supplementary file 7 — Source Data [file 41467_2021_22575_MOESM7_ESM.zip › Raw data/Supplementary Figures/Supplementary Figure 1/Suppl Fig 1f/Western blot cell lines.tif]

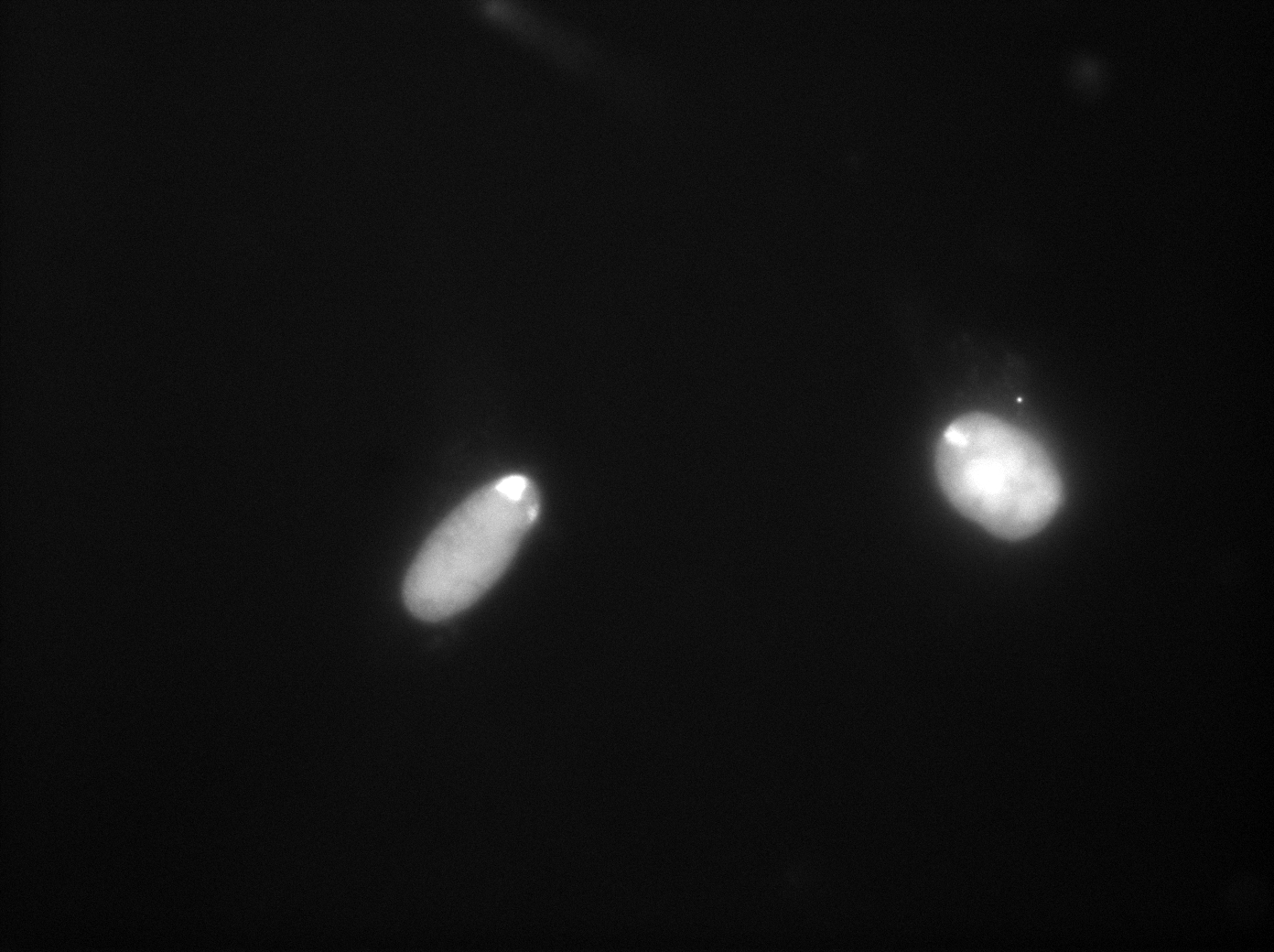

Supplement: Supplementary file 7 — Source Data [file 41467_2021_22575_MOESM7_ESM.zip › Raw data/Supplementary Figures/Supplementary Figure 1/Suppl Fig 1g/images IF/3T3GFPDDB2H33SNAP_newH33(CY3)_CPD(CY5)_w3CY3.TIF]

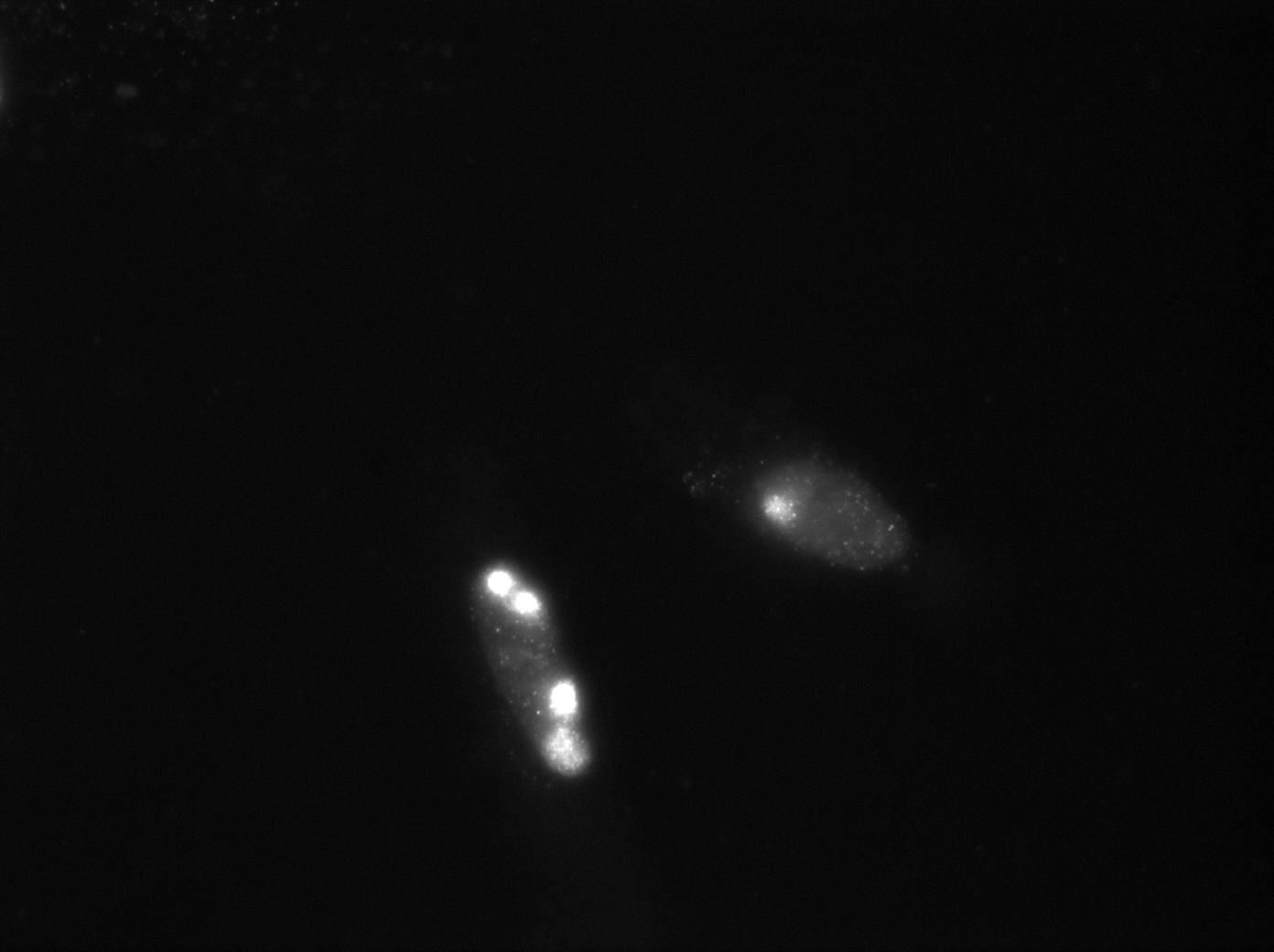

Supplement: Supplementary file 7 — Source Data [file 41467_2021_22575_MOESM7_ESM.zip › Raw data/Supplementary Figures/Supplementary Figure 1/Suppl Fig 1g/images IF/U2OSH33SNAP_newH33(CY3)_CPD(CY5)_w4CY5.TIF]

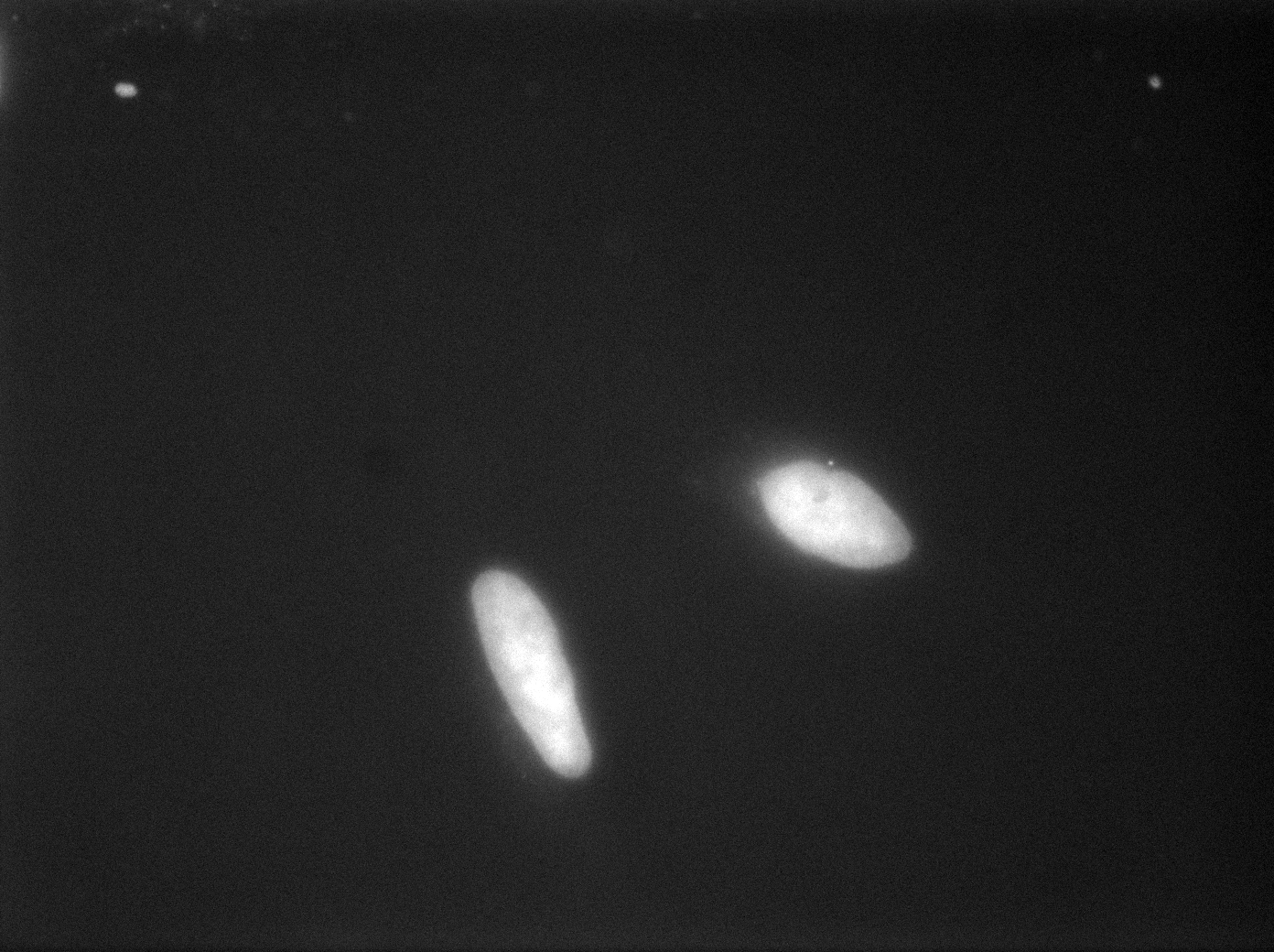

Supplement: Supplementary file 7 — Source Data [file 41467_2021_22575_MOESM7_ESM.zip › Raw data/Supplementary Figures/Supplementary Figure 1/Suppl Fig 1g/images IF/U2OSH33SNAP_newH33(CY3)_CPD(CY5)_w2GFP.TIF]

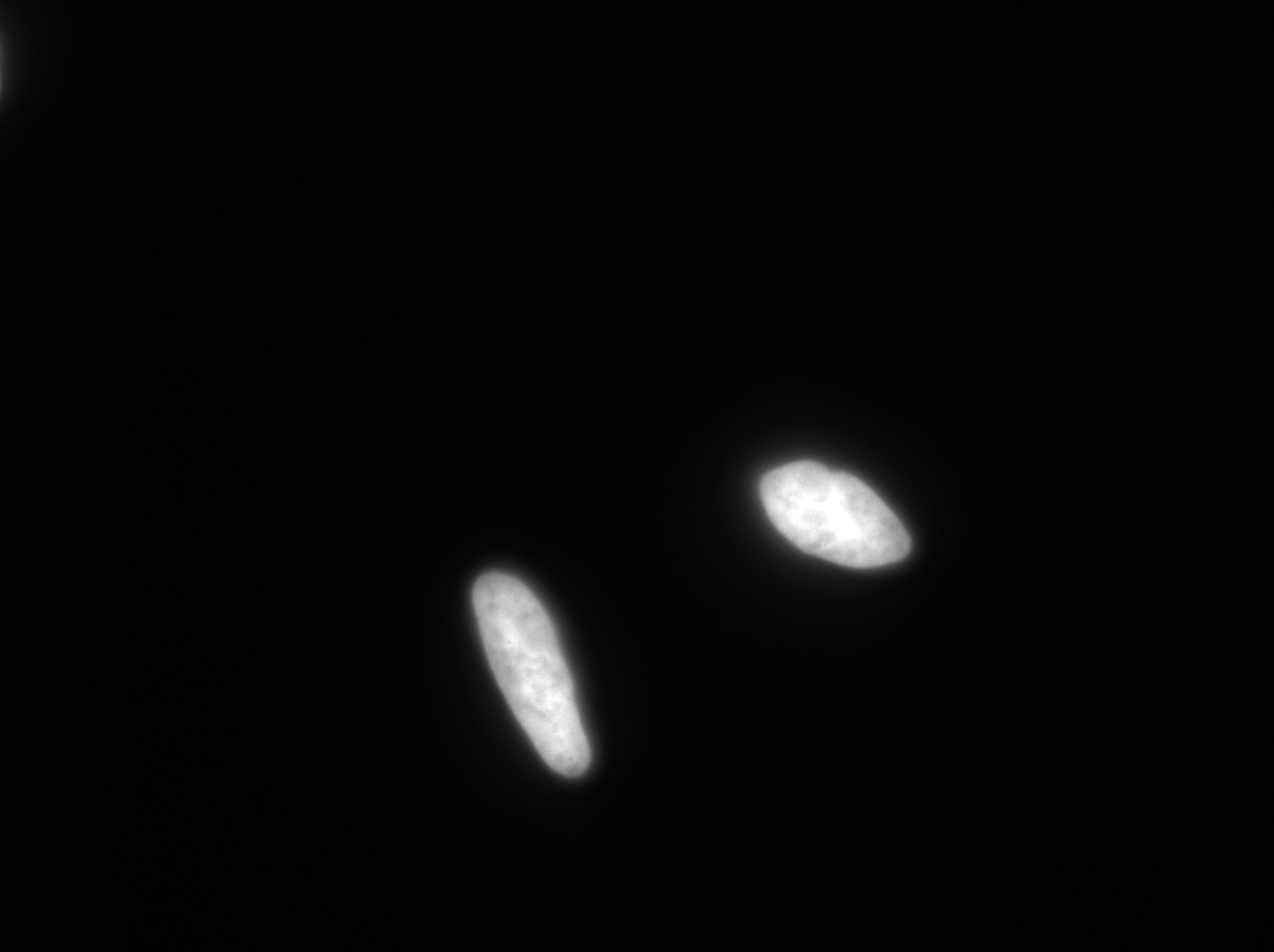

Supplement: Supplementary file 7 — Source Data [file 41467_2021_22575_MOESM7_ESM.zip › Raw data/Supplementary Figures/Supplementary Figure 1/Suppl Fig 1g/images IF/U2OSH33SNAP_newH33(CY3)_CPD(CY5)_w1DAPI.TIF]

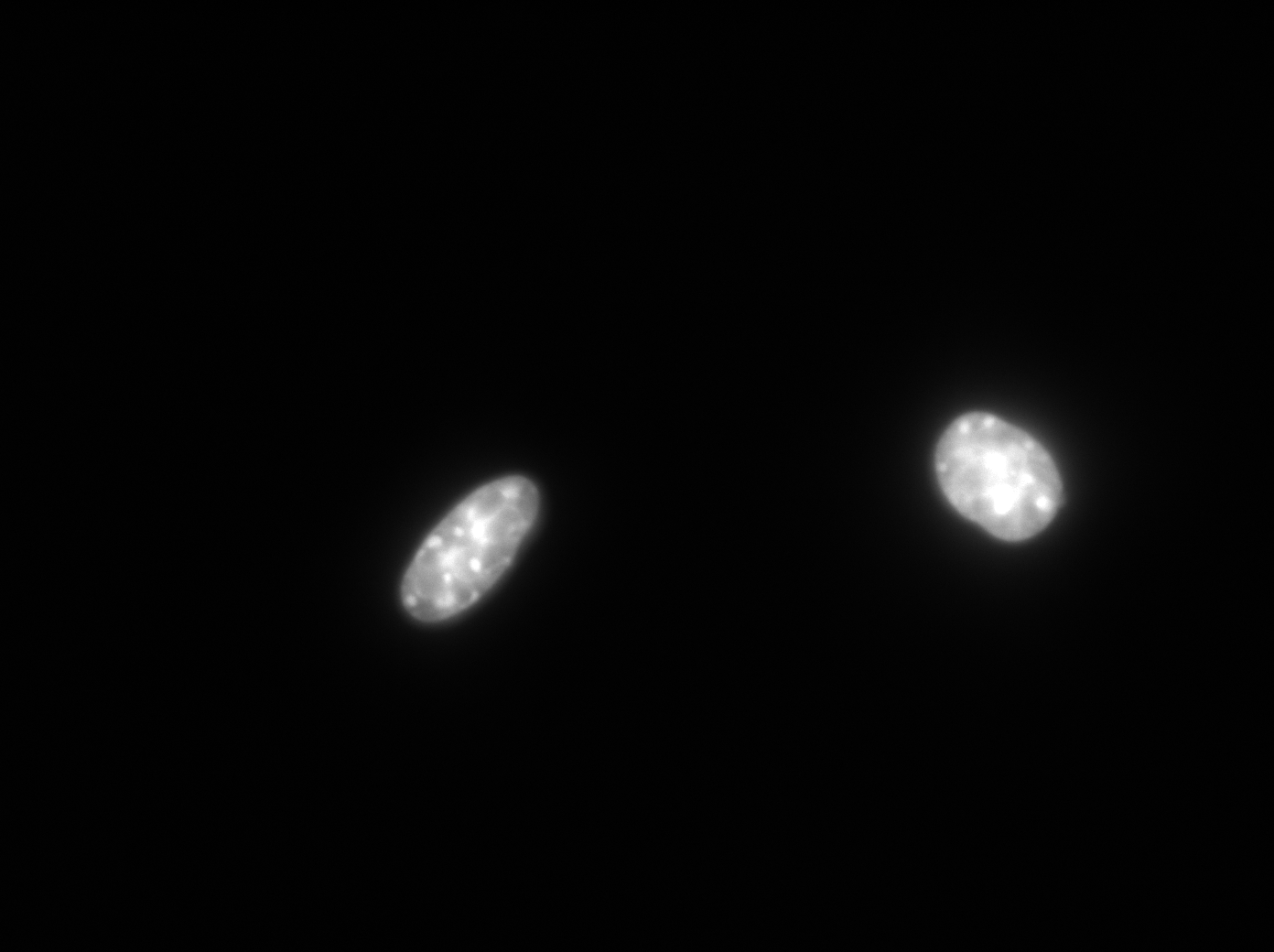

Supplement: Supplementary file 7 — Source Data [file 41467_2021_22575_MOESM7_ESM.zip › Raw data/Supplementary Figures/Supplementary Figure 1/Suppl Fig 1g/images IF/3T3GFPDDB2H33SNAP_newH33(CY3)_CPD(CY5)_w1DAPI.TIF]

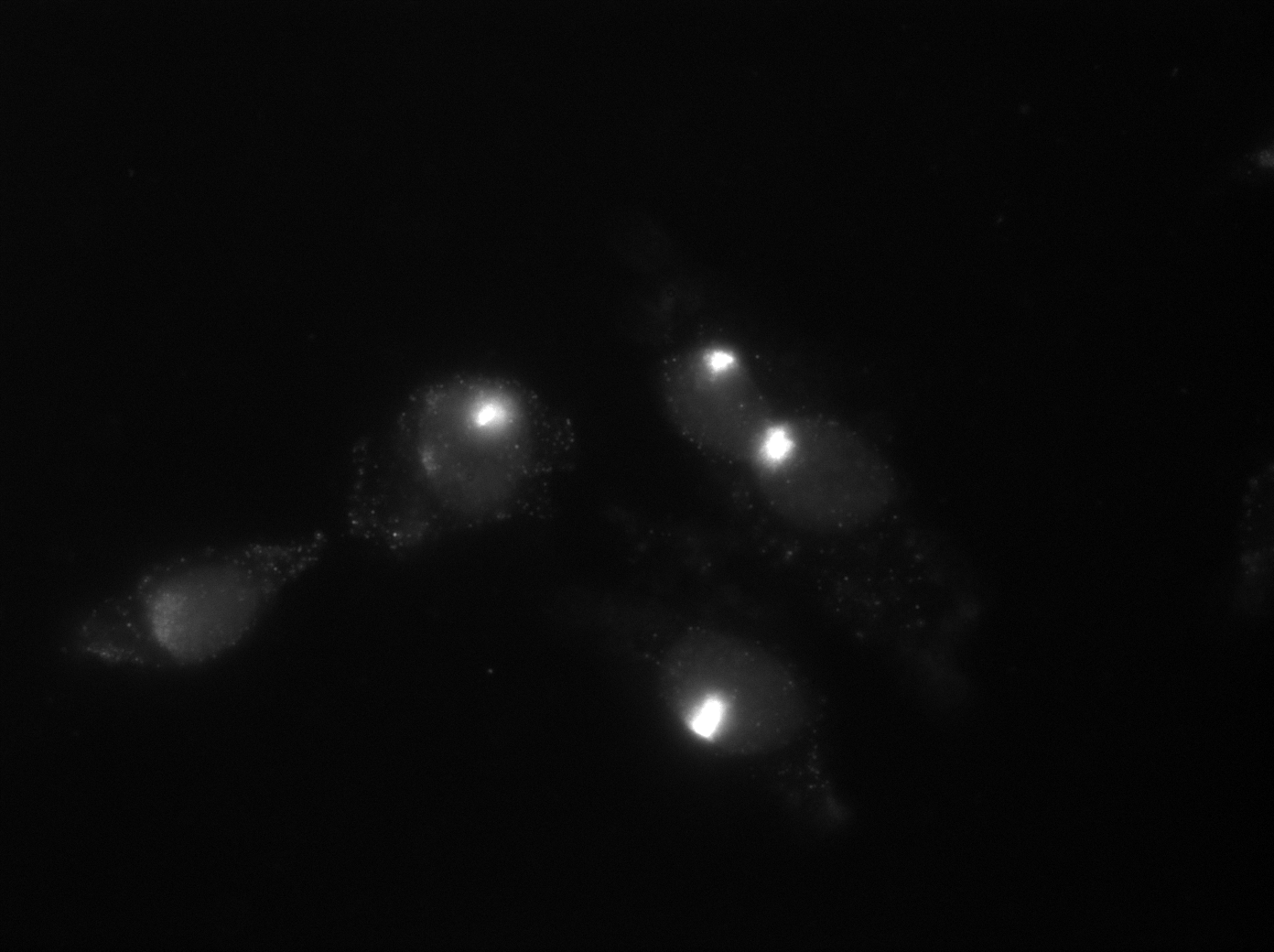

Supplement: Supplementary file 7 — Source Data [file 41467_2021_22575_MOESM7_ESM.zip › Raw data/Supplementary Figures/Supplementary Figure 1/Suppl Fig 1g/images IF/3t3_5_h33snap_newhistones(CY3)_cpd(CY5)_6_w4CY5.TIF]

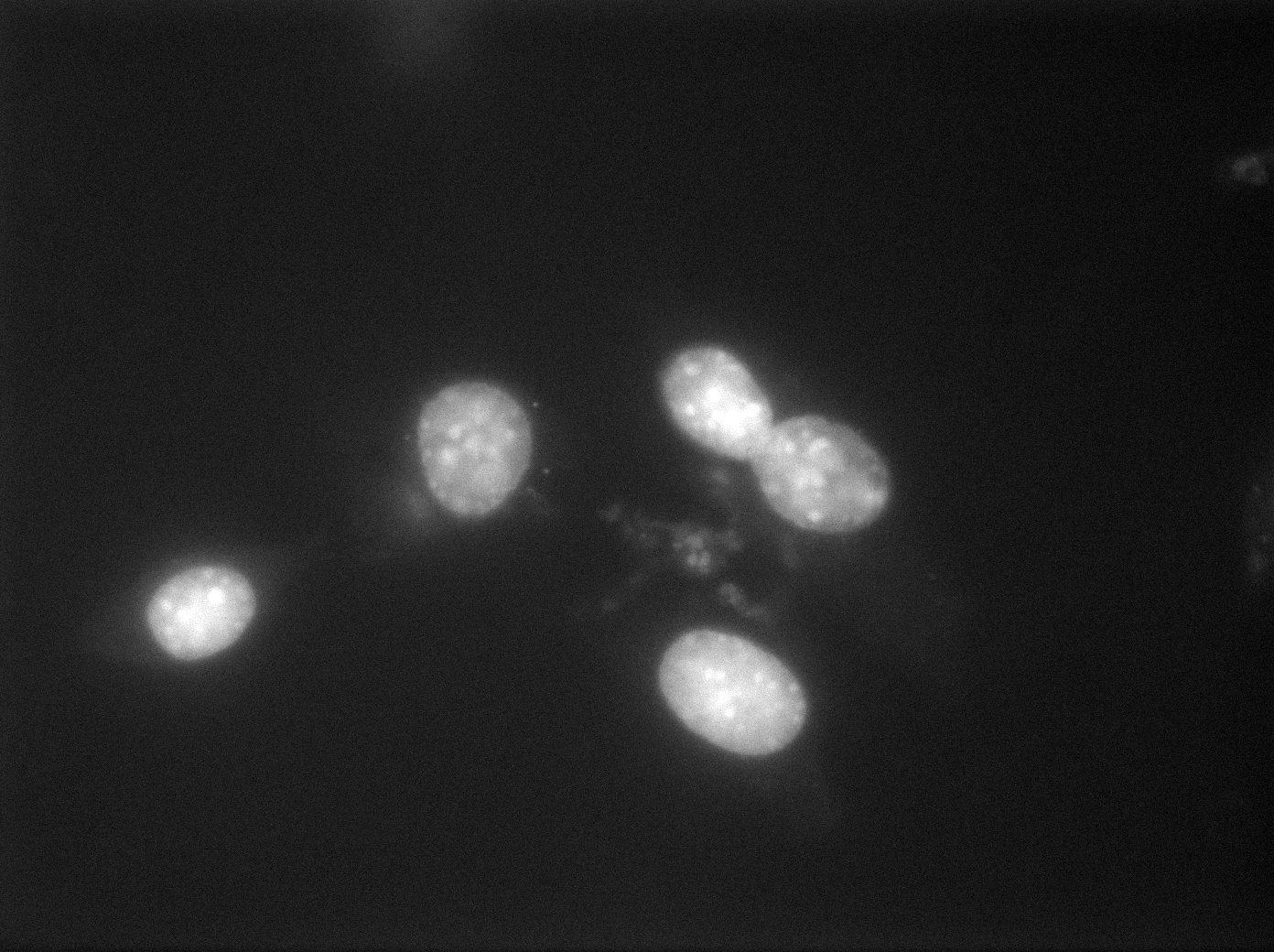

Supplement: Supplementary file 7 — Source Data [file 41467_2021_22575_MOESM7_ESM.zip › Raw data/Supplementary Figures/Supplementary Figure 1/Suppl Fig 1g/images IF/3t3_5_h33snap_newhistones(CY3)_cpd(CY5)_6_w2GFP.TIF]

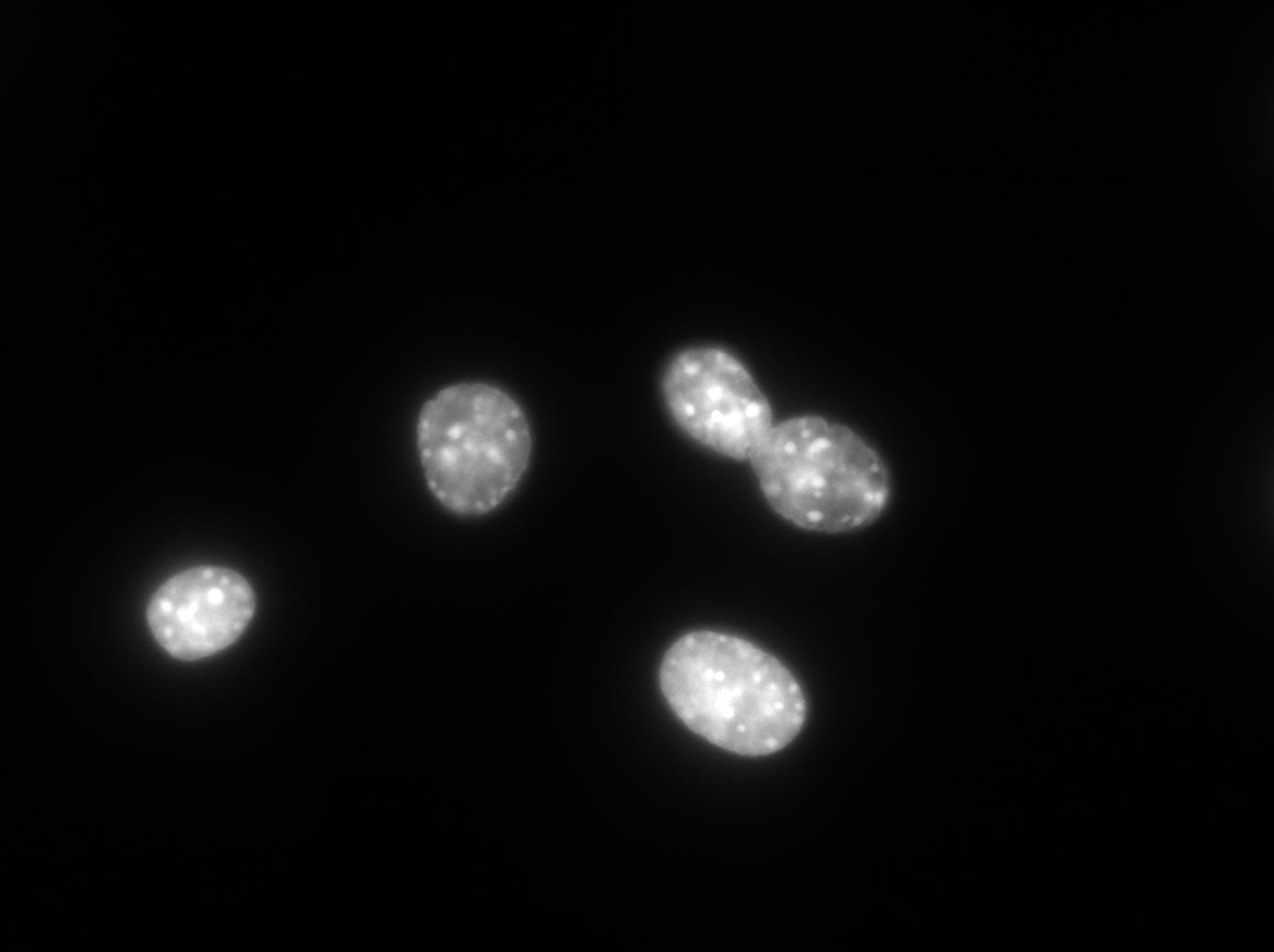

Supplement: Supplementary file 7 — Source Data [file 41467_2021_22575_MOESM7_ESM.zip › Raw data/Supplementary Figures/Supplementary Figure 1/Suppl Fig 1g/images IF/3t3_5_h33snap_newhistones(CY3)_cpd(CY5)_6_w1DAPI.TIF]

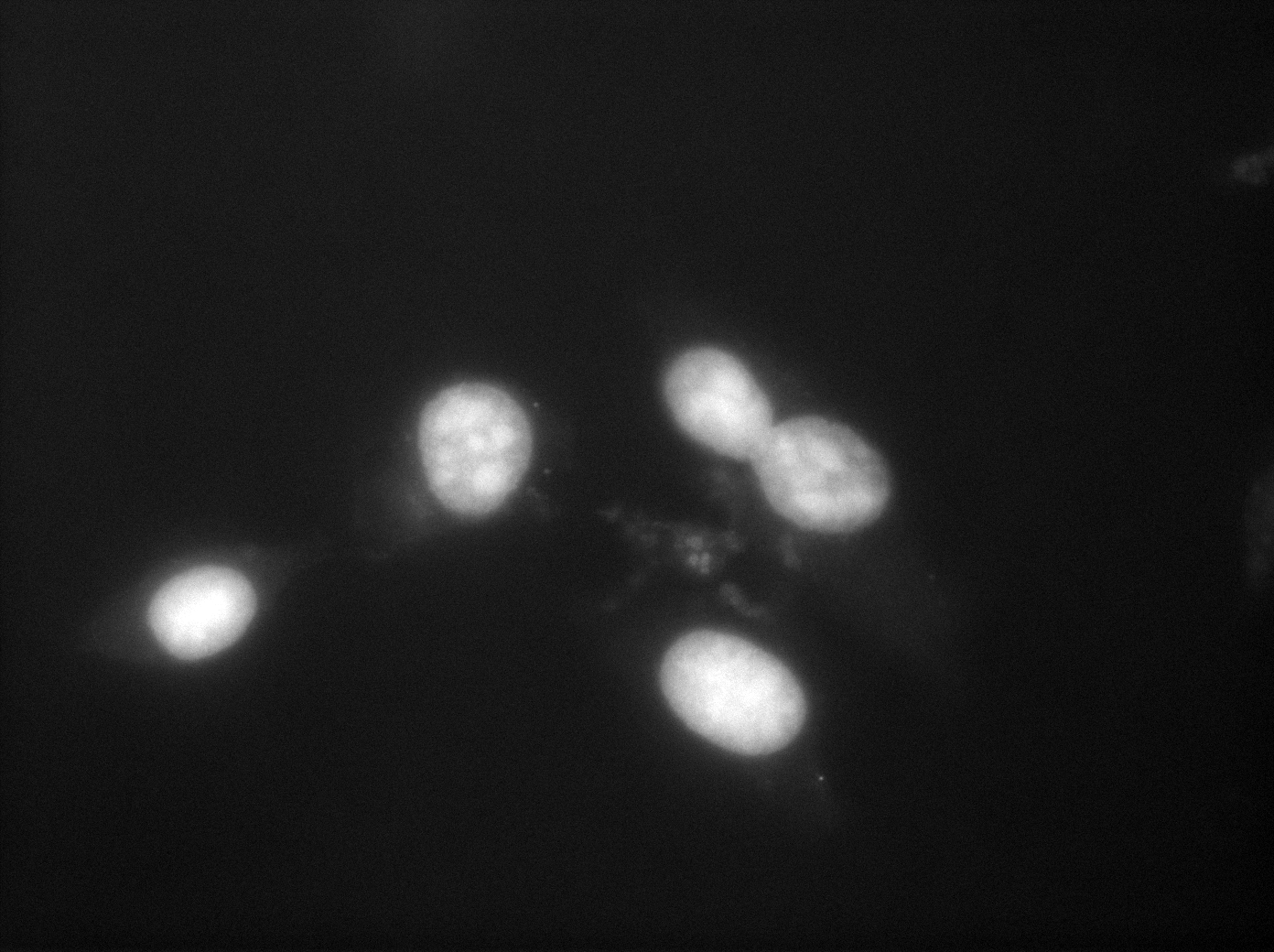

Supplement: Supplementary file 7 — Source Data [file 41467_2021_22575_MOESM7_ESM.zip › Raw data/Supplementary Figures/Supplementary Figure 1/Suppl Fig 1g/images IF/3t3_5_h33snap_newhistones(CY3)_cpd(CY5)_6_w3CY3.TIF]

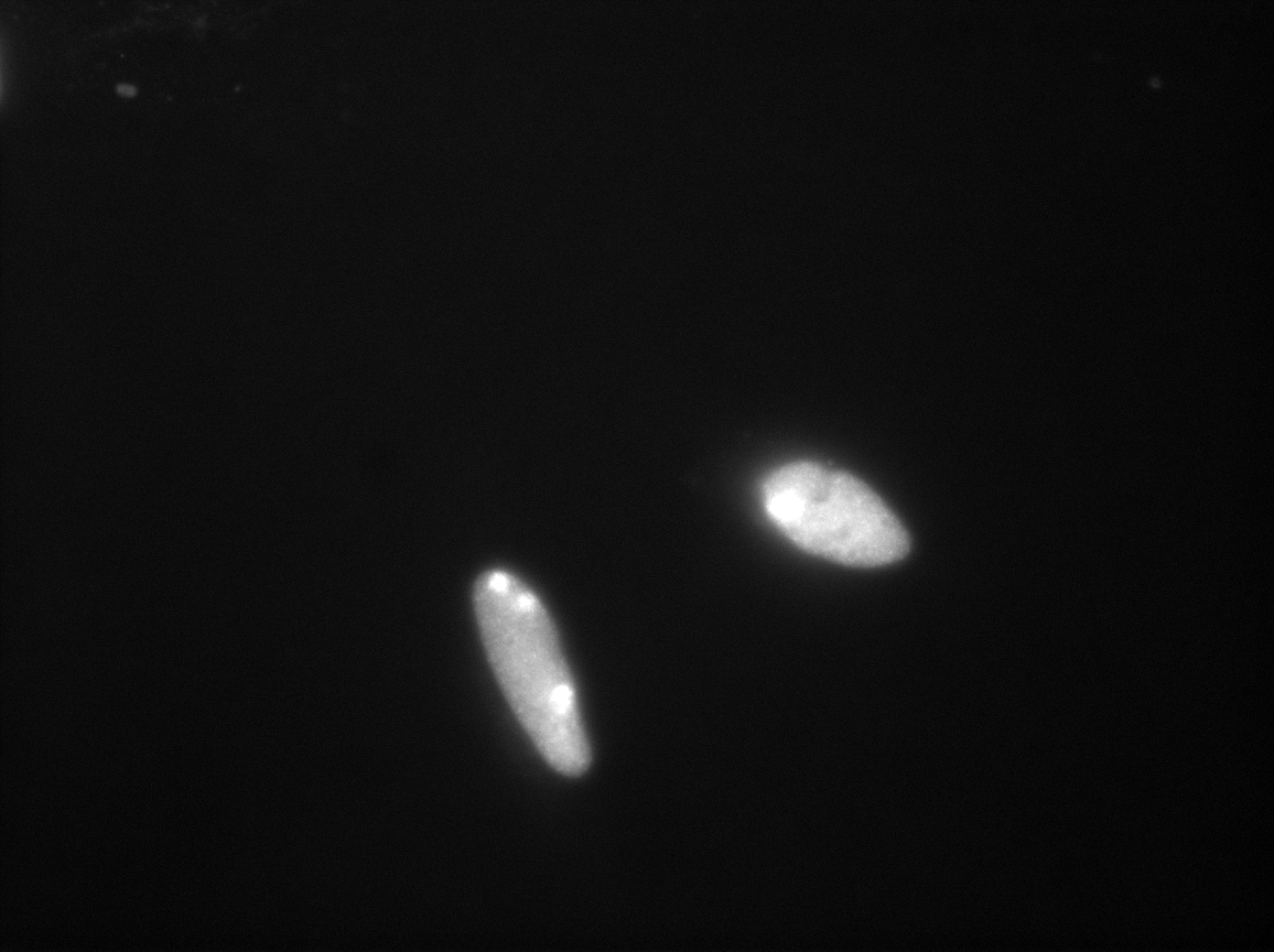

Supplement: Supplementary file 7 — Source Data [file 41467_2021_22575_MOESM7_ESM.zip › Raw data/Supplementary Figures/Supplementary Figure 1/Suppl Fig 1g/images IF/U2OSH33SNAP_newH33(CY3)_CPD(CY5)_w3CY3.TIF]

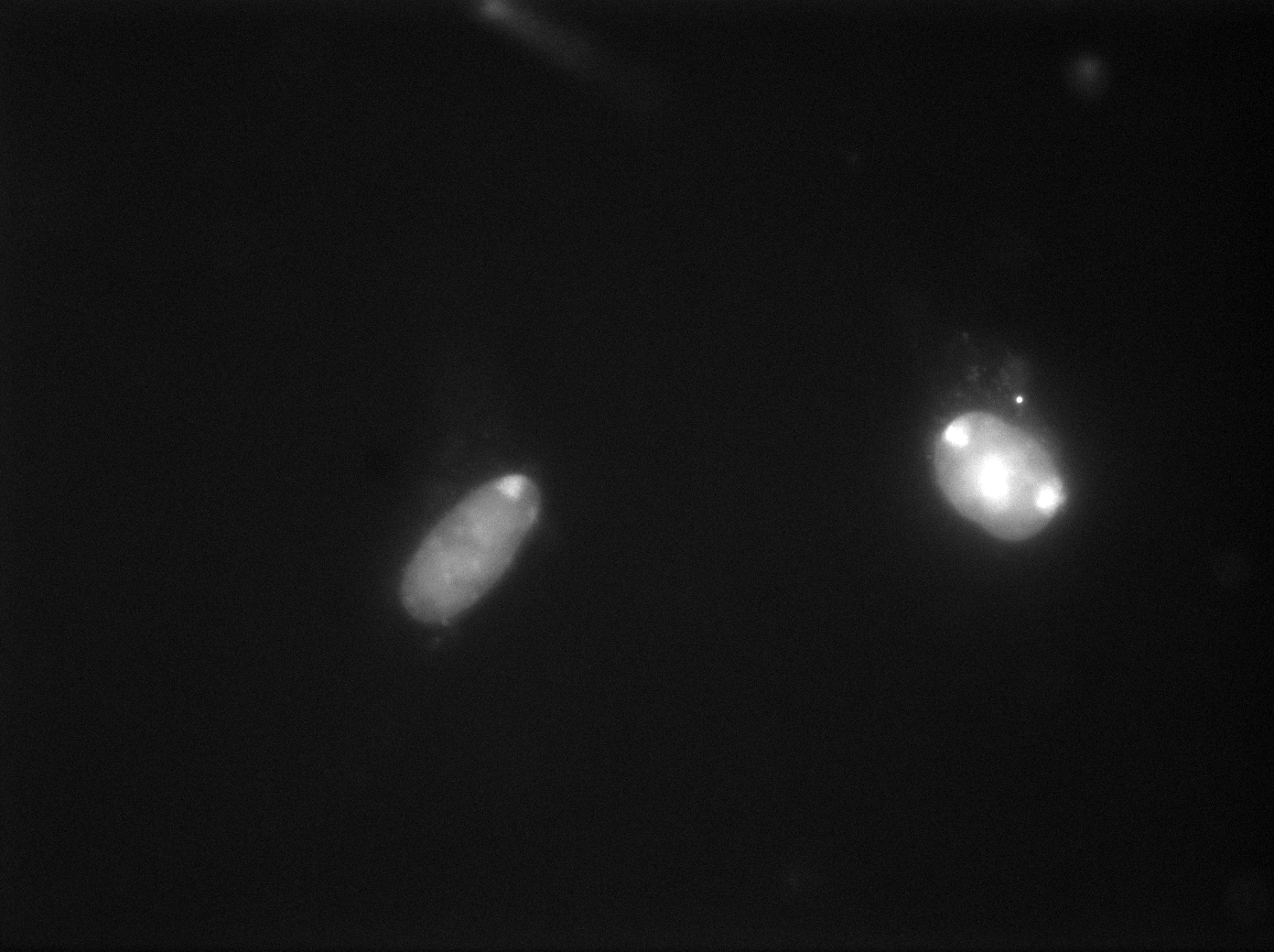

Supplement: Supplementary file 7 — Source Data [file 41467_2021_22575_MOESM7_ESM.zip › Raw data/Supplementary Figures/Supplementary Figure 1/Suppl Fig 1g/images IF/3T3GFPDDB2H33SNAP_newH33(CY3)_CPD(CY5)_w2GFP.TIF]

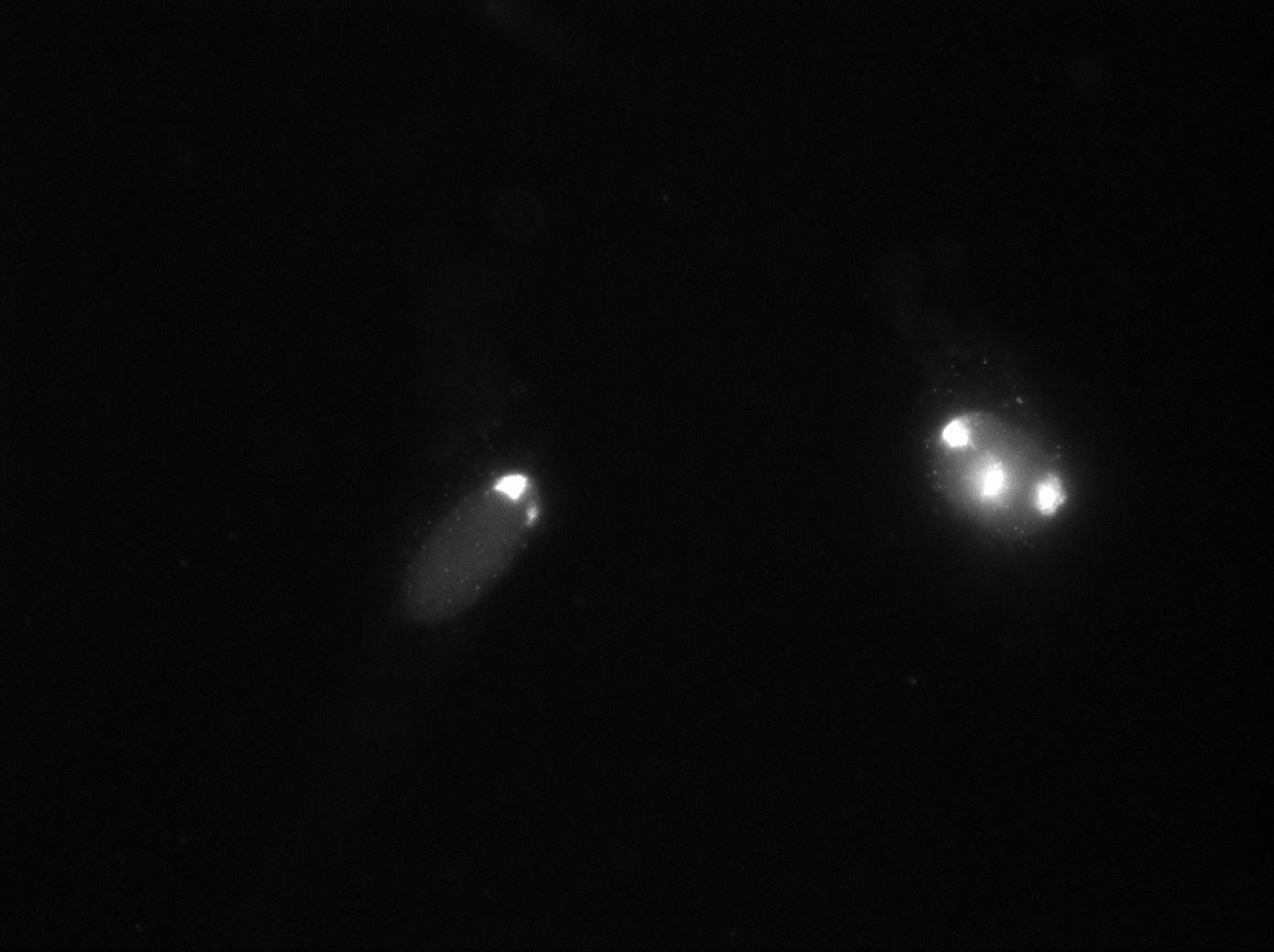

Supplement: Supplementary file 7 — Source Data [file 41467_2021_22575_MOESM7_ESM.zip › Raw data/Supplementary Figures/Supplementary Figure 1/Suppl Fig 1g/images IF/3T3GFPDDB2H33SNAP_newH33(CY3)_CPD(CY5)_w4CY5.TIF]

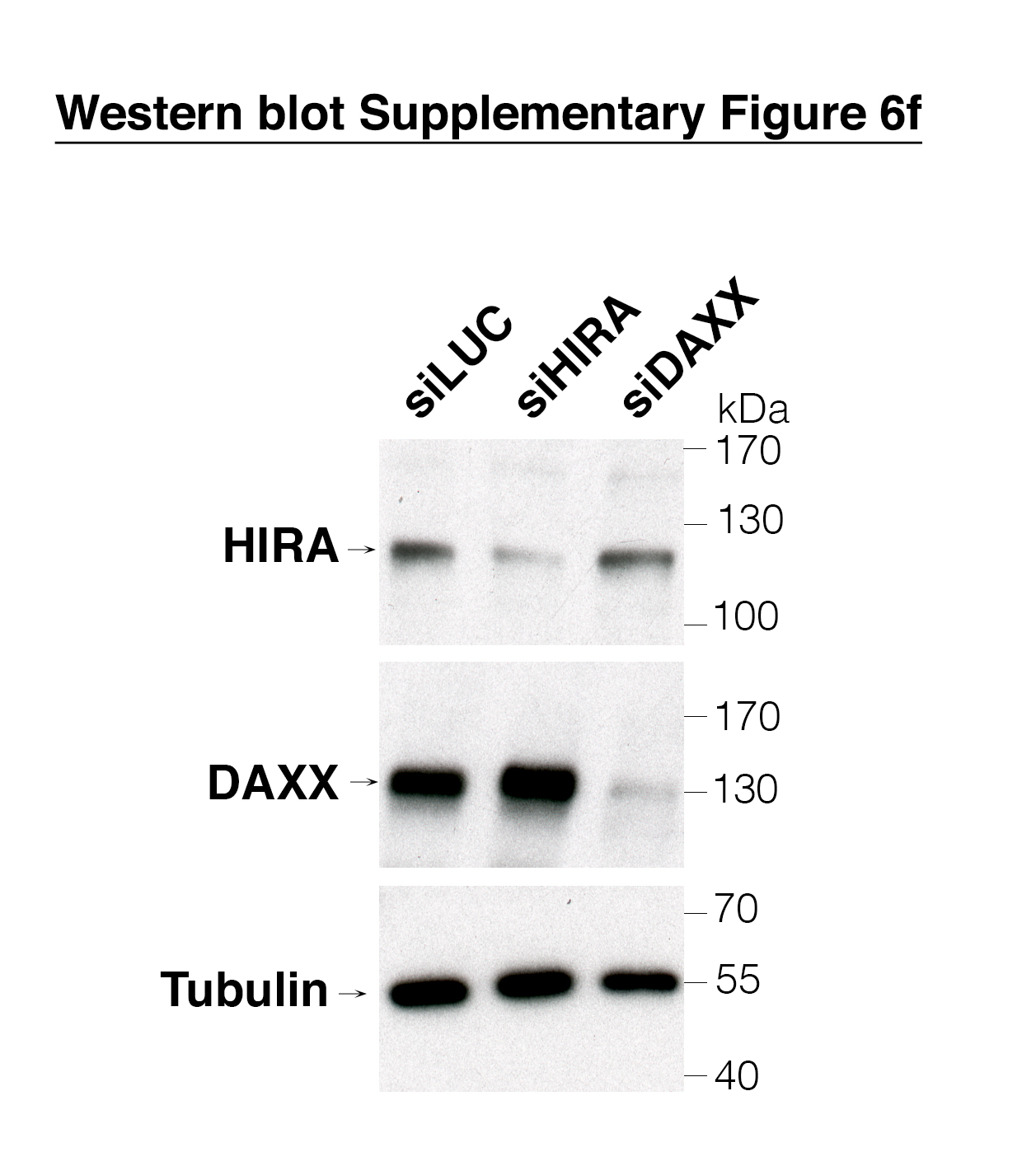

Supplement: Supplementary file 7 — Source Data [file 41467_2021_22575_MOESM7_ESM.zip › Raw data/Supplementary Figures/Supplementary Figure 6/Western blot GFP-DDB2 removal siHIRA_DAXX.tif]

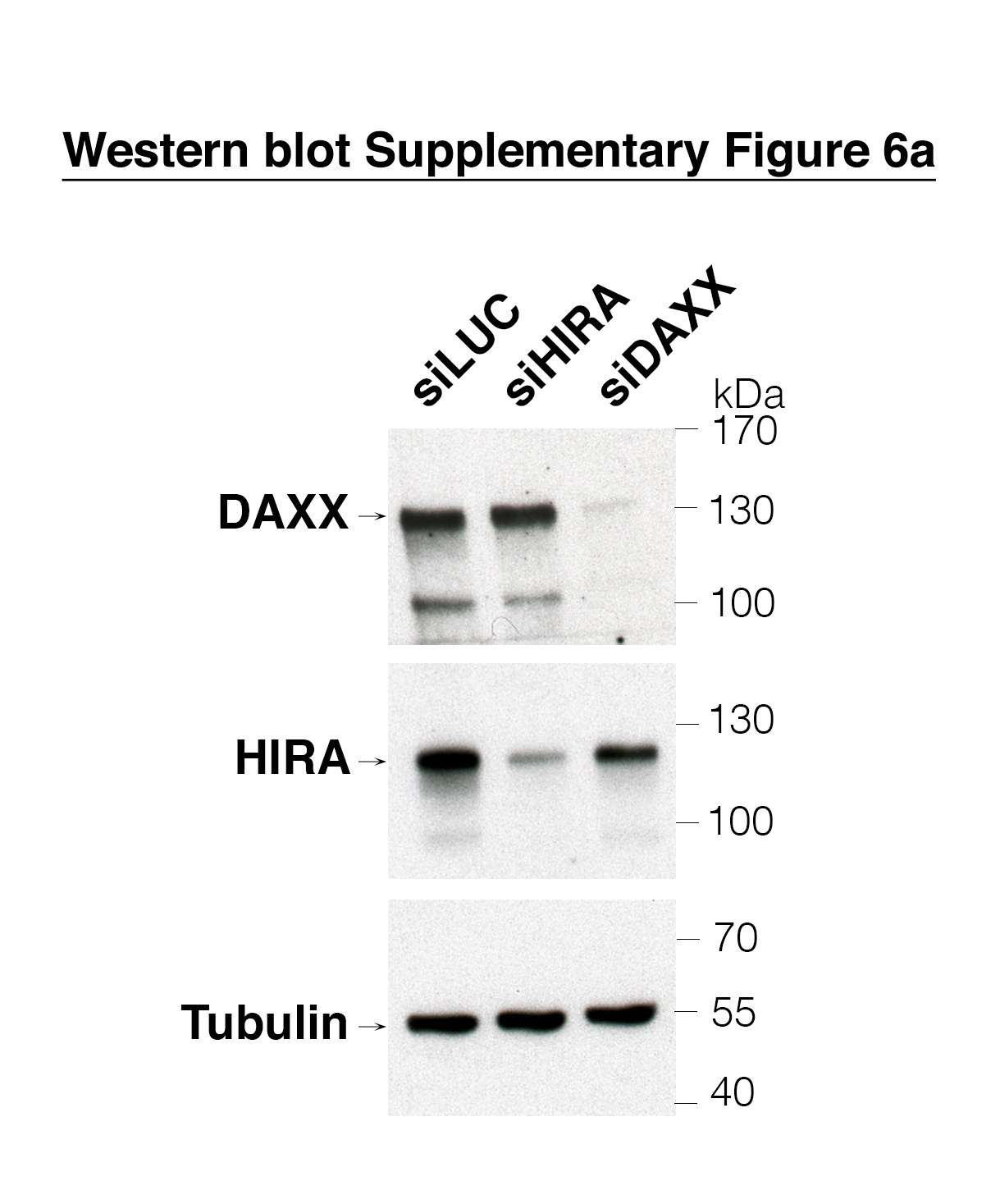

Supplement: Supplementary file 7 — Source Data [file 41467_2021_22575_MOESM7_ESM.zip › Raw data/Supplementary Figures/Supplementary Figure 6/Fig 6a/Western blot HIRA_DAXX.tif]

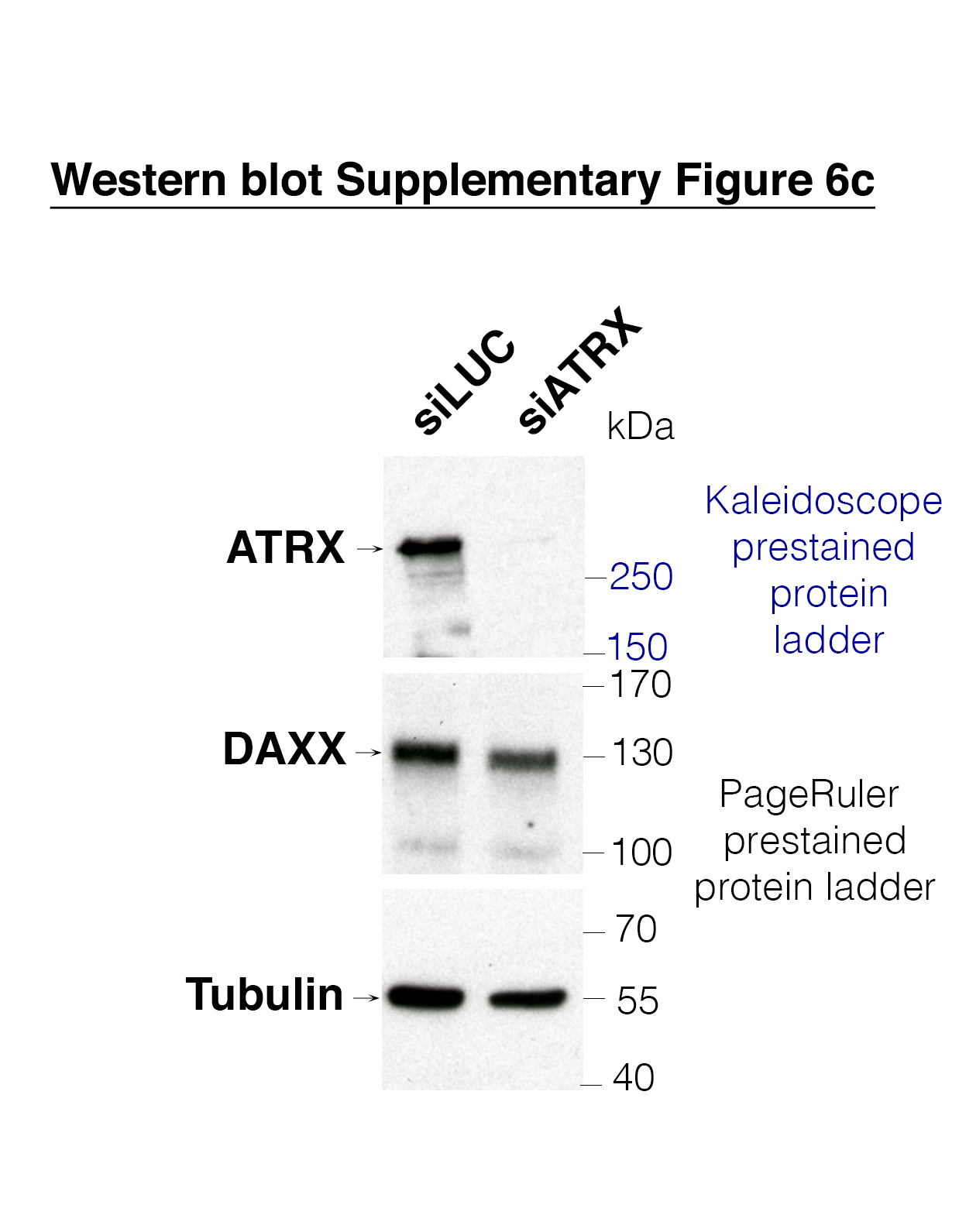

Supplement: Supplementary file 7 — Source Data [file 41467_2021_22575_MOESM7_ESM.zip › Raw data/Supplementary Figures/Supplementary Figure 6/Fig 6c/Western blot DAXX_siATRX.tif]

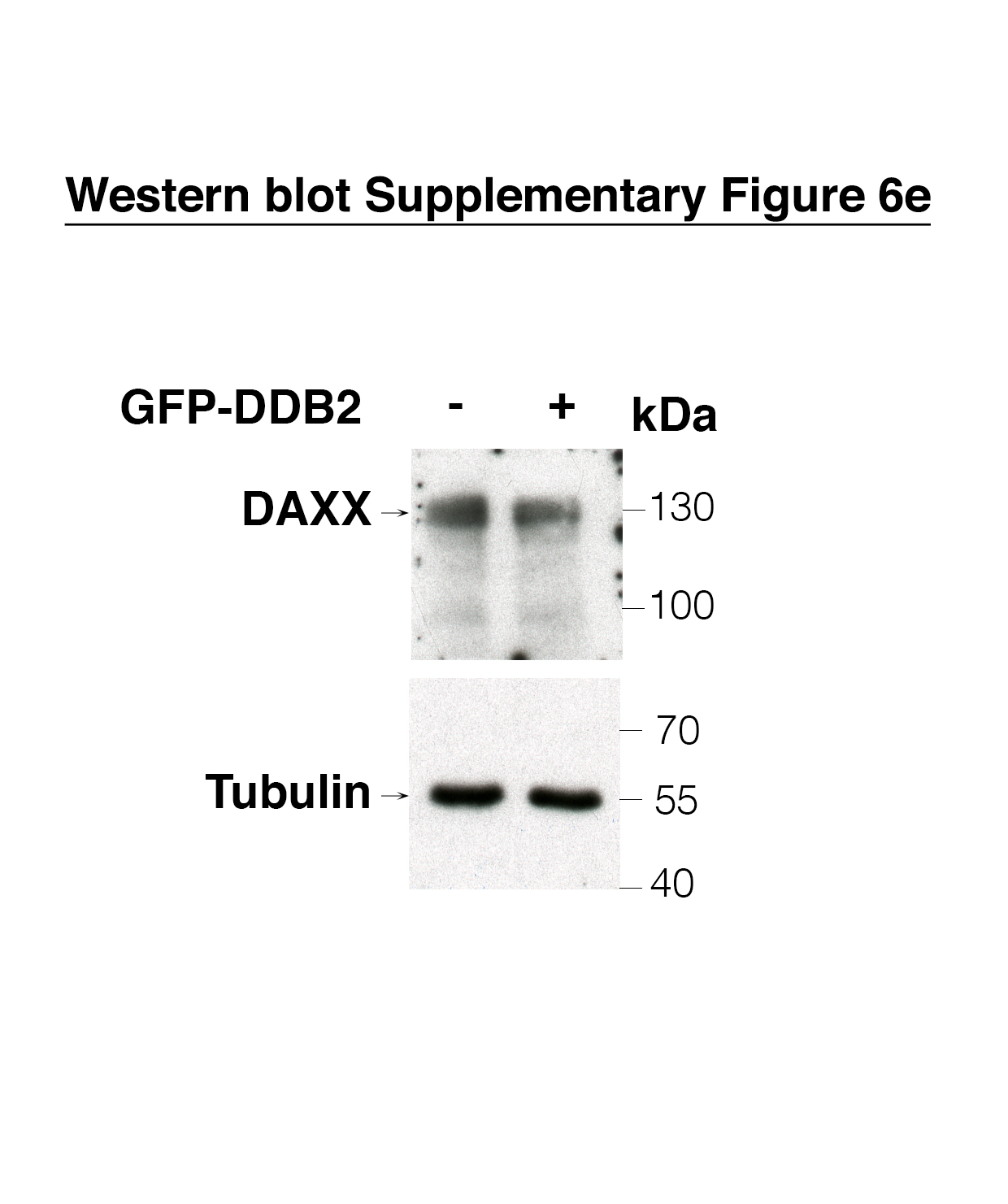

Supplement: Supplementary file 7 — Source Data [file 41467_2021_22575_MOESM7_ESM.zip › Raw data/Supplementary Figures/Supplementary Figure 6/Fig 6e/Western blot DAXX empty vs GFPDDB2.tif]

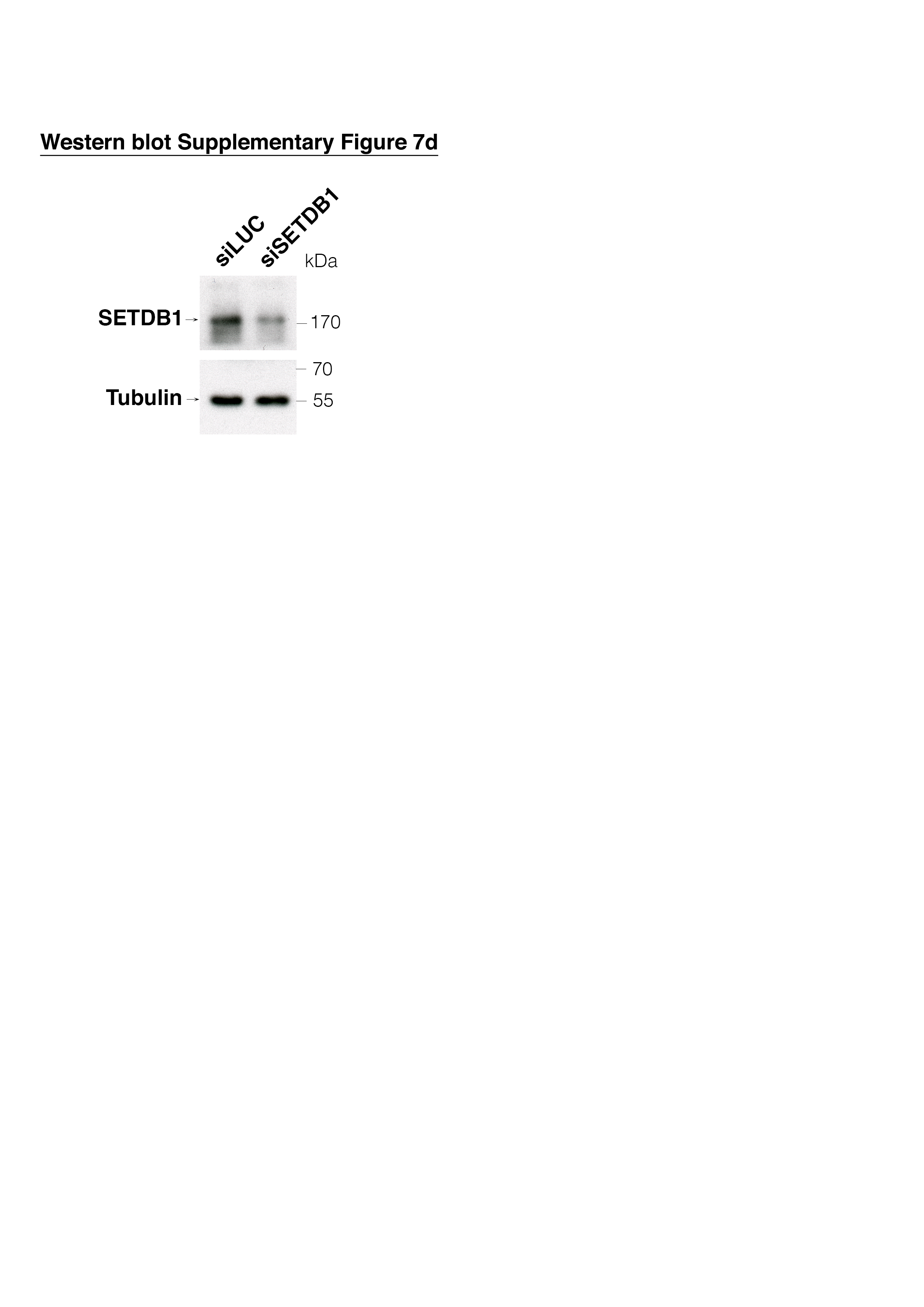

Supplement: Supplementary file 7 — Source Data [file 41467_2021_22575_MOESM7_ESM.zip › Raw data/Supplementary Figures/Supplementary Figure 7/Suppl Fig 7d/Western blot GFP-DDB2 removal siSETDB1 (raw data).tif]

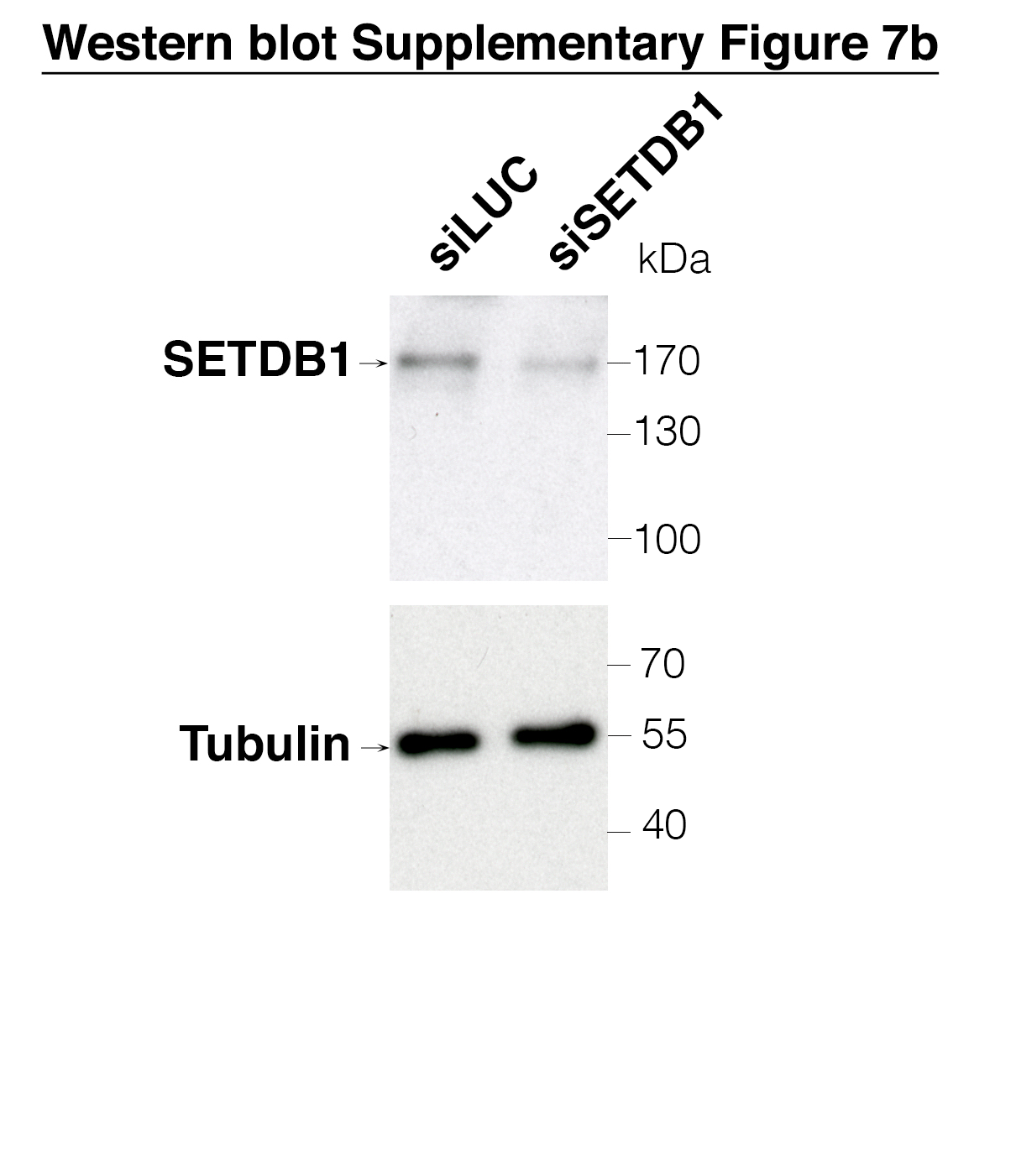

Supplement: Supplementary file 7 — Source Data [file 41467_2021_22575_MOESM7_ESM.zip › Raw data/Supplementary Figures/Supplementary Figure 7/Suppl Fig 7b/Western blot H3K9me3 siSETDB1.tif]

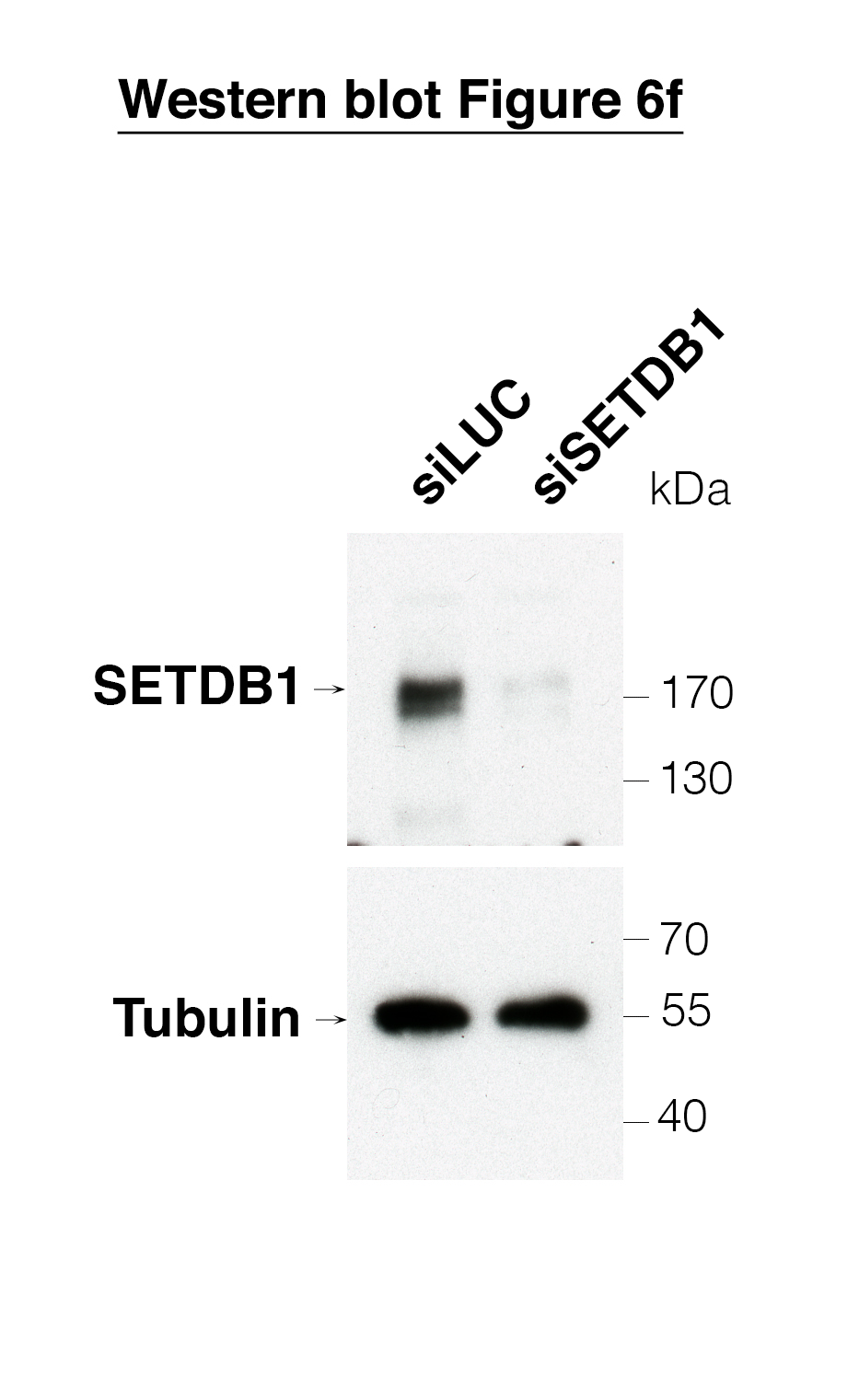

Supplement: Supplementary file 7 — Source Data [file 41467_2021_22575_MOESM7_ESM.zip › Raw data/Main Figures/Figure 6/Fig 6f/Western blot micronuclei.tif]

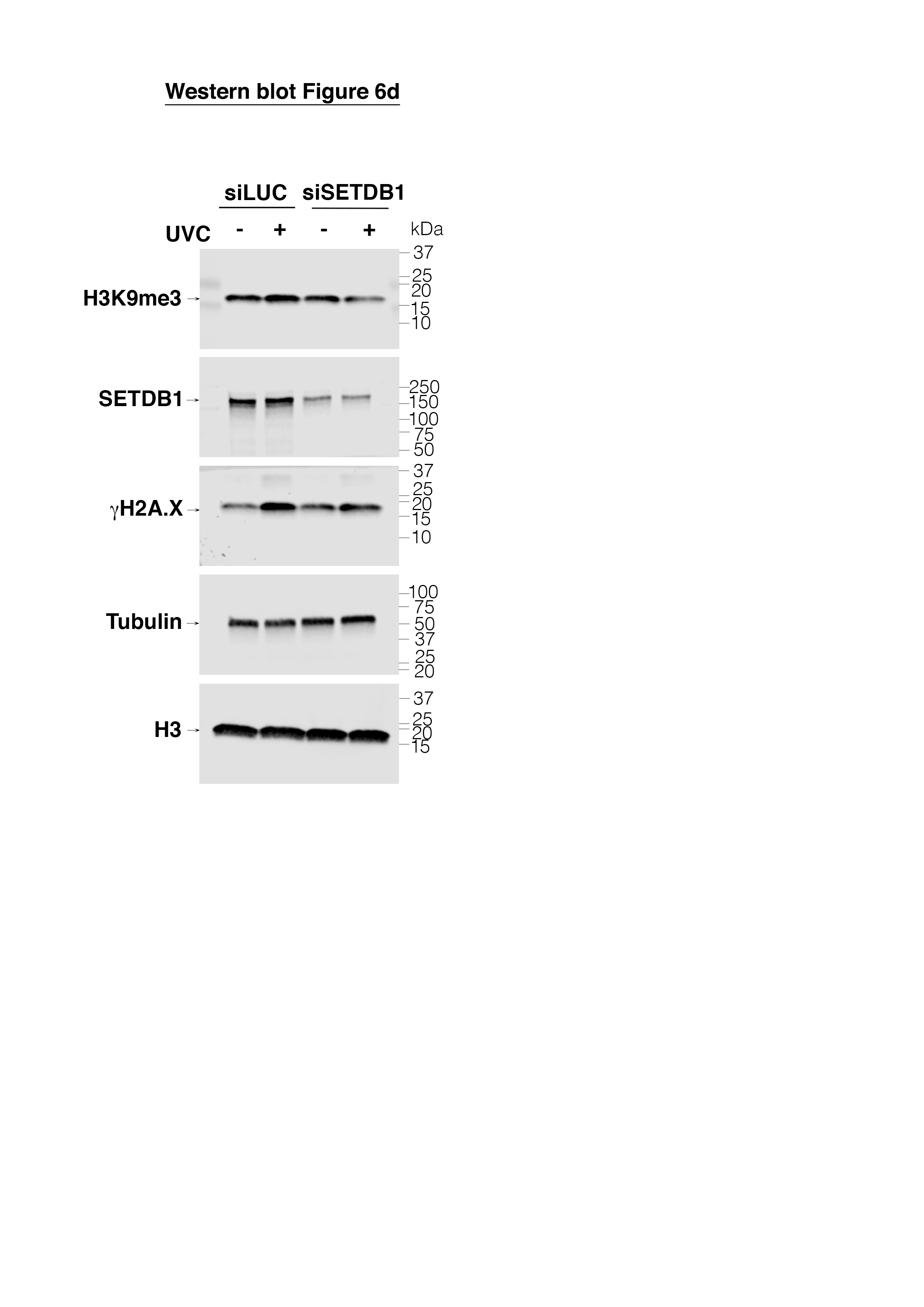

Supplement: Supplementary file 7 — Source Data [file 41467_2021_22575_MOESM7_ESM.zip › Raw data/Main Figures/Figure 6/Fig 6d/Western blot H3K9me3.tif]

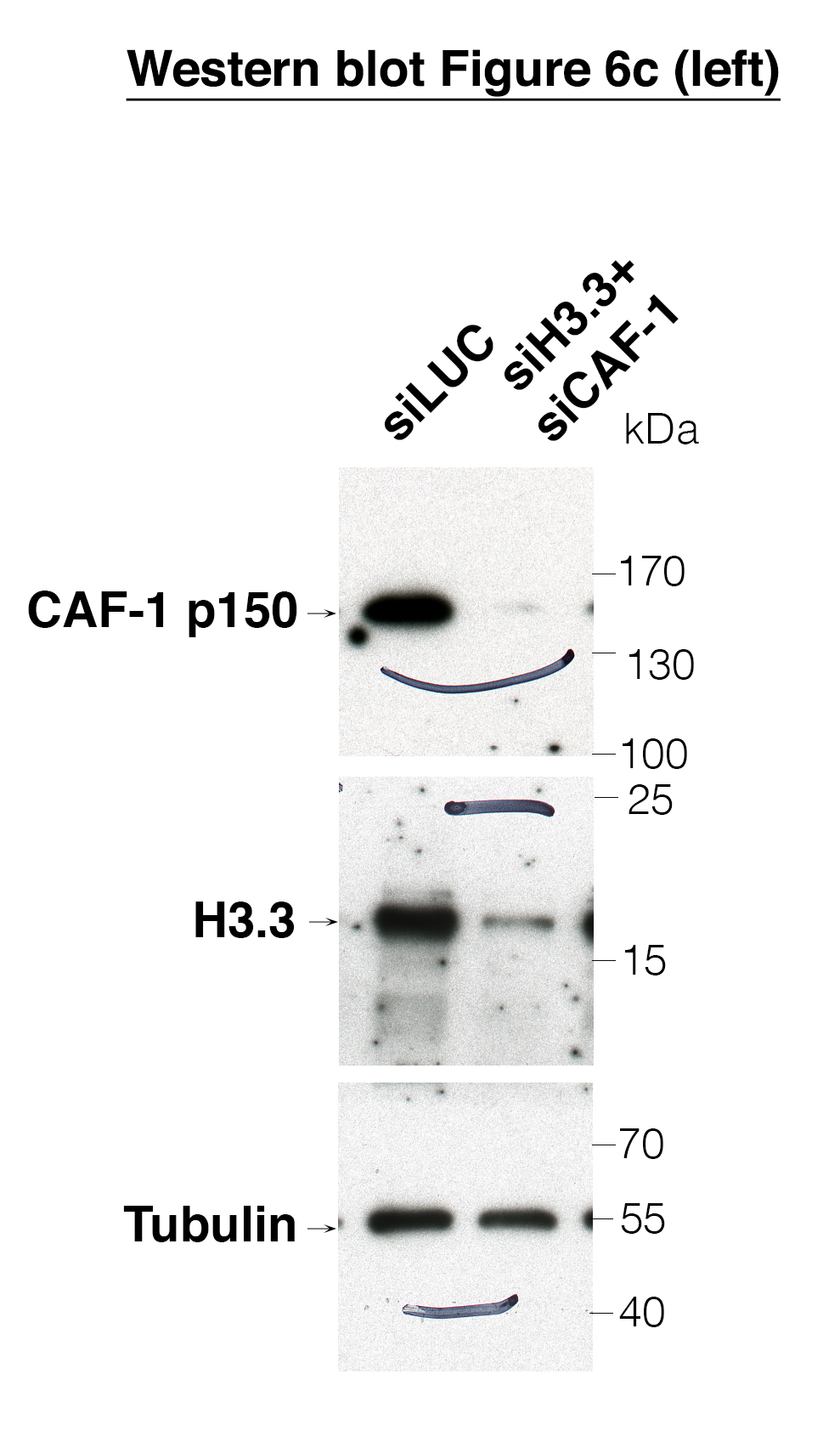

Supplement: Supplementary file 7 — Source Data [file 41467_2021_22575_MOESM7_ESM.zip › Raw data/Main Figures/Figure 6/Fig 6c/Western blot siH3.3-CAF1.tif]

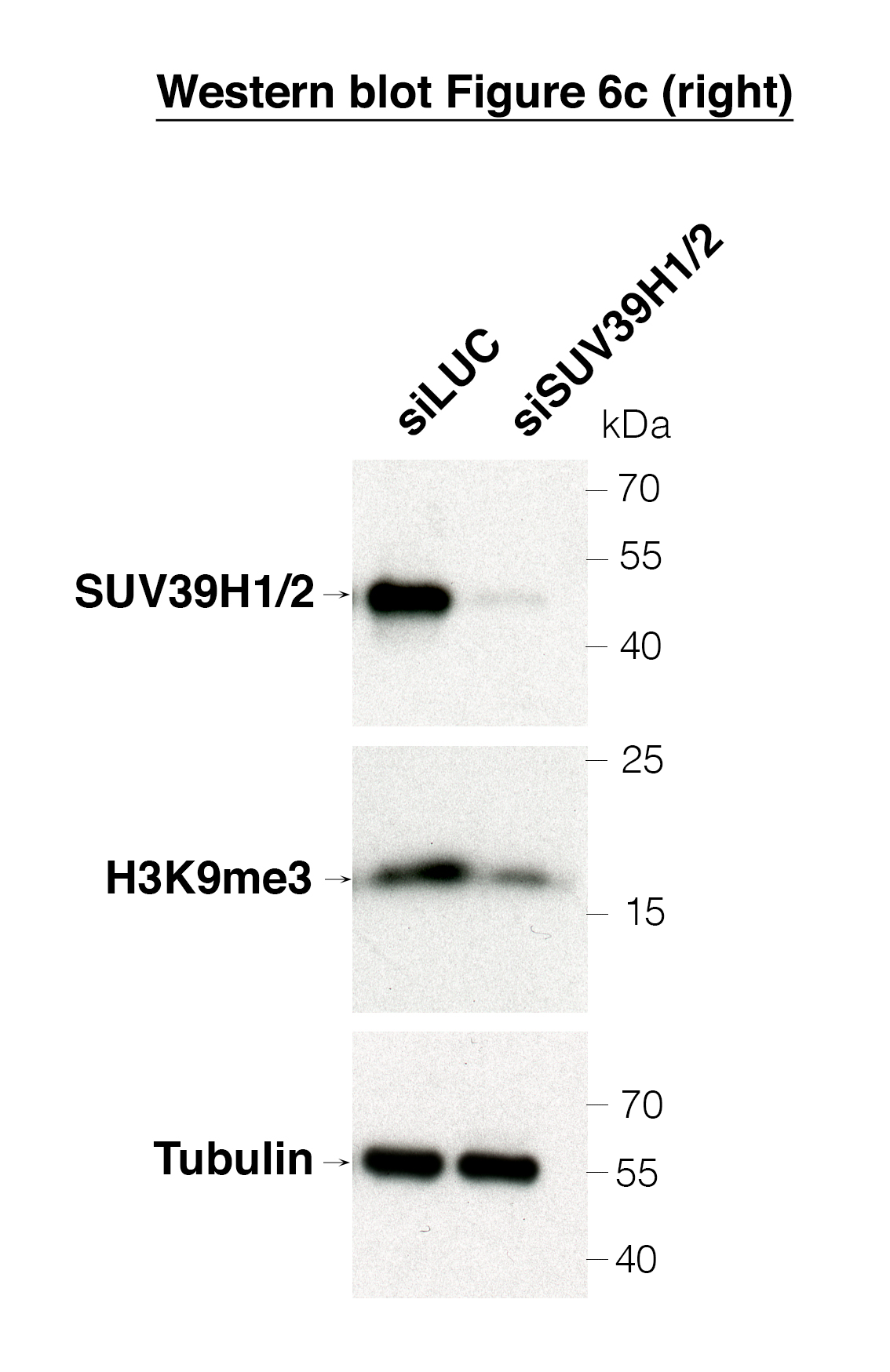

Supplement: Supplementary file 7 — Source Data [file 41467_2021_22575_MOESM7_ESM.zip › Raw data/Main Figures/Figure 6/Fig 6c/Western blot siSUV39H1-2.tif]

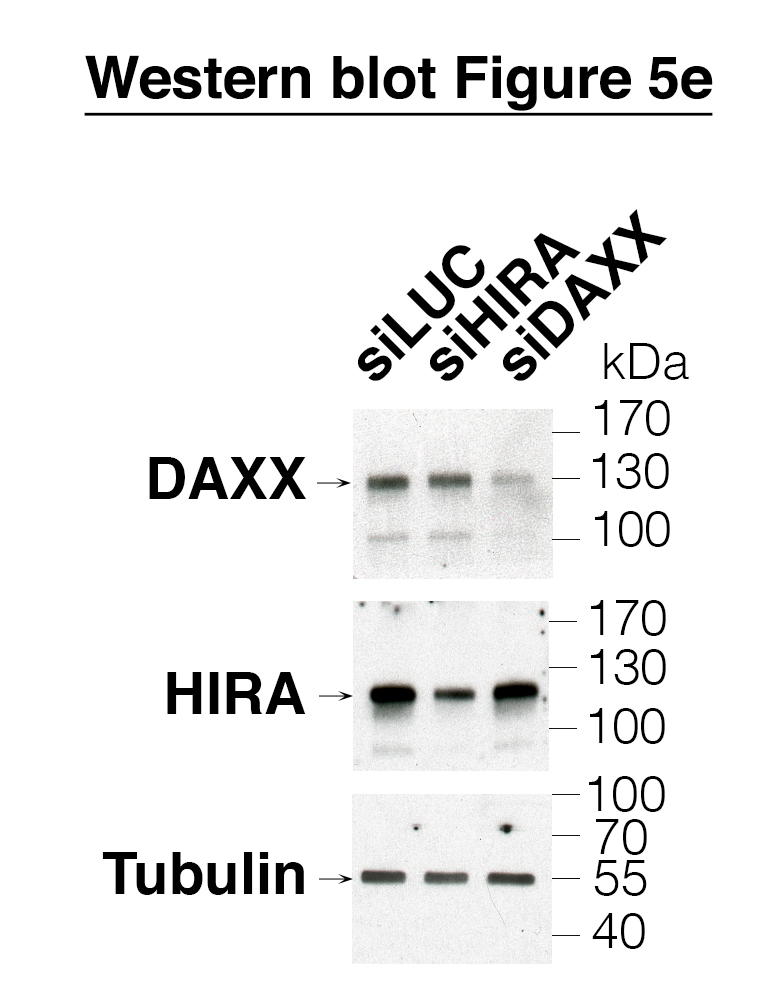

Supplement: Supplementary file 7 — Source Data [file 41467_2021_22575_MOESM7_ESM.zip › Raw data/Main Figures/Figure 5/Fig 5e/Western blot siHIRA, siDAXX.tif]
